# Supplementary material for: Acidity suppresses CD8 + T-cell function by perturbing IL-2, mTORC1, and c-Myc signaling
Source: EMBO J. 2024 Sep 16;43(21):8. doi: 10.1038/s44318-024-00235-w (PMC11535206; doi:10.1038/s44318-024-00235-w)
Supplement: Supplementary file 1 — Appendix [file 44318_2024_235_MOESM1_ESM.pdf]

Appendix for

Acidity suppresses CD8+ T-cell function by perturbing IL-2, mTORC1, and c-Myc signaling

by Vuillefroy de Silly et al.

Appendix Figure S1. Acidity lowers IL-2-dependent response of CTLs. (page 2-3)

Appendix Figure S2. Acidity lowers IL-2R signaling in CTLs. (page 4-5)

Appendix Figure S3. Impact of pH on pathways that may act on mTORC1 and c-Myc. (page 6)

Appendix Figure S4. Impact of pH on pathways that may act on mTORC1 and c-Myc. (page 7)

Appendix Figure S5. mTORC1 and c-Myc pathways are lowered at low pH. (page 8)

Appendix Figure S6. The impact of acidity on mTORC1 and c-Myc does not seem to be linked to a disturbance in the activation of upstream IL-2R signaling transducers in polyclonal CTLs. (page 9-10)

Appendix Figure S7. The Switch IL-2 mutant does not improve mTORC1 signaling, c-Myc levels or T-cell expansion at low pH of murine CD8+ T cells. (page 11)

Appendix Figure S8. The Switch IL-2 mutant does not improve mTORC1 signaling, c-Myc levels or T-cell expansion at low pH of human CD8+ T cells. (page 12)

Appendix Figure S9. Knocking out CD25 reveals that it is not involved in the pattern observed at low pH. (page 13-14)

Appendix Figure S10. Glutamine metabolism exploration at low pH. (page 15)

Appendix Figure S11. Methodology to estimate mean cell division number. (page 16)

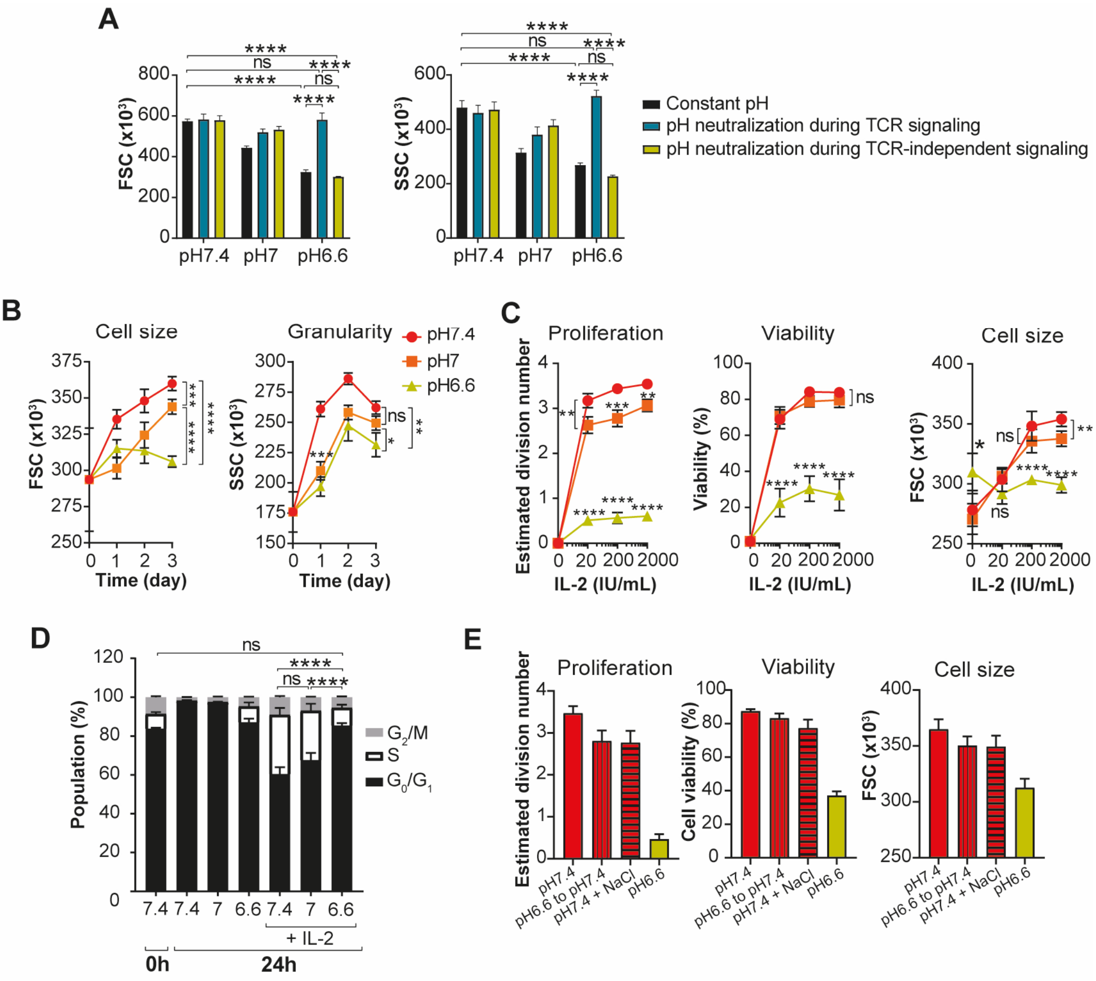

Appendix Figure S1. Acidity lowers IL-2-dependent response of CTLs.

(A) Acidity influences cell size and granularity of CTLs during TCR signaling. OT-I CTLs were cultured for one day in anti-CD3 –coated plates (TCR signaling step). The resulting cells and supernatants were transferred to an anti-CD3 –free plate and cultured for two further days (TCR-independent signaling step). Cells were either: cultured during the TCR signaling step and the TCR-independent signaling at constant pH (“Constant pH”), cultured at pH7.4 during the TCR signaling step then at various pH during the TCR-independent signaling step (“pH neutralization during TCR signaling”), or cultured at various pH during TCR signaling step then at pH7.4 during the TCR-independent signaling step (“pH neutralization during TCR-independent signaling”). Bar graph shows the mean cell size or granularity + SEM of four biological replicates from two independent experiments. ns: not statistically significant, ****p<0.0001 (one-way repeated measures ANOVA, Tukey post-hoc test).

(B) Time-course of the pH impact on the cell size and granularity of CTLs to IL-2. OT-I CTLs were cultured in the presence of exogenous murine IL-2. Results show the mean cell size, or granularity, ± SEM of at least three biological replicates from at least two independent experiments. ns: not significant, *p<0.05, **p<0.01, ***p<0.001, ****p<0.0001 (Student’s paired *t*-test).

(**C**) Impact of pH on the dose-response of CTLs to IL-2. OT-I CTLs were cultured for three days. Results show the estimated division number, viability or cell size ± SEM of at least seven biological replicates from at least three independent experiments. ns: not significant, *p<0.05, **p<0.01, ***p<0.001, ****p<0.0001 (Student’s paired *t*-test).

(**D**) Impact of pH on cell cycle phases of OT-I CTLs in response to IL-2. Results show the mean proportion of cells in G_0/G_1 (black solid bars), S (black empty bars), or G_2/M (grey solid bars) phases as evidenced by propidium iodide staining + SEM of four biological replicates from two independent

experiments. ns: not significant, ****p<0.0001 (one-way repeated measures ANOVA, Tukey post-hoc test): calculated for the S phase.

(**E**) Impact of acidity on CTL response to IL-2 is not due to precipitation/inactivation of molecules in the medium or to increased osmolarity. OT-I CTLs were cultured for three days in the presence of exogenous murine IL-2. The conditions “pH6.6 to pH7.4” refers to the use of medium that was beforehand acidified at pH6.6, incubated for two hours at 37°C and neutralized with NaOH to pH7.4, while “NaCl” means that CTLs were cultured at pH7.4 in the presence of the same amounts of NaCl than the HCl concentrations (around 31 mM) used to reach pH6.6. Results show the estimated division number, viability or cell size + SEM of five biological replicates from two independent experiments.

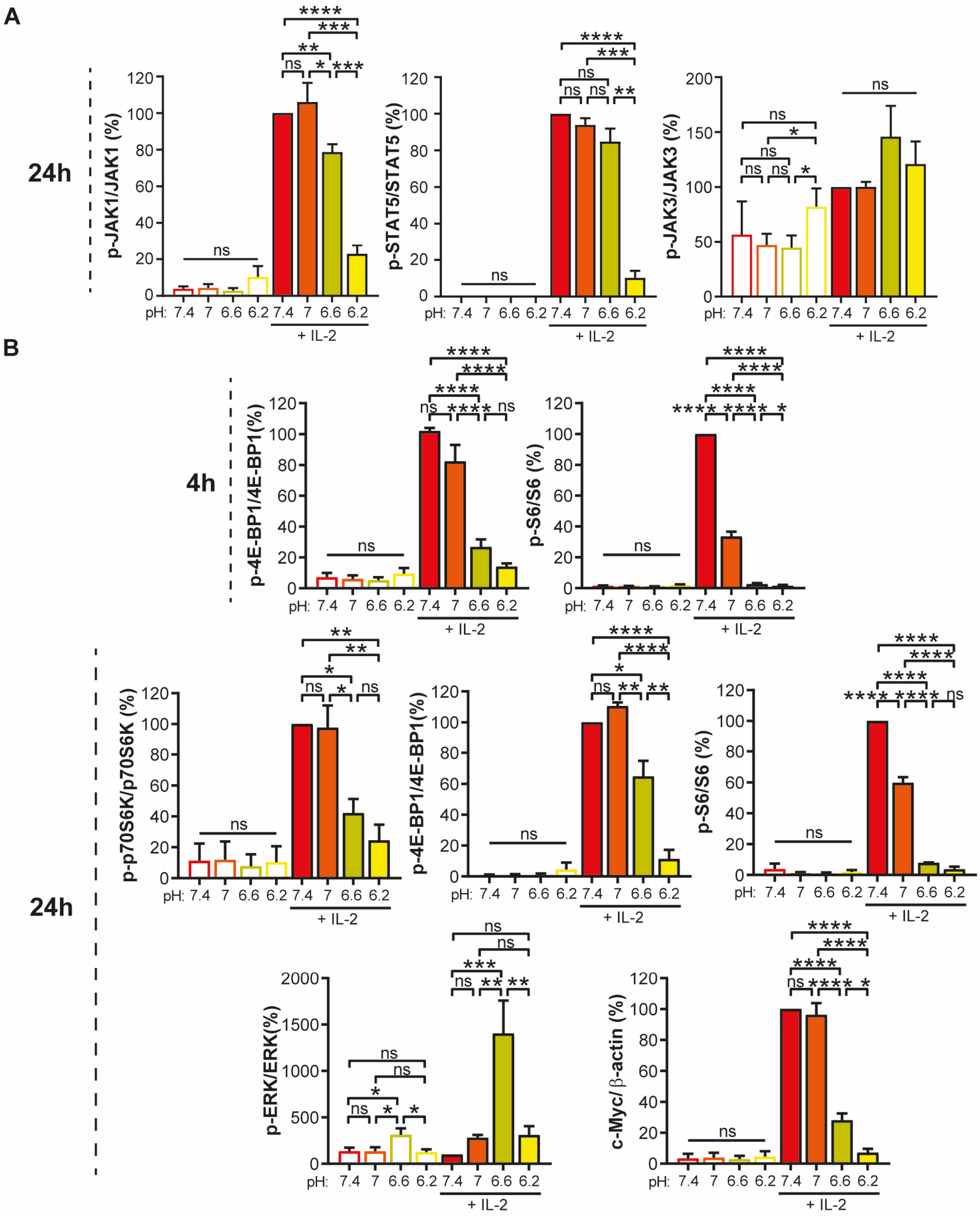

Appendix Figure S2. Acidity lowers IL-2R signaling in CTLs.

(A) IL-2R signaling is disrupted at lower pH. OT-I CTLs were cultured for 24 hours (without prior starving). Bar graphs show the mean levels normalized to the condition "pH7.4 + IL-2" +SEM of at least five biological replicates from at least two independent experiments. ns: not significant, *p<0.05, **p<0.01, ***p<0.001, ****p<0.0001 (one-way repeated measures ANOVA, Tukey post-hoc test).

(B) Low pH disturbs IL-2 –induced mTORC1 pathway and c-Myc levels OT-I CTLs were cultured for 4 or 24 hours (without prior starving). Bar graphs show the mean levels normalized to the condition "pH7.4 + IL-2" +SEM of at least five biological replicates from at least two independent experiments. ns: not

significant, *p<0.05, **p<0.01, ***p<0.001, ****p<0.0001 (one-way repeated measures ANOVA, Tukey post-hoc test).

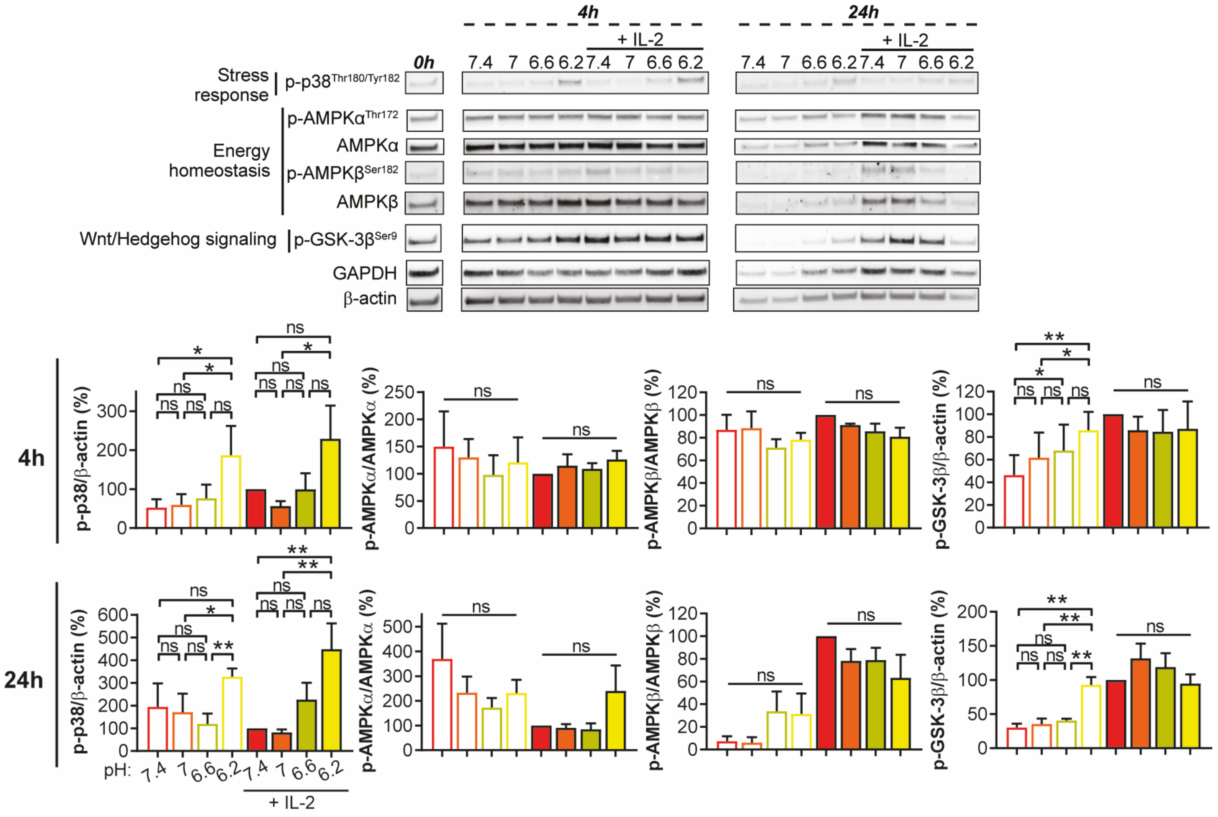

Appendix Figure S3. Impact of pH on pathways that may act on mTORC1 and c-Myc.

OT-I CTLs were cultured at various pH in the presence, or the absence of exogenous murine IL-2 for 4 or 24 hours. One representative western blot coming from the same membranes as the one displayed in ***Fig. EV2C*** is shown. Bar graphs show the mean levels normalized to the condition “pH7.4 + IL-2” ± SEM of at least three biological replicates from at least two independent experiments. ns: not significant, *p<0.05, **p<0.01 (one-way repeated measures ANOVA, Tukey post-hoc test).

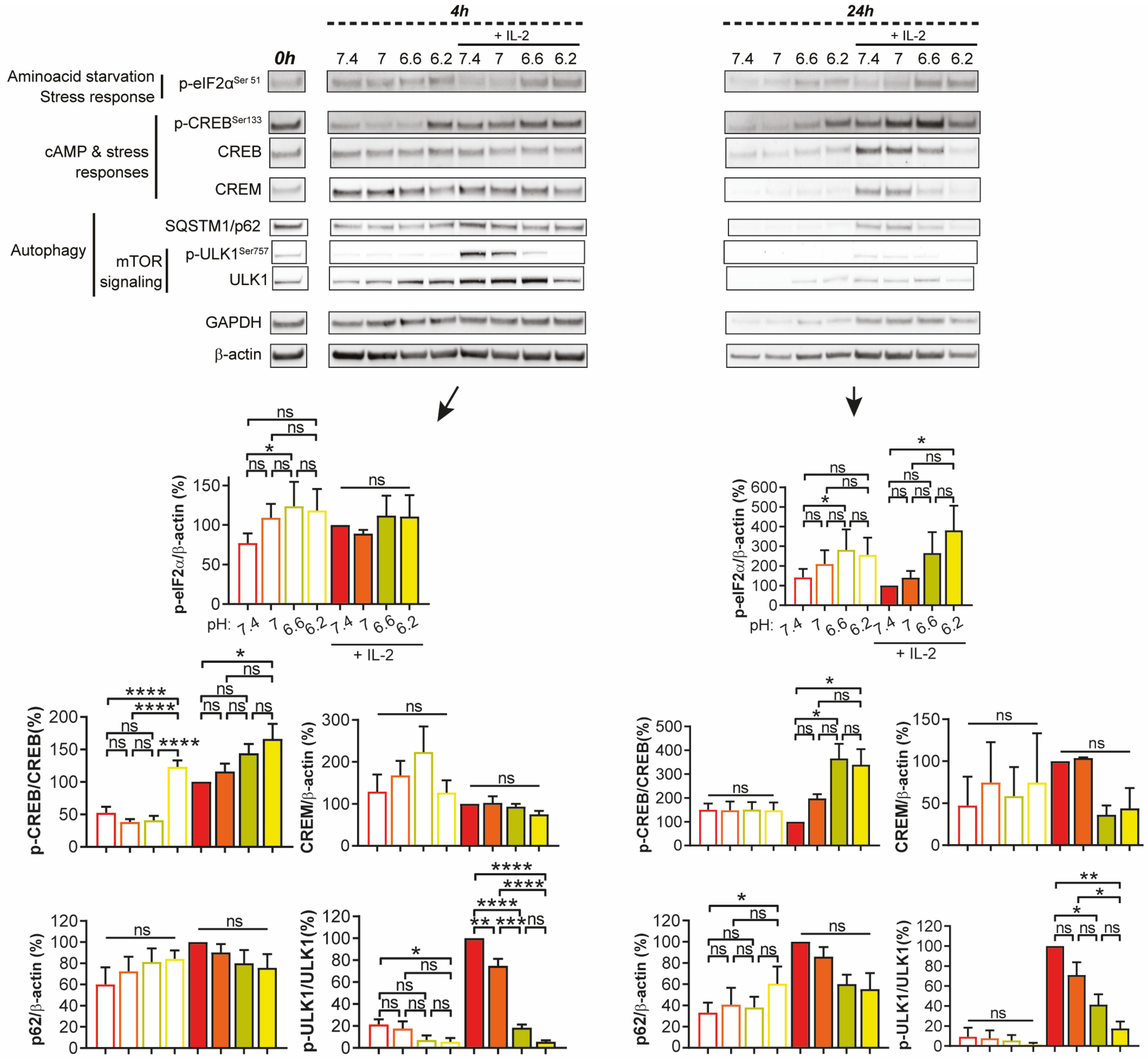

Appendix Figure S4. Impact of pH on pathways that may act on mTORC1 and c-Myc.

OT-I CTLs were cultured at various pH in the presence, or the absence of exogenous murine IL-2 for 4 or 24 hours. One representative western blot is shown. Bar graphs show the mean levels normalized to the condition “pH7.4 + IL-2” ± SEM of at least three biological replicates from at least two independent experiments. ns: not significant, *p<0.05, **p<0.01, ***p<0.001, ****p<0.0001 (one-way repeated measures ANOVA, Tukey post-hoc test). (one-way repeated measures ANOVA, Tukey post-hoc test).

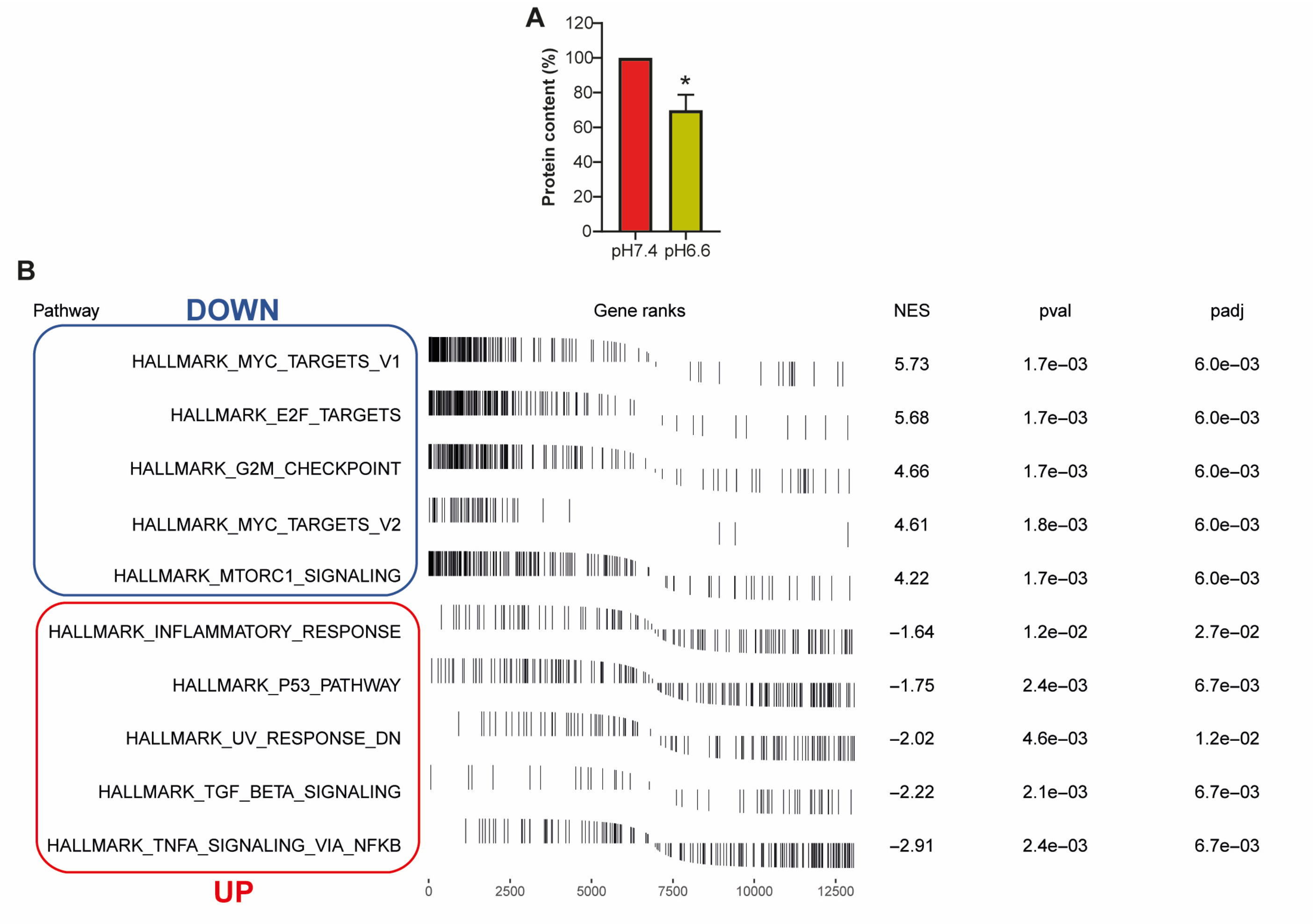

Appendix Figure S5. mTORC1 and c-Myc pathways are lowered at low pH.

(**A**) Acidity limits protein content. OT-I CTLs were cultured for four hours in the presence of exogenous IL-2. Bar graph shows mean protein content, normalized to the condition “pH7.4”, + SEM of four biological replicates from two independent experiments. *p<0.05 (Student’s t-test).

(**B**) Acidity blunts c-Myc targets expression. OT-I CTLs were cultured for twenty-four hours in the presence of exogenous IL-2 at pH7.4 or pH6.6. Results displays GSEA from RNA-seq data from five biological experiments from two independent experiments.

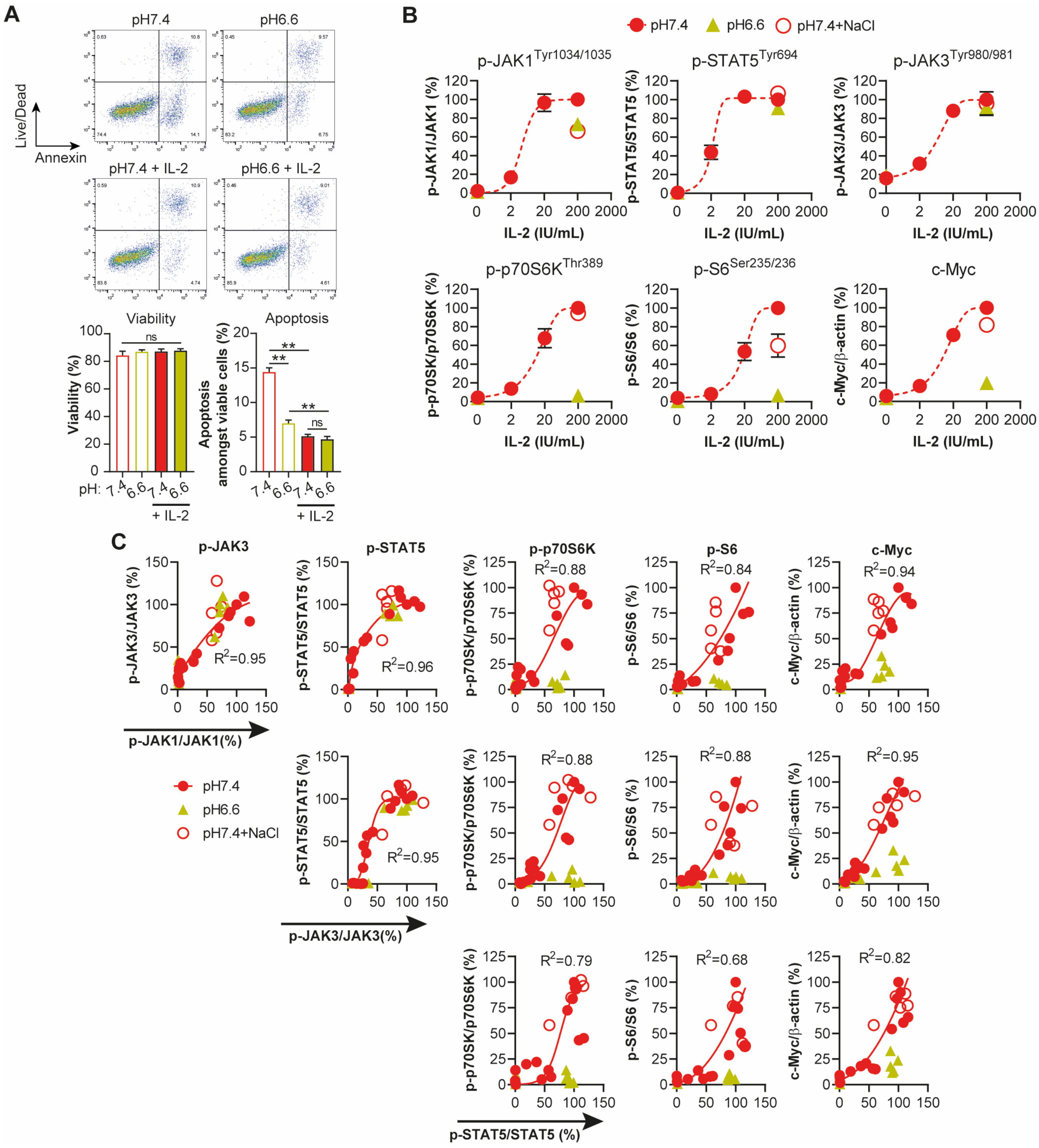

Appendix Figure S6. The impact of acidity on mTORC1 and c-Myc does not seem to be linked to a disturbance in the activation of upstream IL-2R signaling transducers in polyclonal CTLs.

(**A**) Acidity does not increase apoptosis or cell death of CTLs upon a four-hour treatment. OT-I CTLs were cultured for 4 hours. Representative dot plots show Annexin and Live/Dead staining obtained by flow cytometry. Bar graph shows percent cell viability (Live/Dead negative fraction) or apoptosis amongst viable cells (Annexin positive amongst Live/Dead negative fraction) + SEM of four biological replicates from two independent experiments.

(**B**) Dose-dependent activation of the first signaling transducers, mTORC1 pathway and accumulation of c-Myc. C57BL/6 CTLs were cultured for 4 hours. In parallel, CTLs were cultured at pH7.4 with 31mM NaCl. Results show the mean levels normalized to the condition “pH7.4 + 200 IU/mL IL-2” ± SEM of at least four biological replicates from two independent experiments.

(**C**) Correlation between the activation of first signaling transducers and mTORC1 pathway targets activation, or c-Myc levels. C57BL/6 CTLs were cultured for 4 hours. Results show individual values obtained from (**A**). Correlation curves, and the associated R^2, were calculated for the condition “pH7.4” (red solid lines).

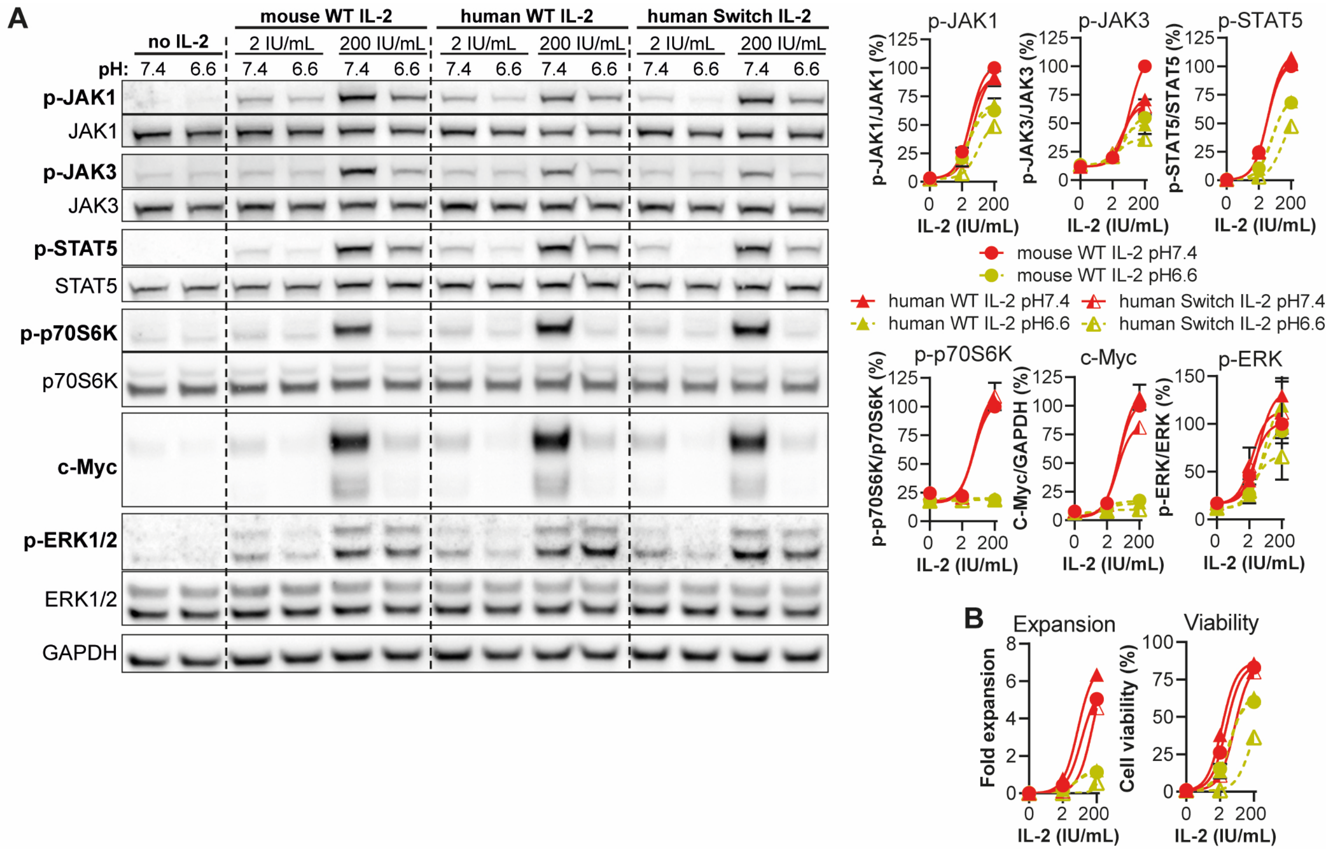

Appendix Figure S7. The Switch IL-2 mutant does not improve mTORC1 signaling, c-Myc levels or T-cell expansion at low pH of murine CD8+ T cells.

(**A**) Acidity, Impact of the IL-2 "Switch" cytokine on IL-2R signaling. OT-I CTLs were cultured for four hours. Blots of one representative experiment are shown. Line graphs shows the mean ± SEM of three biological replicates from two independent experiments.

(**B**) Impact of the IL-2 "Switch" cytokine on expansion and viability. OT-I CTLs were cultured for three days. Dot plots shows individual values of three biological replicates from two independent experiments. A correlation curve is displayed per biological replicate.

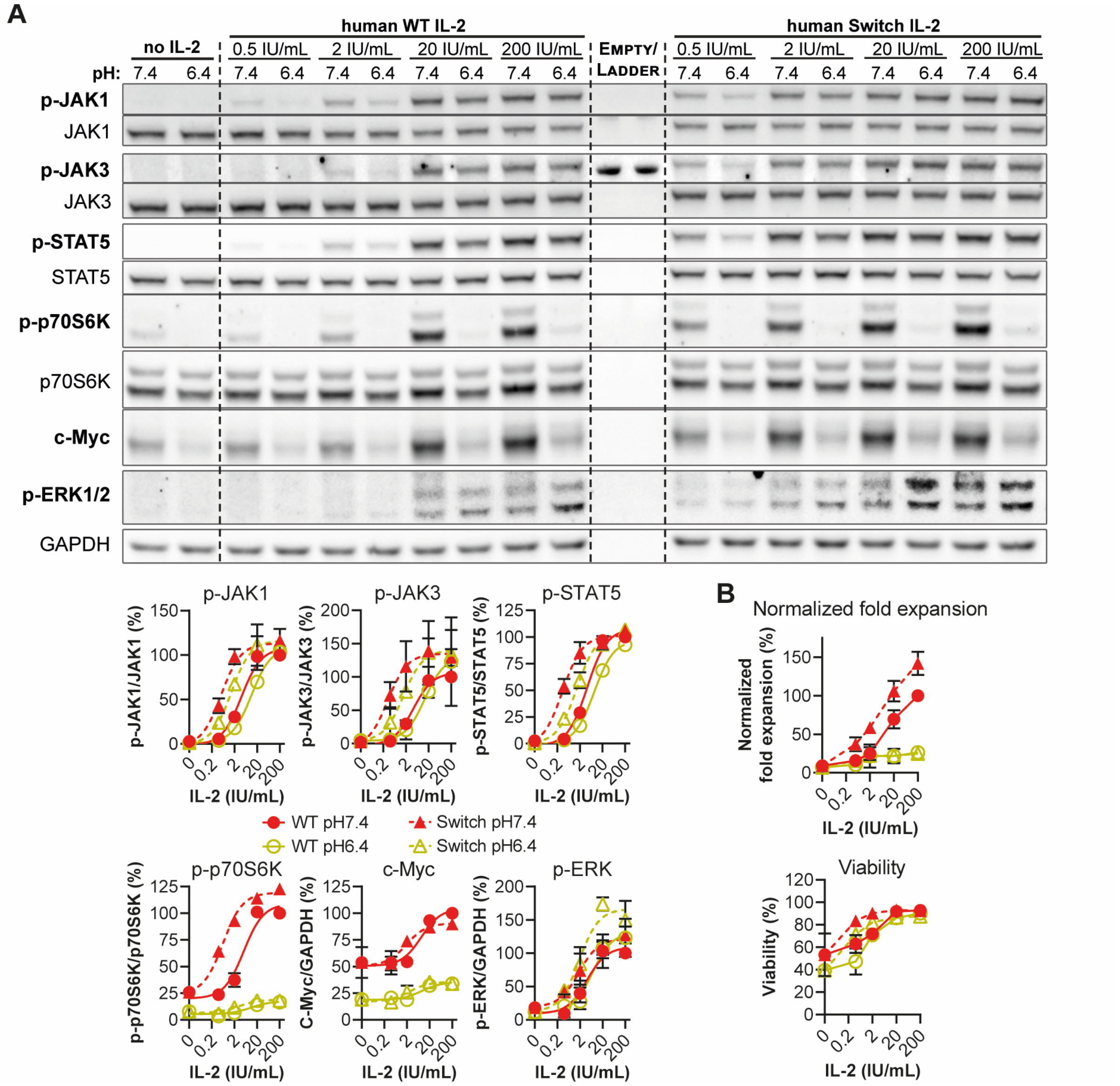

Appendix Figure S8. The Switch IL-2 mutant does not improve mTORC1 signaling, c-Myc levels or T-cell expansion at low pH of human CD8⁺ T cells.

(**A**) Impact of the IL-2 “Switch” cytokine on IL-2R signaling in human $CD8^+$ T cells. Human $CD8^+$ T cells were cultured for four hours. Blots of one representative experiment are shown. Line graphs shows the mean ± SD of two biological replicates.

(**B**) Impact of the IL-2 “Switch” cytokine on expansion and viability of human $CD8^+$ T cells. Human $CD8^+$ T cells were cultured for five days. Line graphs shows the mean ± SD of two biological replicates.

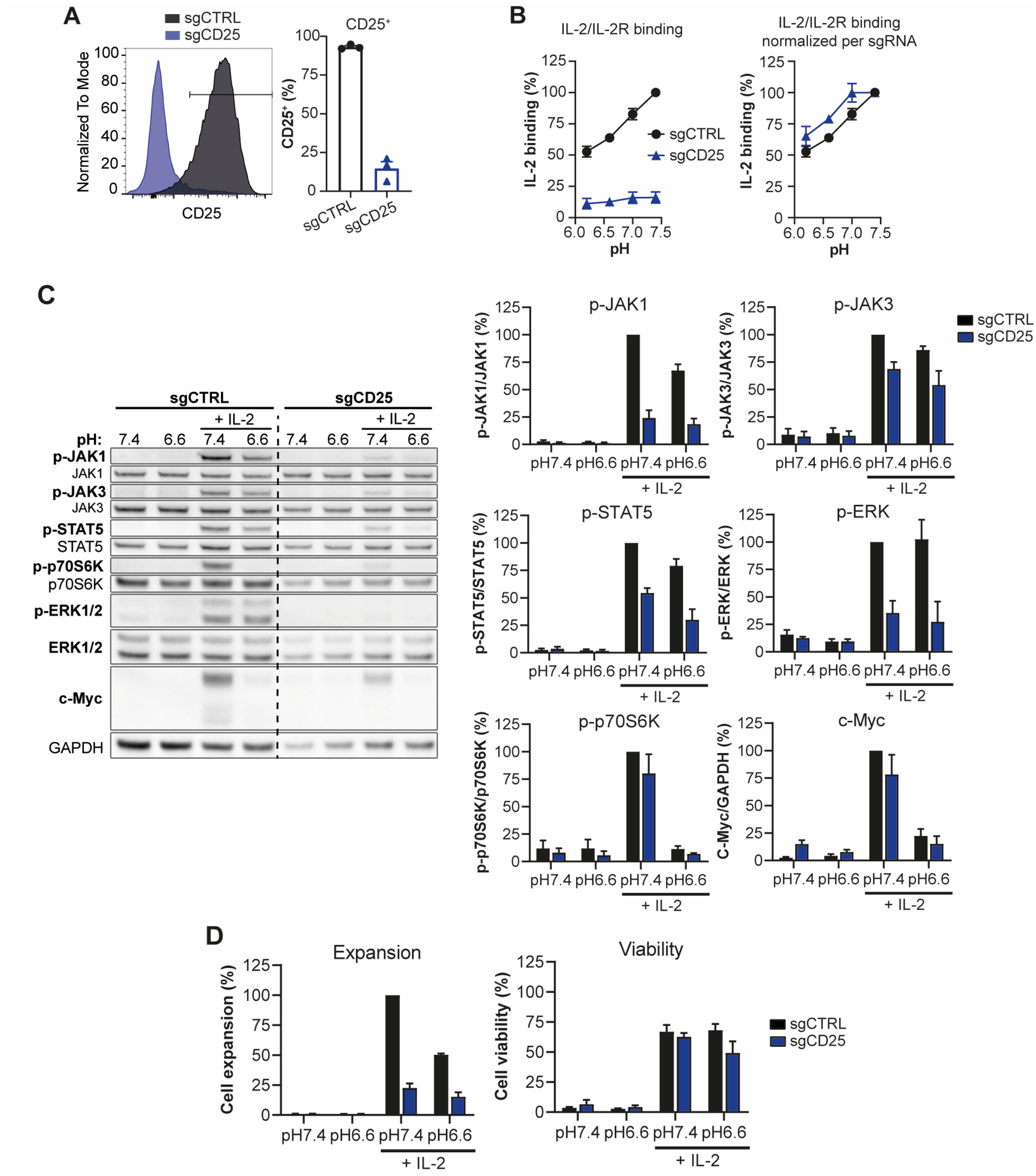

Appendix Figure S9. Knocking out CD25 reveals that it is not involved in the pattern observed at low pH.

(**A**) CD25 expression levels upon knock out using CRISPR/Cas9. OT-I x CRISPR/Cas9 CTLs were transduced with retroviruses expressing a negative control sgRNA or a CD25 sgRNA. Upon expansion and Thy1.1 enrichment, CD25 expression was analyzed by flow cytometry. One representative histogram is shown. Bar graph shows the percentage of CD25⁺ CTLs ± SEM of three biological replicates out of two independent experiments.

(**B**) Impact of pH and CD25 on IL-2/IL-2R binding. IL-2/IL-2R binding was determined by incubating cells used in (**A**) with biotinylated IL-2 at various pH. Line graph on the left shows IL-2/IL-2R binding

normalized to the condition sgCTRL pH7.4 ± SEM of three biological replicates out of two independent experiments. Line graph on the right displays the same results normalized to the respective sgRNA at pH7.4.

(**C**) Impact of pH and CD25 on IL-2R signaling. The same cells as used in (**A**,**B**) were cultured for four hours. Bar graphs shows the mean + SEM of three biological replicates from two independent experiments.

(**D**) Impact of the IL-2 “Switch” cytokine on expansion and viability. The same cells as used in (**A**,**B**) were cultured for four days. Bar graphs shows the mean + SEM of three biological replicates from two independent experiments.

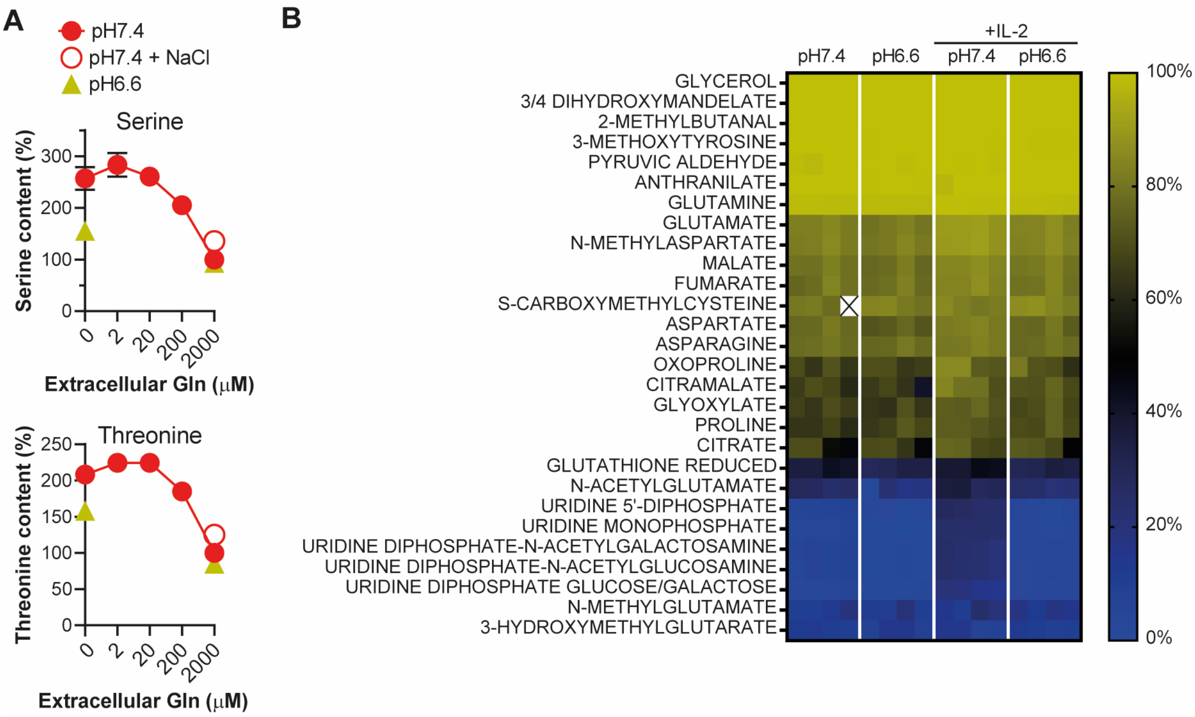

Appendix Figure S10. Glutamine metabolism exploration at low pH.

(**A**) Dose-dependent impact of exogenous glutamine on intracellular serine and threonine levels. OT-I CTLs were cultured for 4 hours with exogenous murine IL-2. Results show the mean intracellular amino acid content normalized to the condition “pH7.4 + 2000μM Gln” ± SEM of four biological replicates out of two independent experiments.

(**B**) Metabolites incorporating extracellular glutamine. OT-I CTLs were cultured for four hours with isotopic ^{13}C-glutamine (2mM). Heat map shows the proportion of the indicated intracellular amino acid that incorporated ^{13}C using a color code, as outlined on the ladder. Each condition has four biological replicates from two independent experiments. Empty square with a cross inside means the metabolite was not detected. Metabolites are ordered based on their mean at pH7.4 in the presence of IL-2, and those with less than 10% of ^{13}C incorporation are not shown.

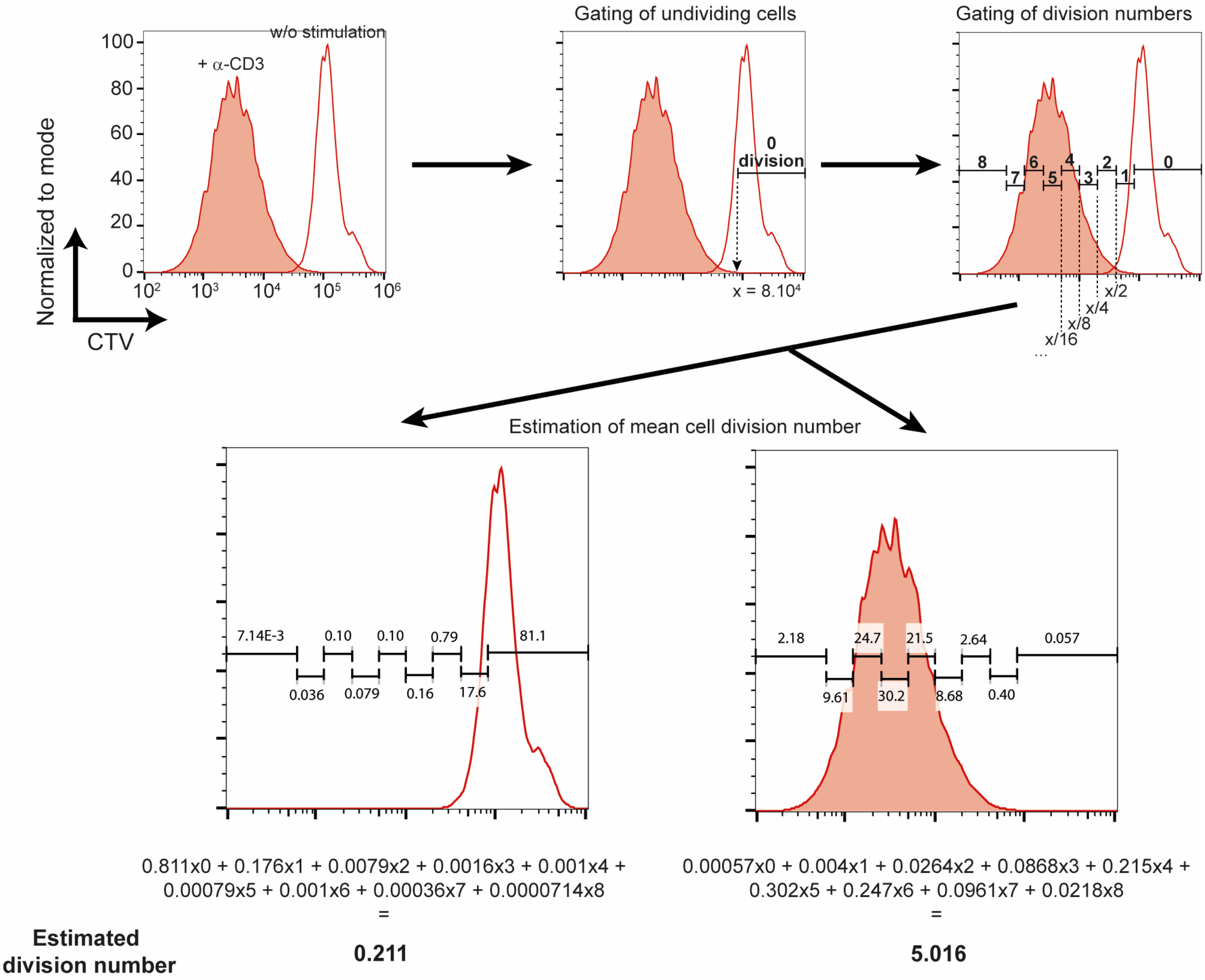

Appendix Figure S11. Methodology to estimate mean cell division number.

CTV-labelled CTLs were cultured for 3 days in the presence or absence of anti-CD3. Unstimulated cells allow to create the “0 division” gate. The lowest value of this gate is then halved iteratively to create the other gates representing 1, 2, 3, 4, 5, 6, 7 and 8 cell divisions. Finally, estimated mean cell division number is obtained by summing up each percentage of cells belonging to a particular gate multiplied to the corresponding cell division number.
