# Supplementary figures and images for: Acidity suppresses CD8 + T-cell function by perturbing IL-2, mTORC1, and c-Myc signaling
Source: EMBO J. 2024 Sep 16;43(21):8. doi: 10.1038/s44318-024-00235-w (PMC11535206; doi:10.1038/s44318-024-00235-w)

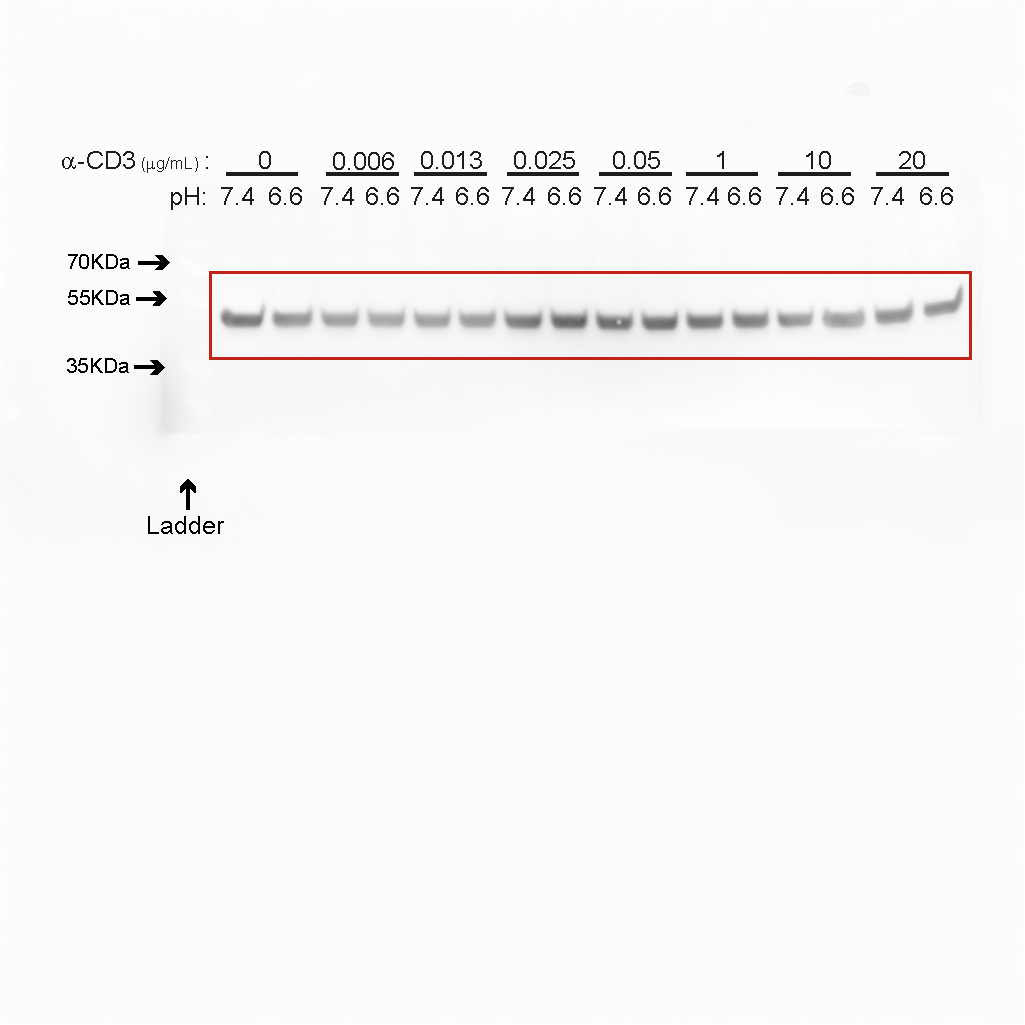

Supplement: Supplementary file 6 — Source data Fig. 4 [file 44318_2024_235_MOESM6_ESM.zip › Figure 4/4A/actin-2min.tif]

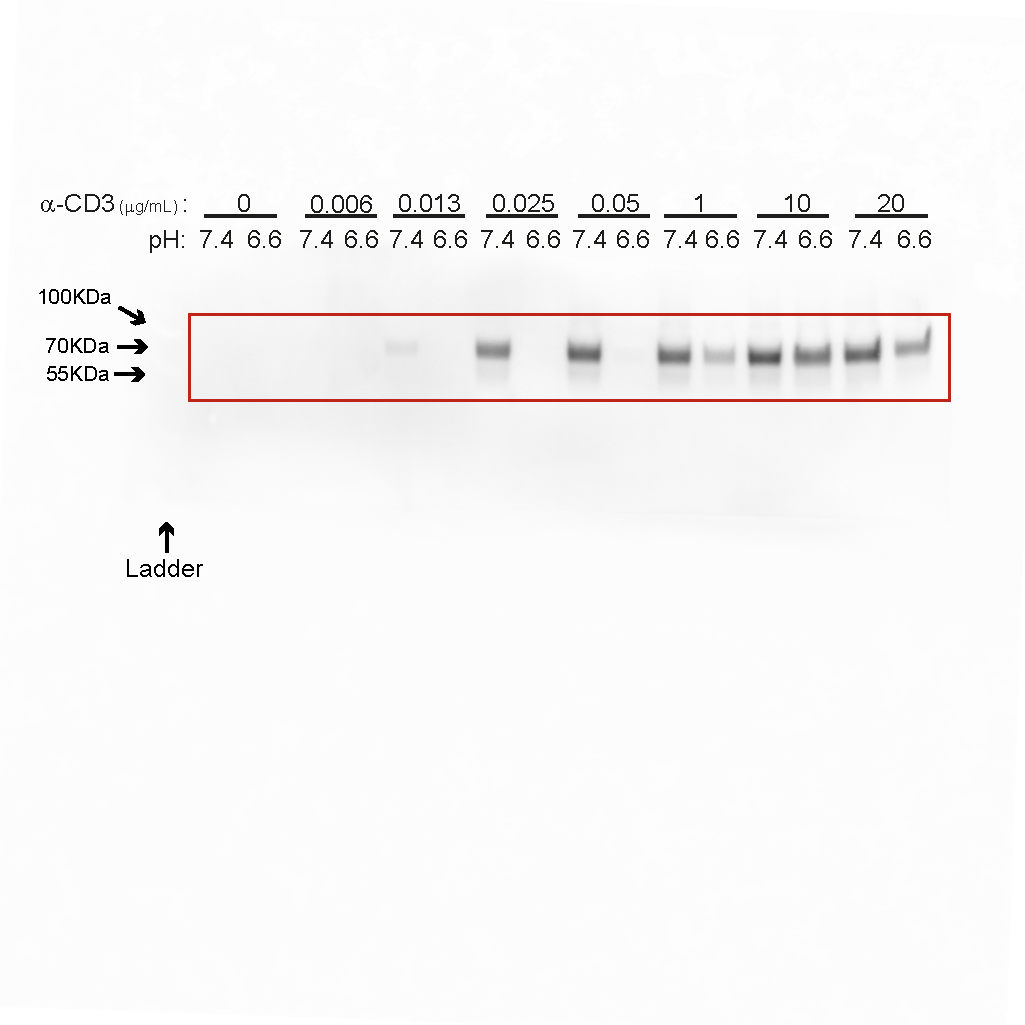

Supplement: Supplementary file 6 — Source data Fig. 4 [file 44318_2024_235_MOESM6_ESM.zip › Figure 4/4A/cmyc-10sec.tif]

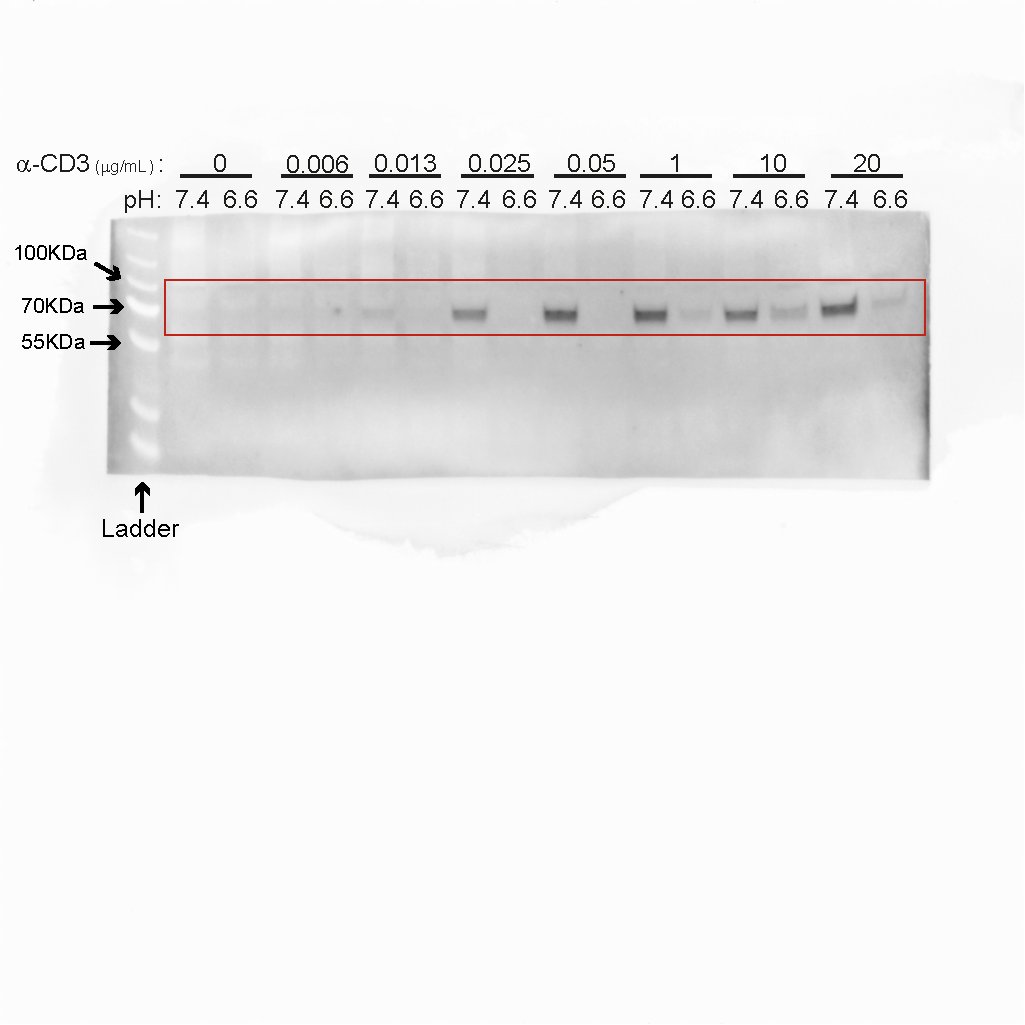

Supplement: Supplementary file 6 — Source data Fig. 4 [file 44318_2024_235_MOESM6_ESM.zip › Figure 4/4A/p-p70S6K-7min30.tif]

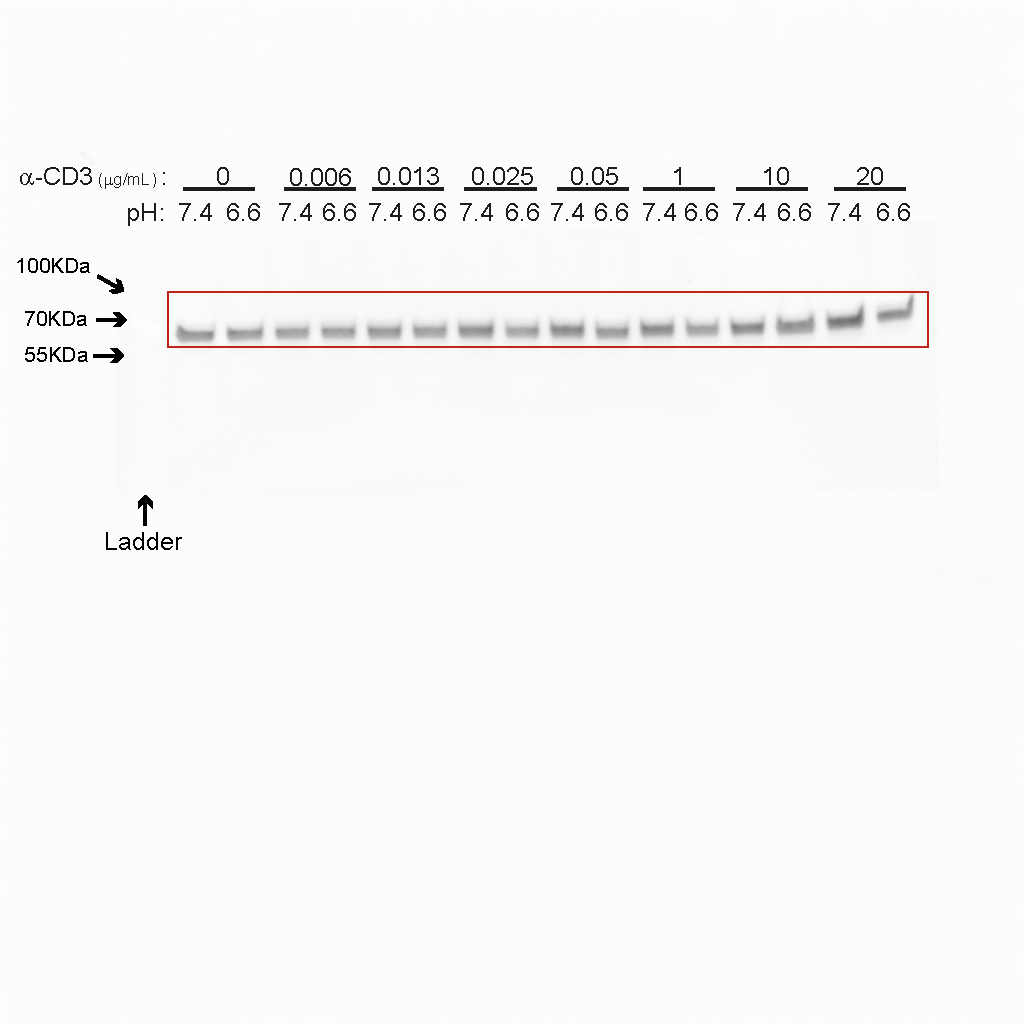

Supplement: Supplementary file 6 — Source data Fig. 4 [file 44318_2024_235_MOESM6_ESM.zip › Figure 4/4A/p70S6K-1min.tif]

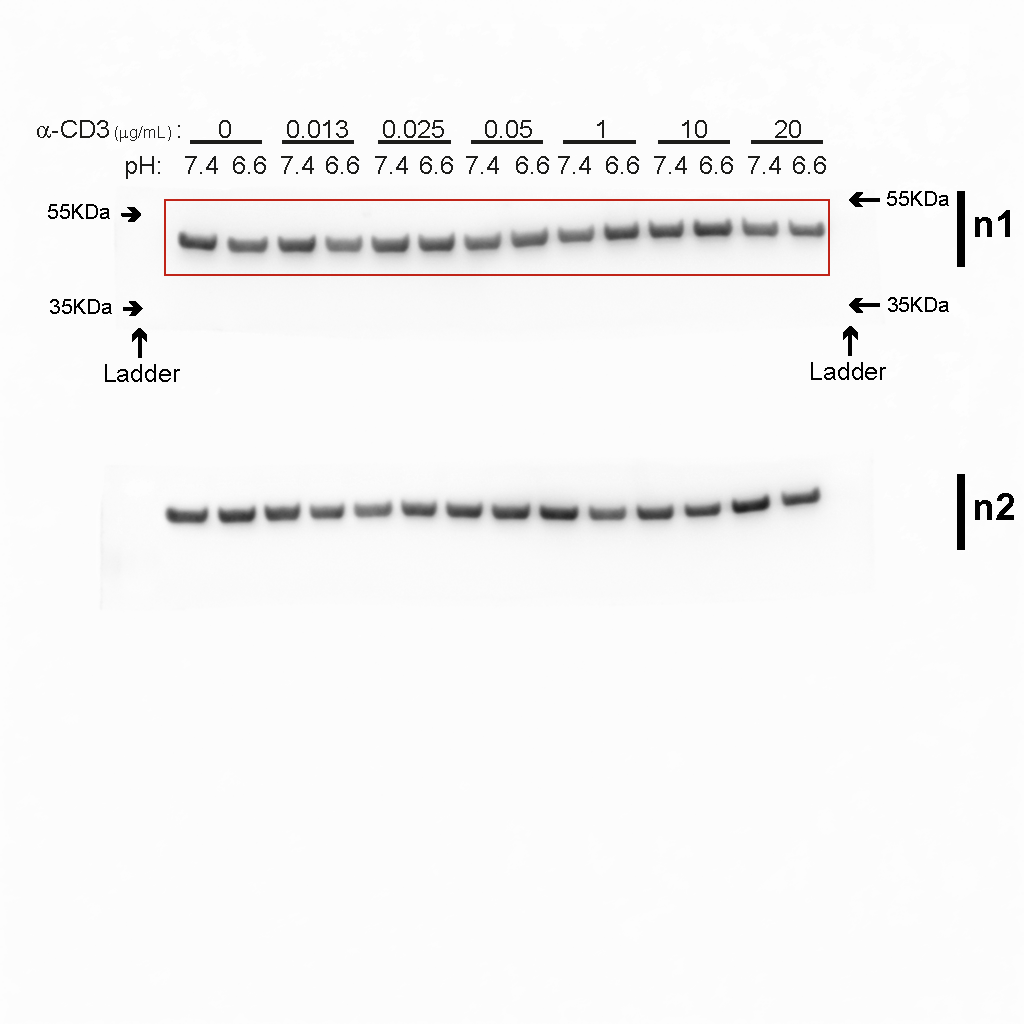

Supplement: Supplementary file 6 — Source data Fig. 4 [file 44318_2024_235_MOESM6_ESM.zip › Figure 4/4B/actin-7s.tif]

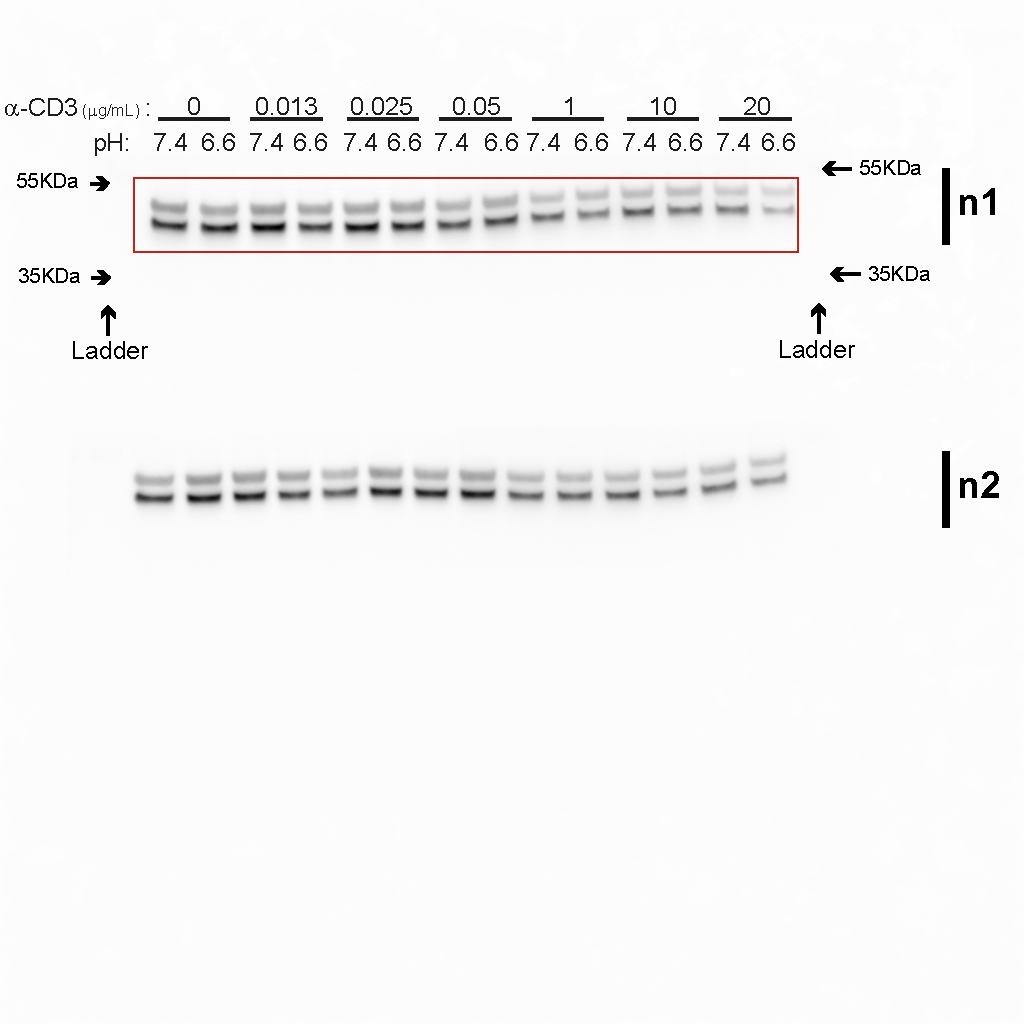

Supplement: Supplementary file 6 — Source data Fig. 4 [file 44318_2024_235_MOESM6_ESM.zip › Figure 4/4B/erk-6s.tif]

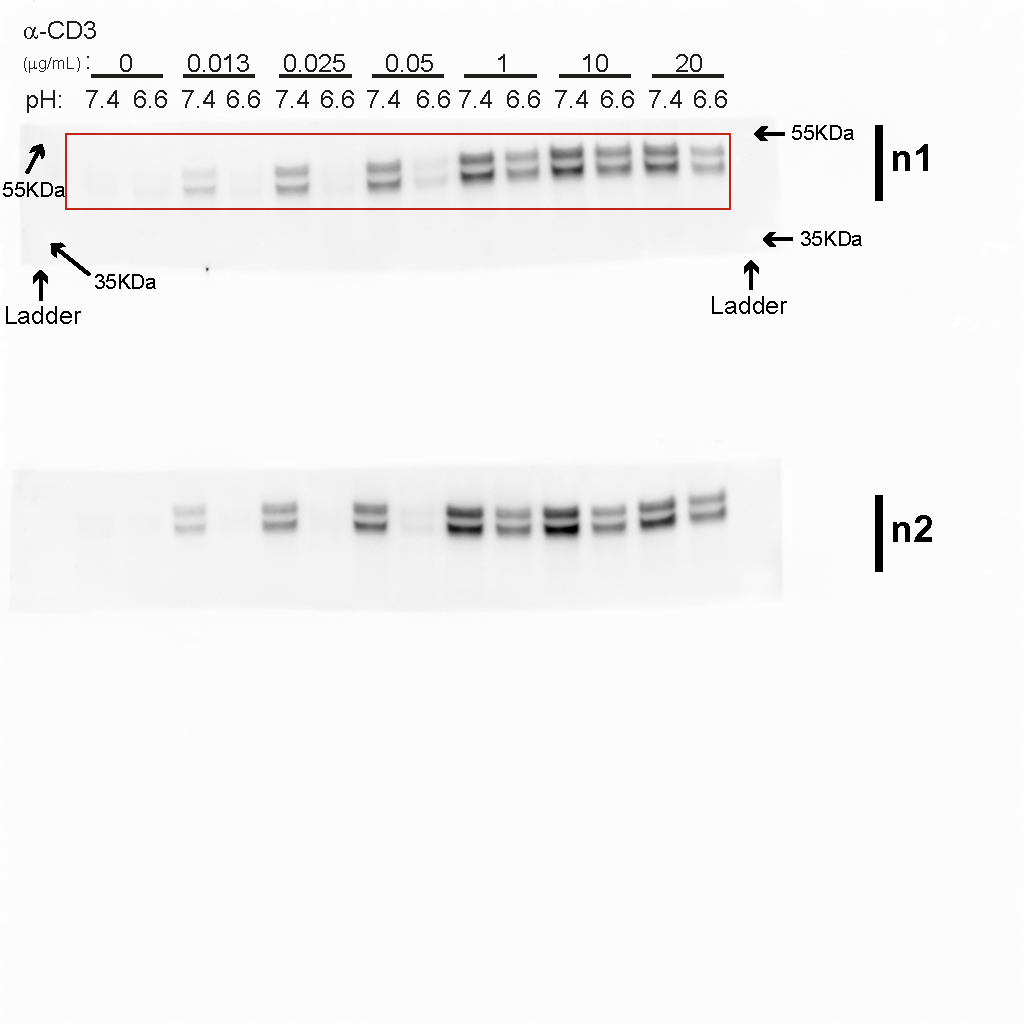

Supplement: Supplementary file 6 — Source data Fig. 4 [file 44318_2024_235_MOESM6_ESM.zip › Figure 4/4B/perk-2min30.tif]

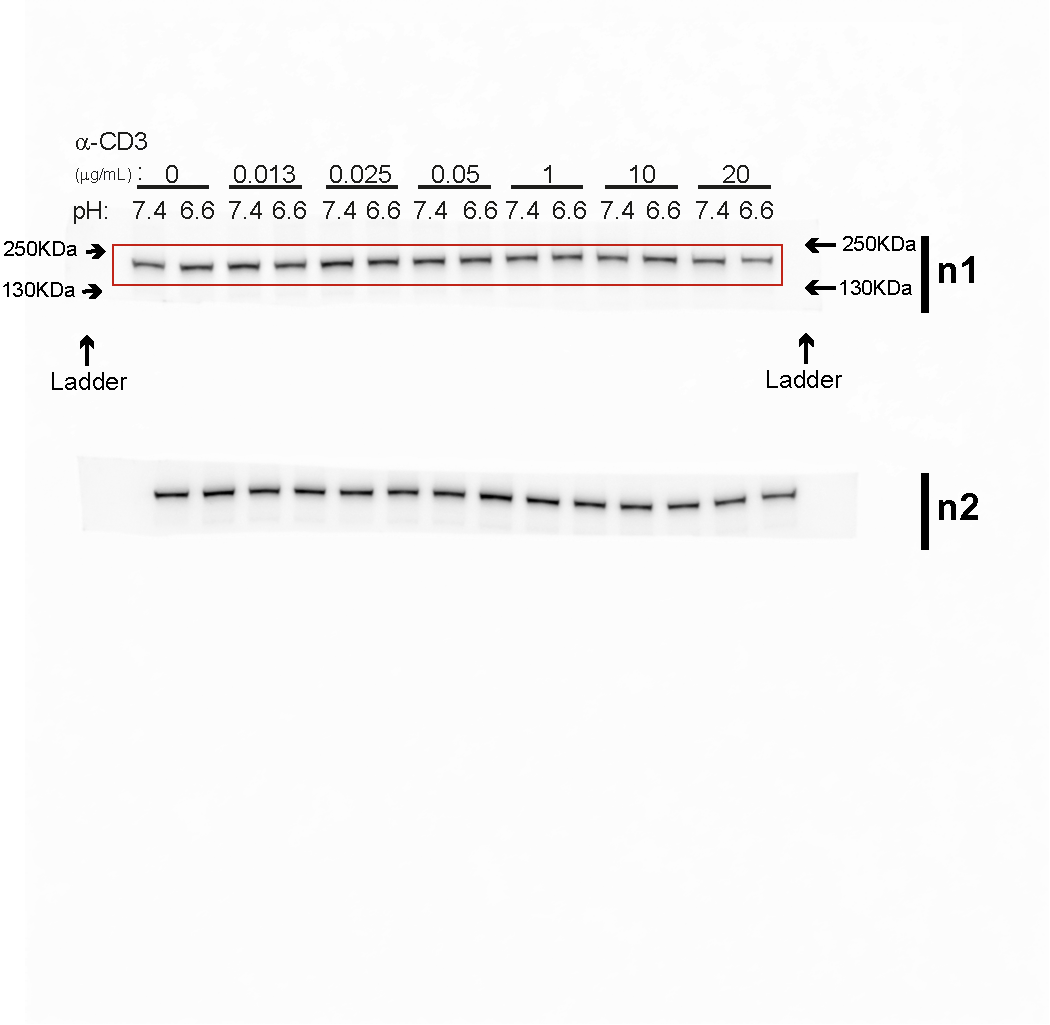

Supplement: Supplementary file 6 — Source data Fig. 4 [file 44318_2024_235_MOESM6_ESM.zip › Figure 4/4B/plcg1-4s.tif]

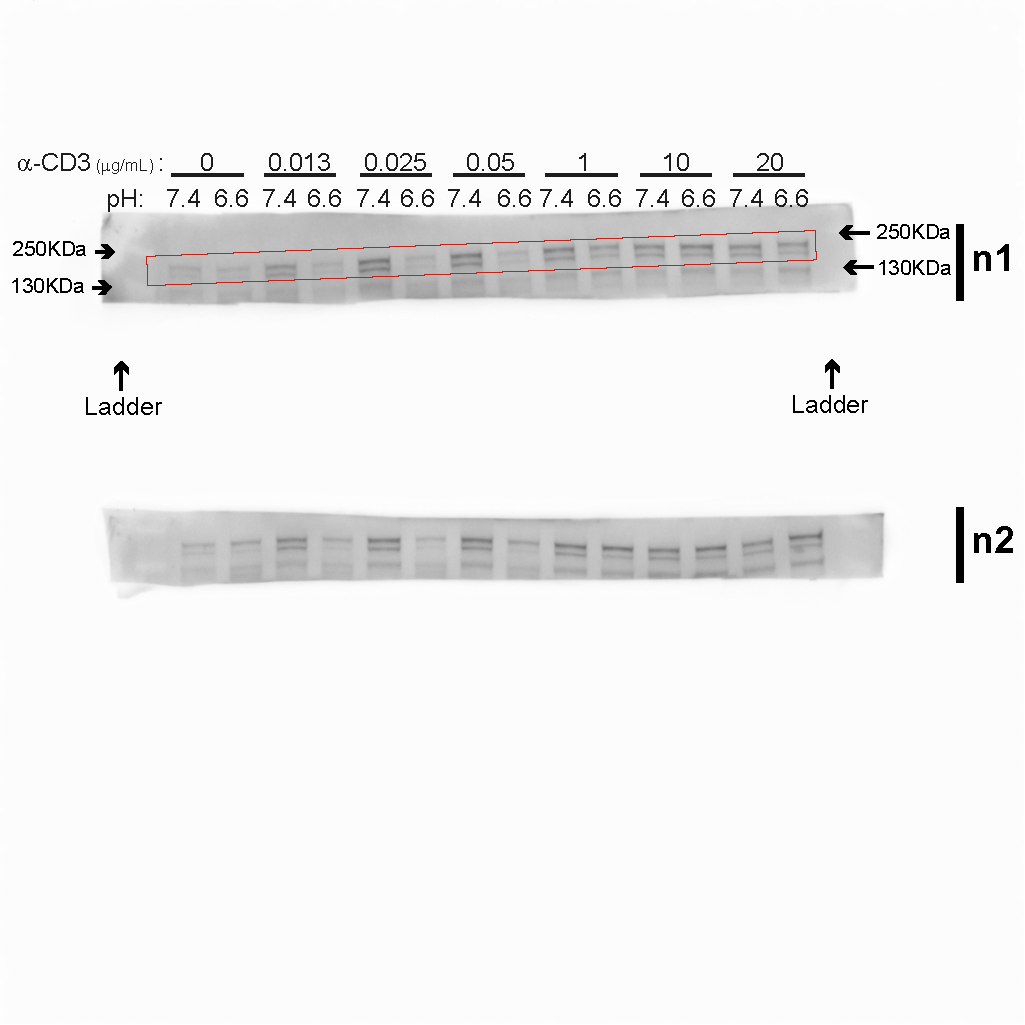

Supplement: Supplementary file 6 — Source data Fig. 4 [file 44318_2024_235_MOESM6_ESM.zip › Figure 4/4B/pplcg-1min30.tif]

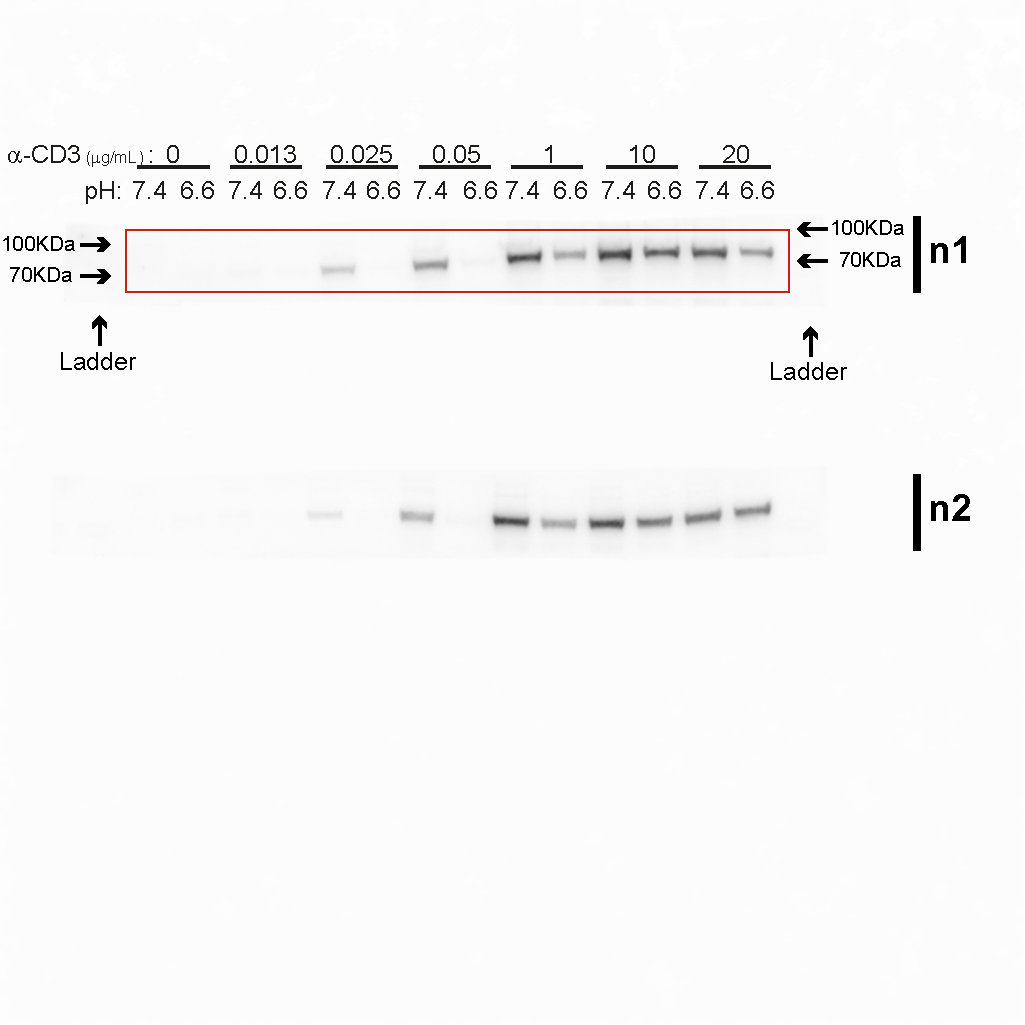

Supplement: Supplementary file 6 — Source data Fig. 4 [file 44318_2024_235_MOESM6_ESM.zip › Figure 4/4B/pslp76-45s.tif]

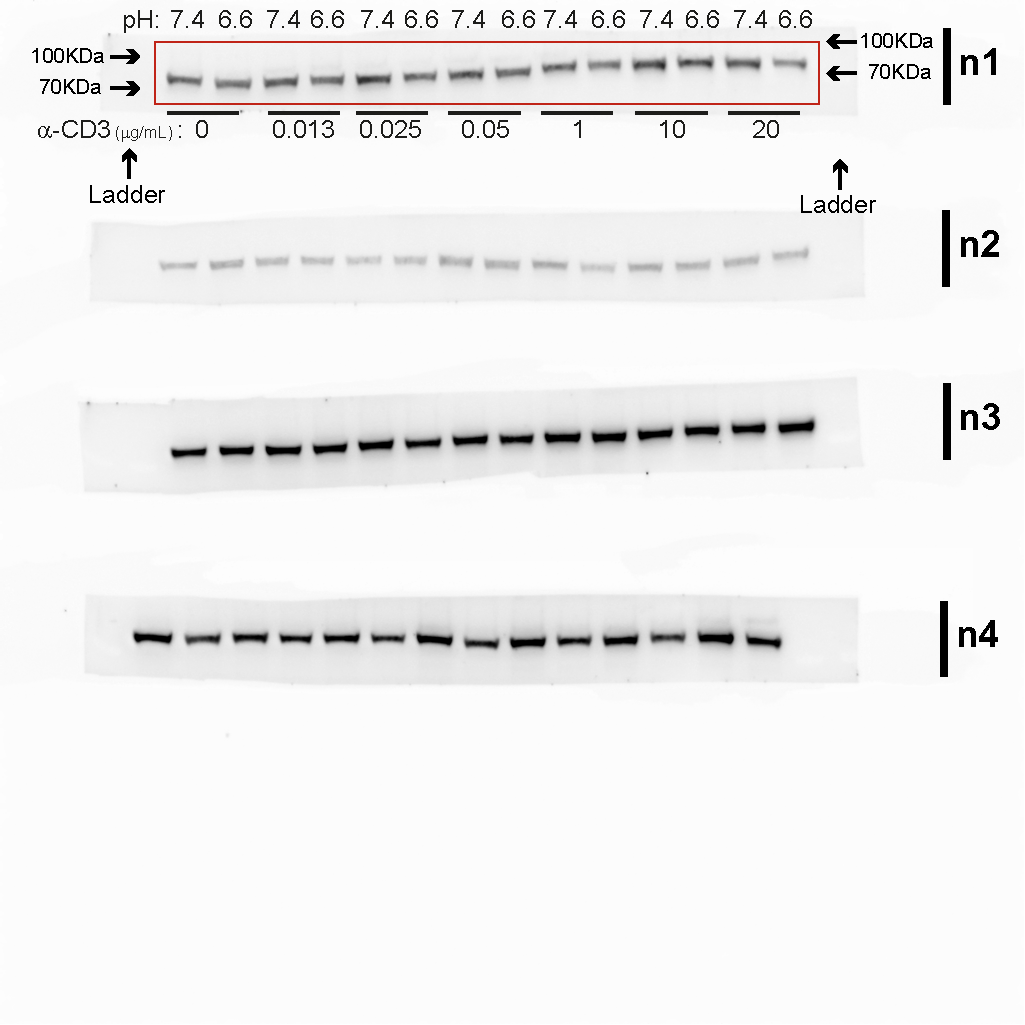

Supplement: Supplementary file 6 — Source data Fig. 4 [file 44318_2024_235_MOESM6_ESM.zip › Figure 4/4B/slp76-1min45s.tif]

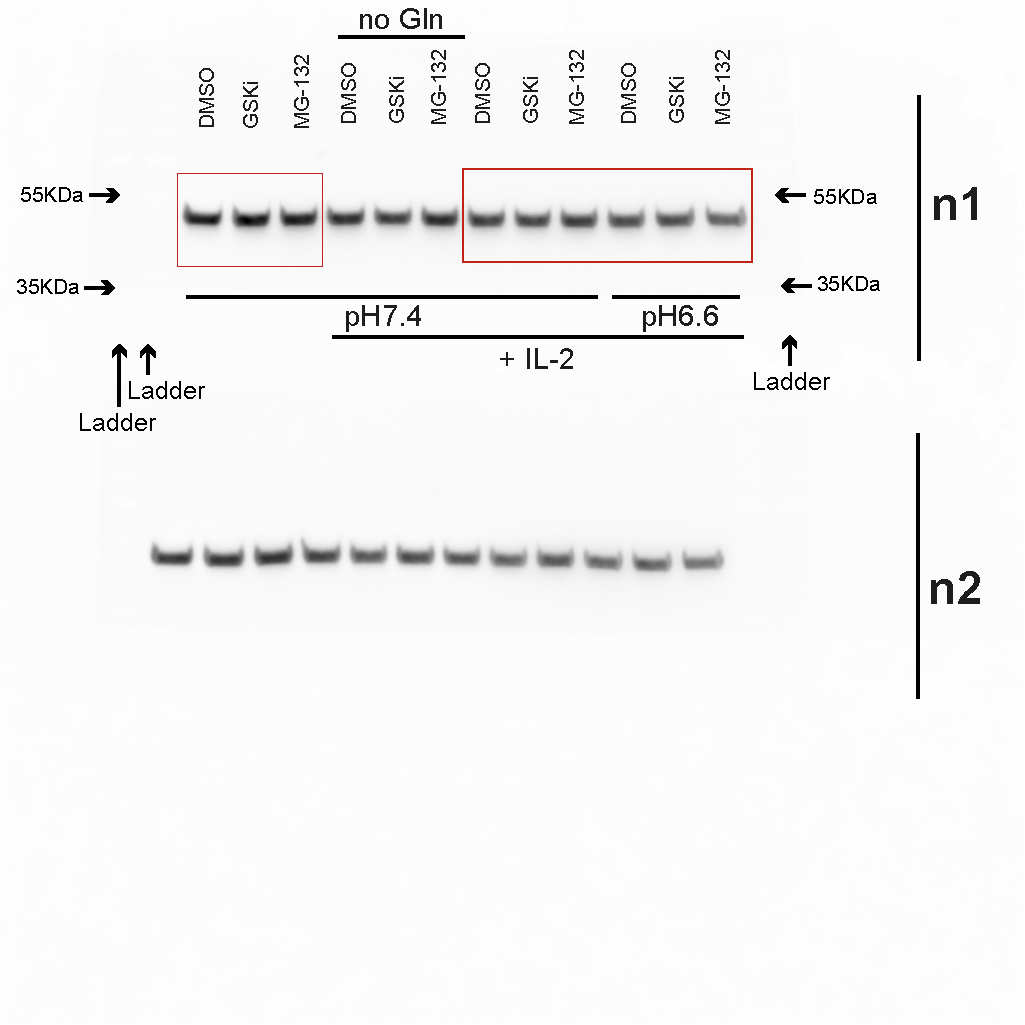

Supplement: Supplementary file 7 — Source data Fig. 5 [file 44318_2024_235_MOESM7_ESM.zip › Figure 5/5F/actin-2s.tif]

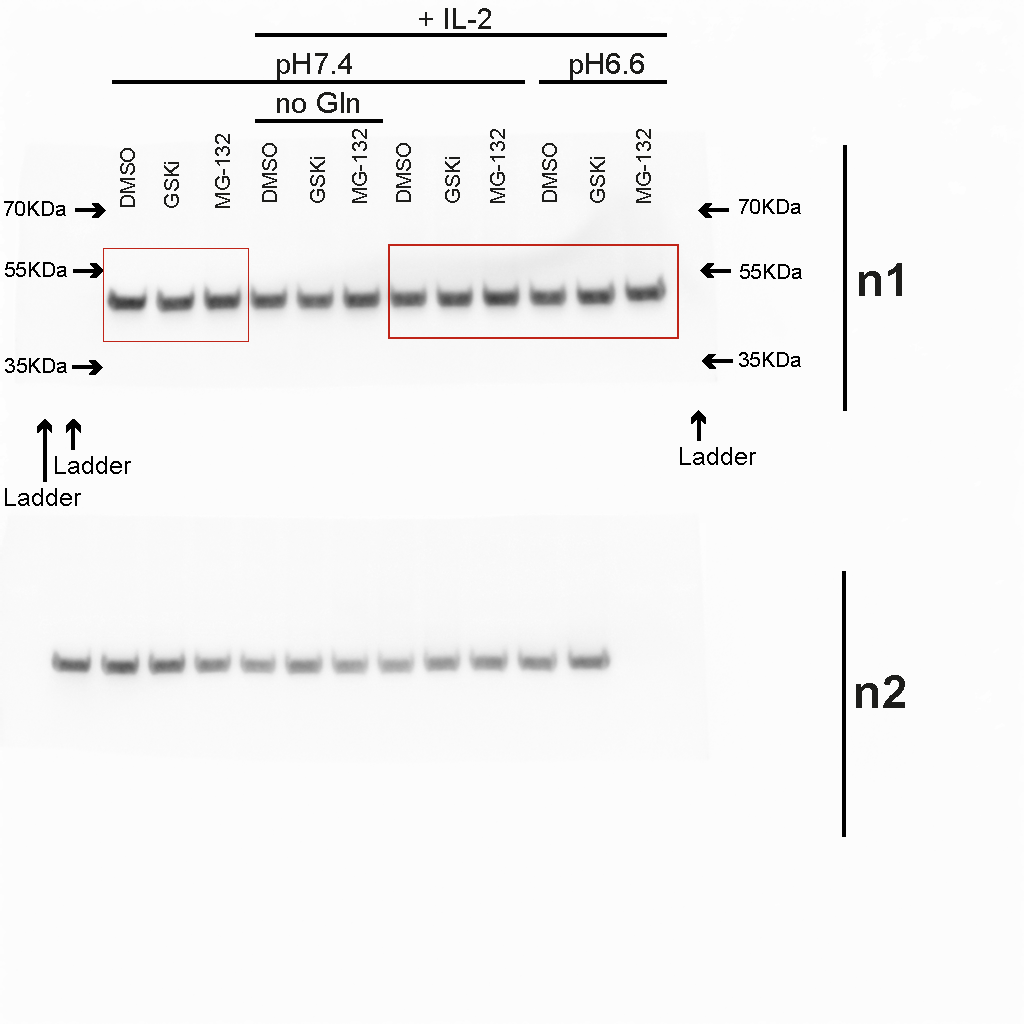

Supplement: Supplementary file 7 — Source data Fig. 5 [file 44318_2024_235_MOESM7_ESM.zip › Figure 5/5F/actin_for_pmyc-3s.tif]

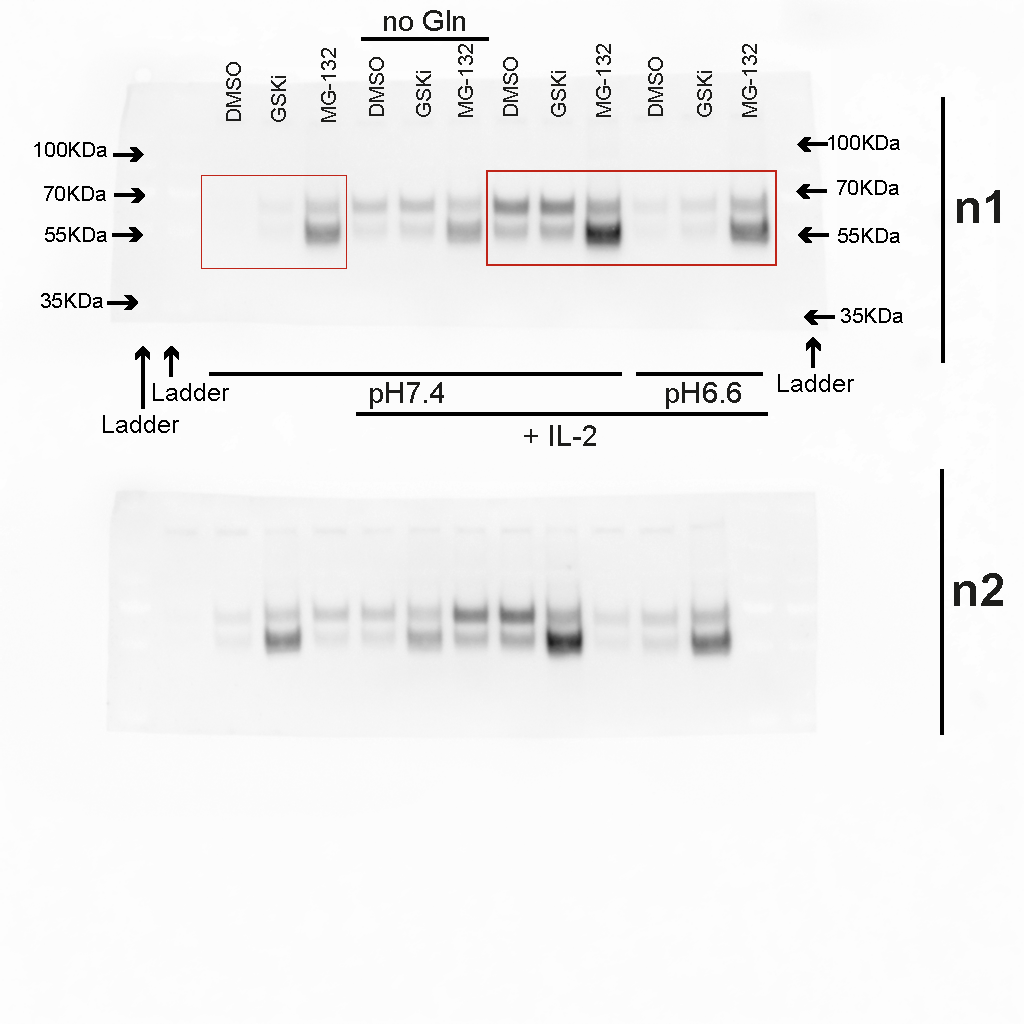

Supplement: Supplementary file 7 — Source data Fig. 5 [file 44318_2024_235_MOESM7_ESM.zip › Figure 5/5F/myc-30s.tif]

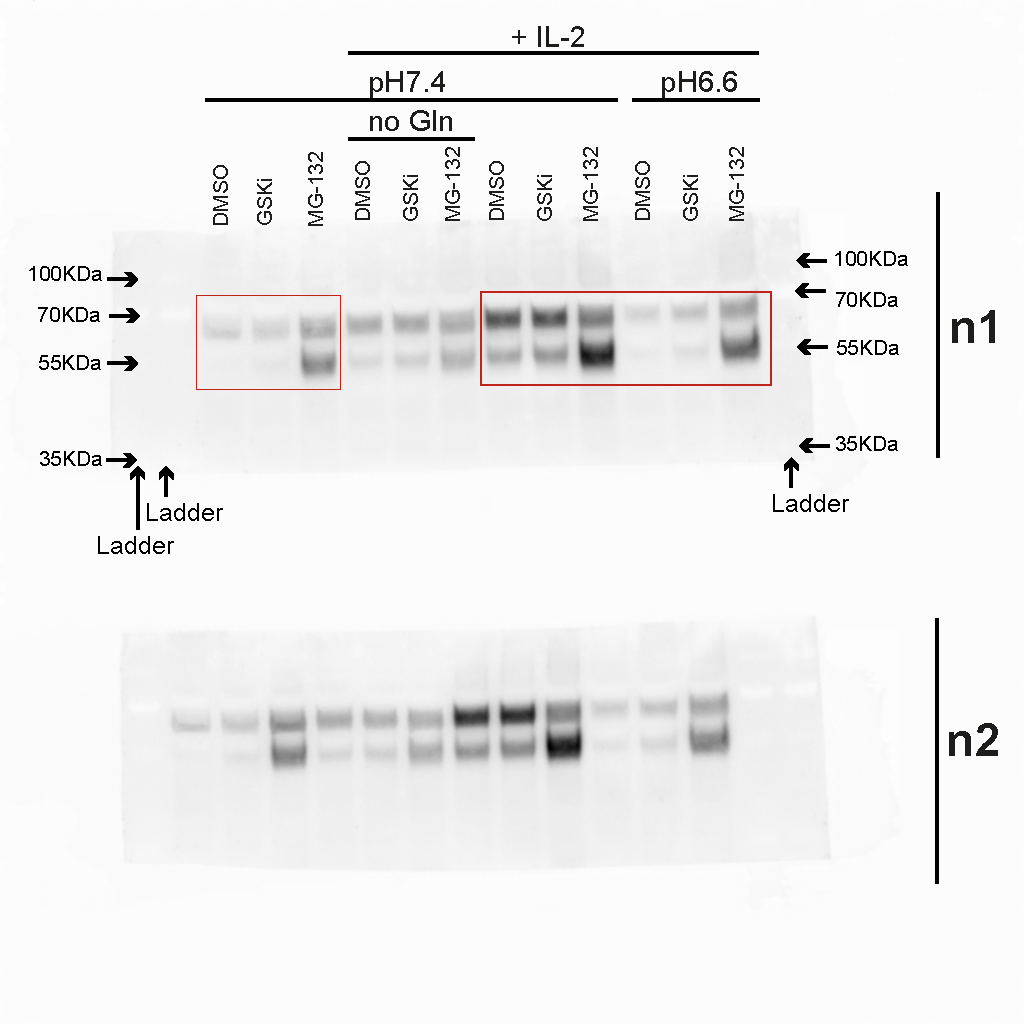

Supplement: Supplementary file 7 — Source data Fig. 5 [file 44318_2024_235_MOESM7_ESM.zip › Figure 5/5F/pcmycSer-2min40.tif]

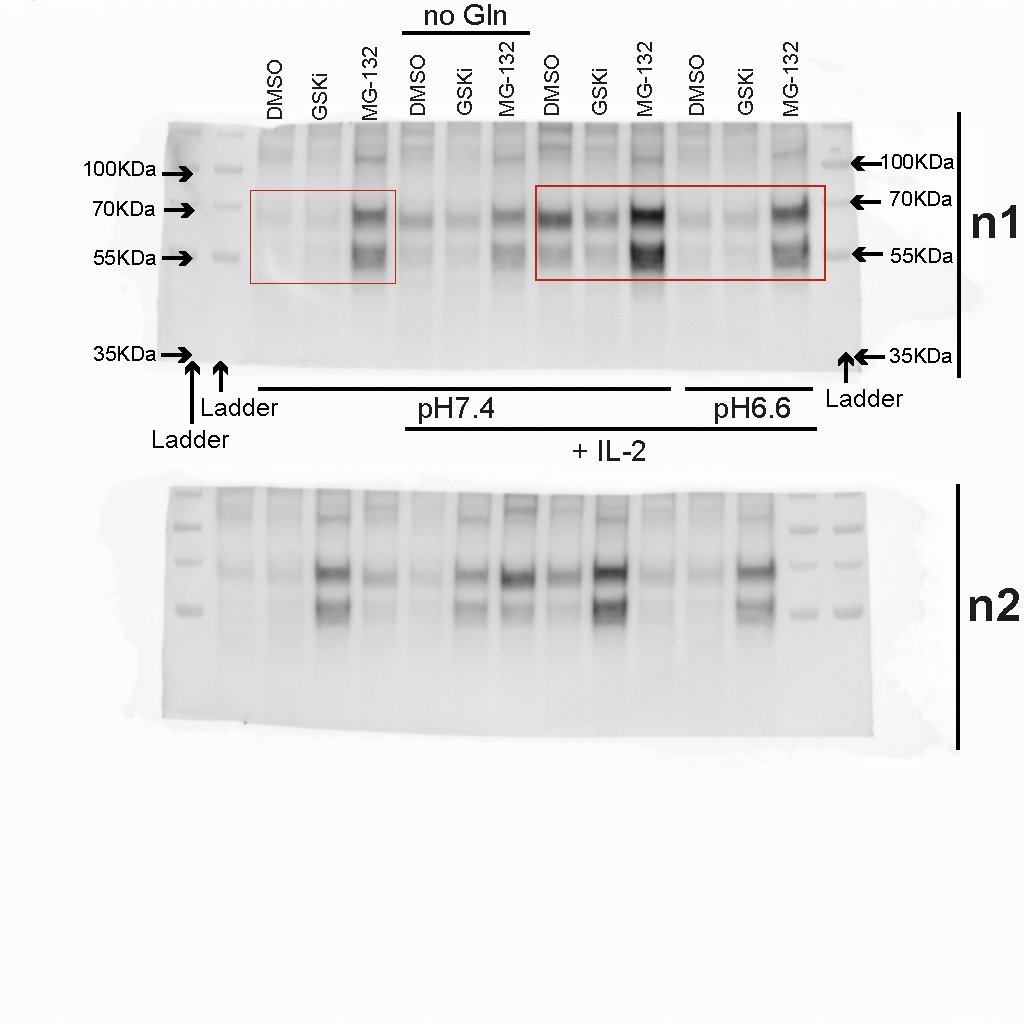

Supplement: Supplementary file 7 — Source data Fig. 5 [file 44318_2024_235_MOESM7_ESM.zip › Figure 5/5F/pcmycThr-3min.tif]

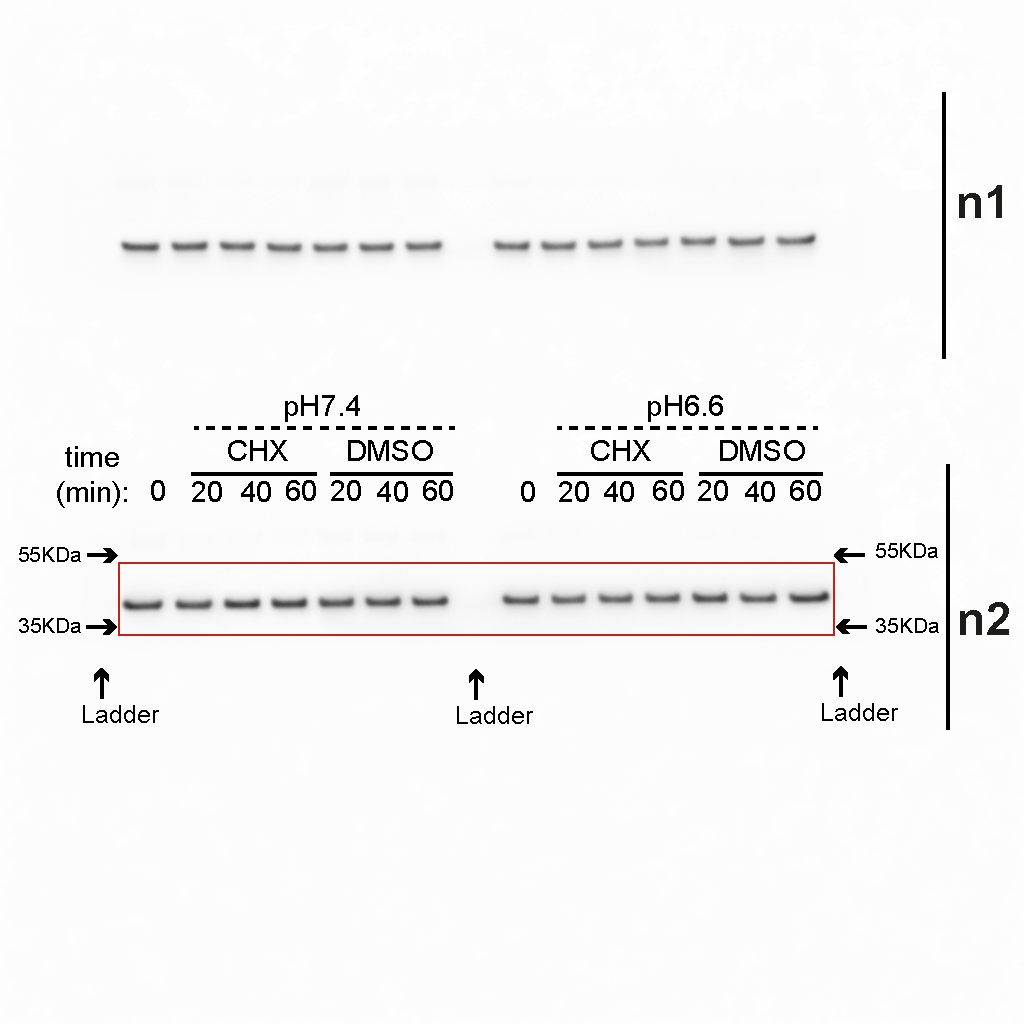

Supplement: Supplementary file 7 — Source data Fig. 5 [file 44318_2024_235_MOESM7_ESM.zip › Figure 5/5G/gapdh-3s500.tif]

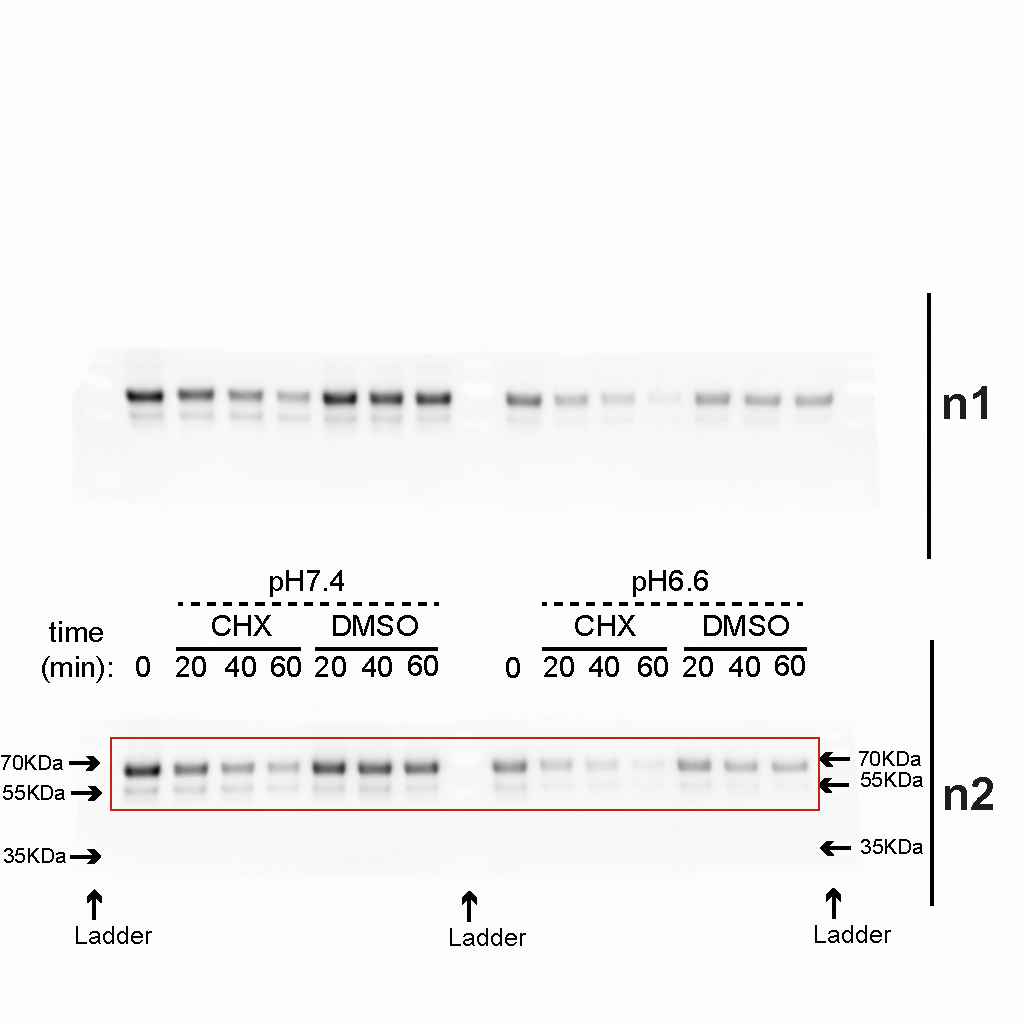

Supplement: Supplementary file 7 — Source data Fig. 5 [file 44318_2024_235_MOESM7_ESM.zip › Figure 5/5G/myc-50s.tif]

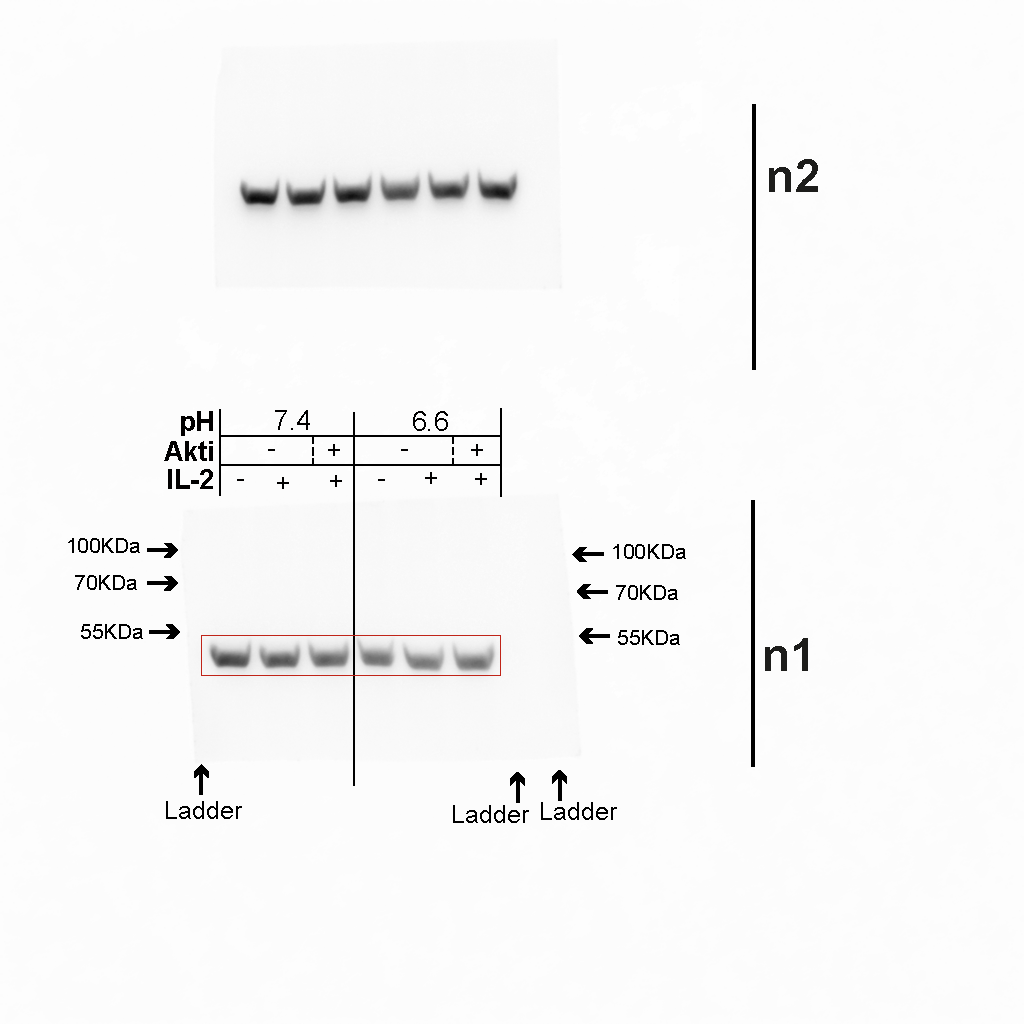

Supplement: Supplementary file 8 — Source data Fig. 6 [file 44318_2024_235_MOESM8_ESM.zip › Figure 6/6A/actin-3s.tif]

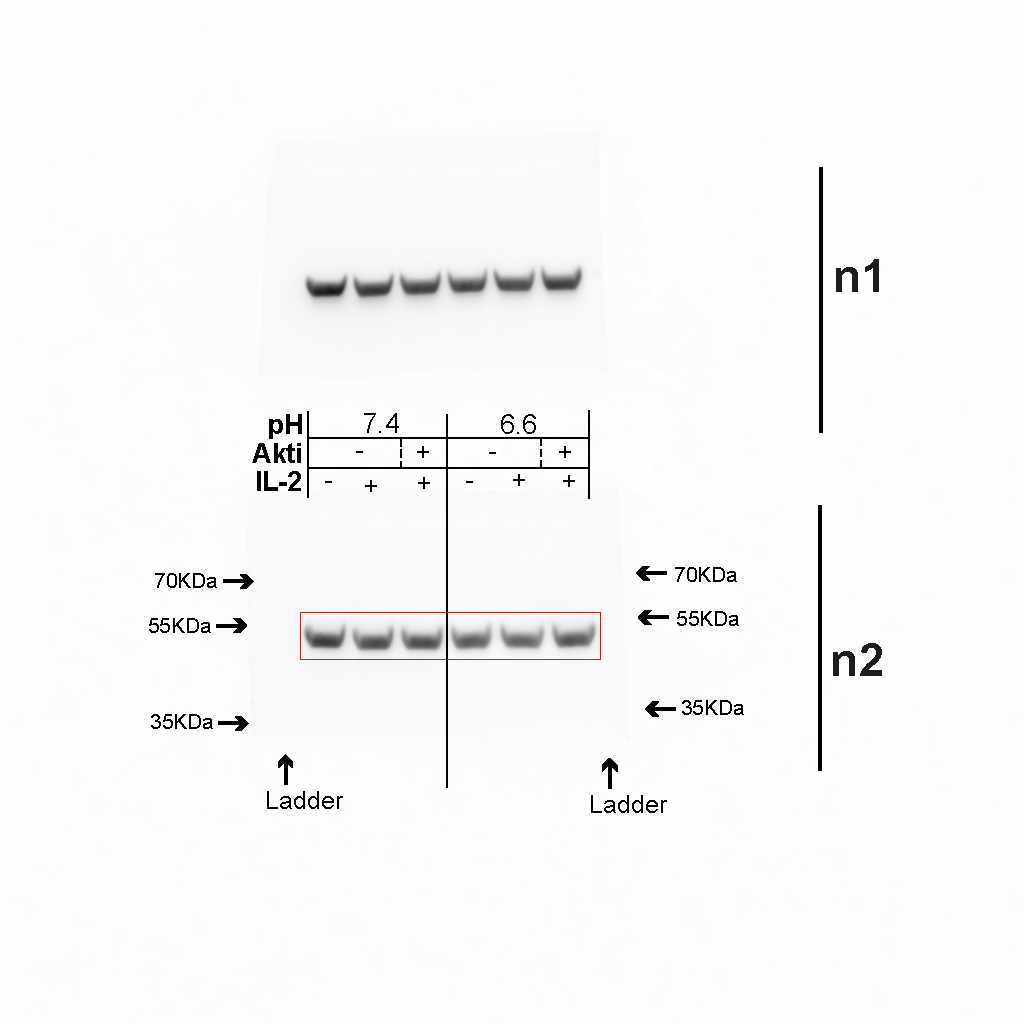

Supplement: Supplementary file 8 — Source data Fig. 6 [file 44318_2024_235_MOESM8_ESM.zip › Figure 6/6A/actin-akt-1s5.tif]

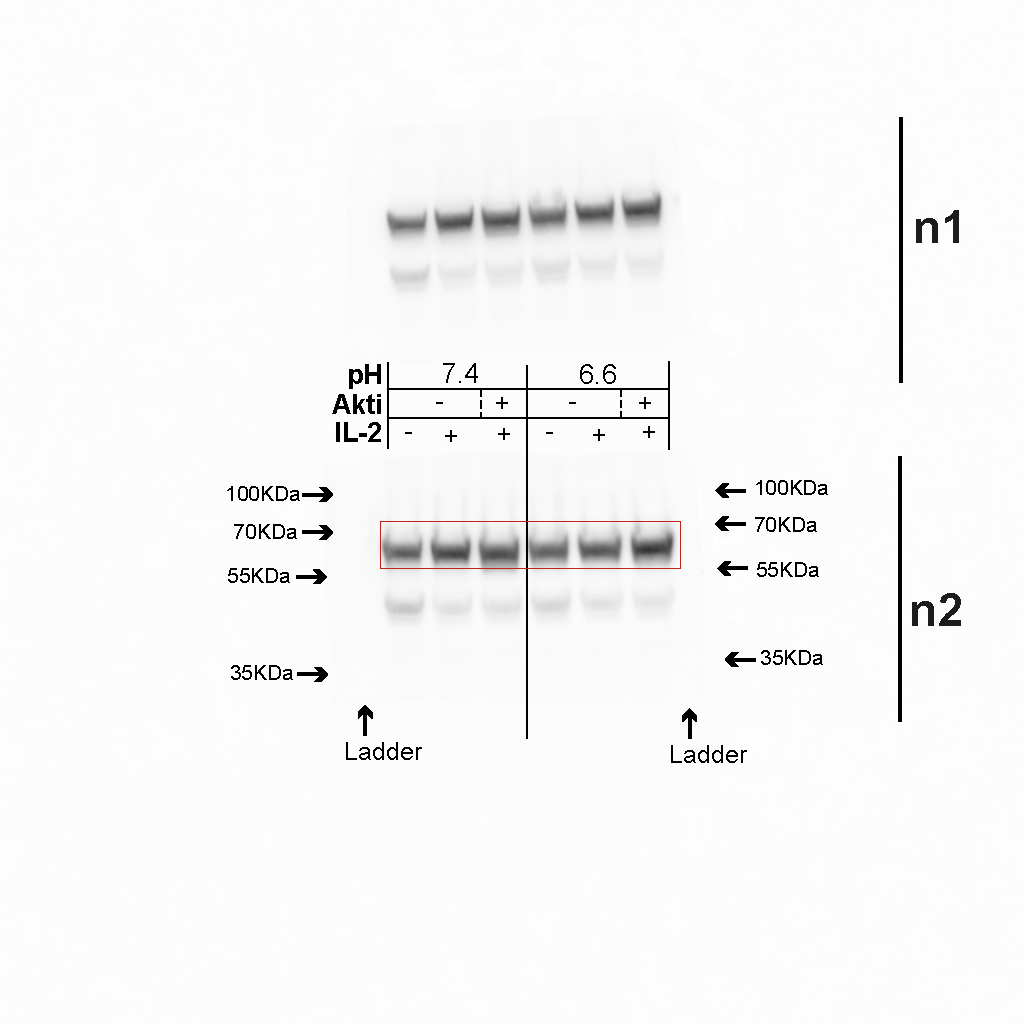

Supplement: Supplementary file 8 — Source data Fig. 6 [file 44318_2024_235_MOESM8_ESM.zip › Figure 6/6A/akt-7s.tif]

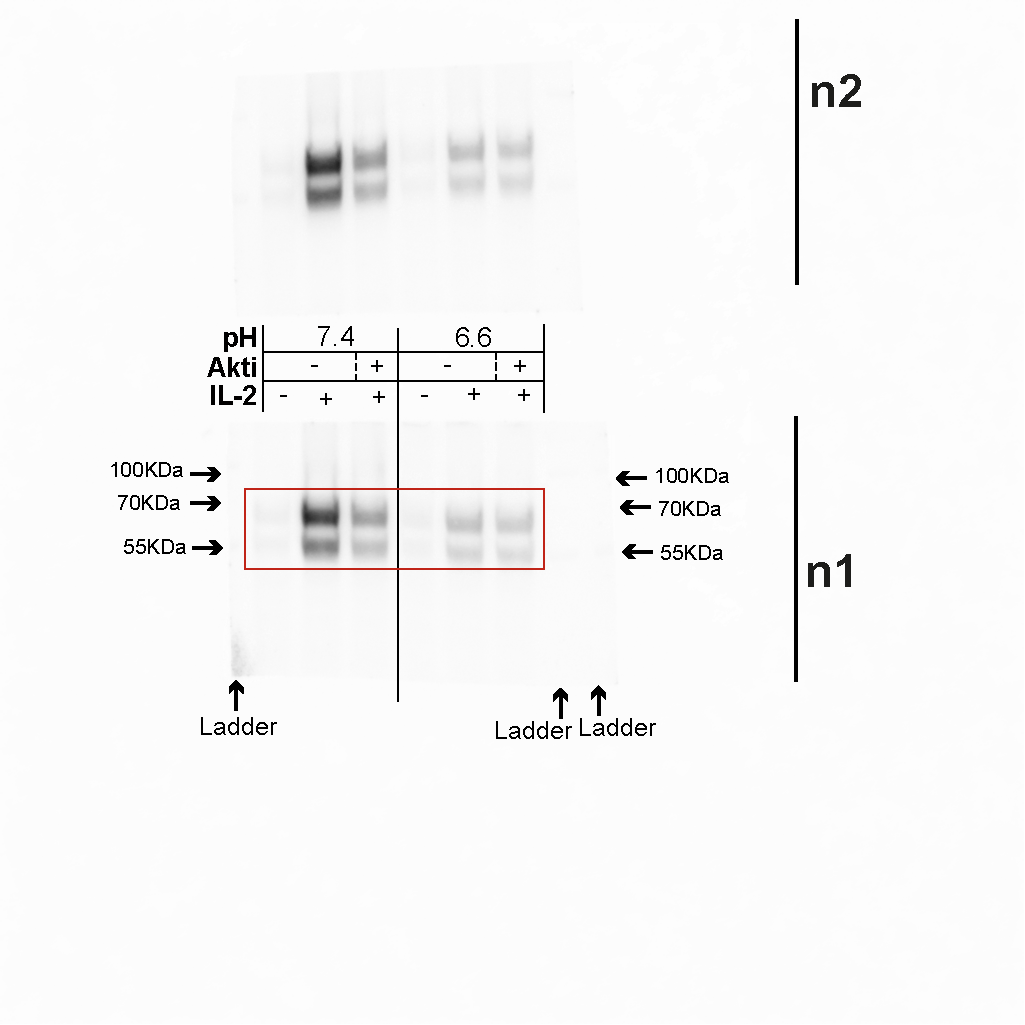

Supplement: Supplementary file 8 — Source data Fig. 6 [file 44318_2024_235_MOESM8_ESM.zip › Figure 6/6A/cmyc-20s.tif]

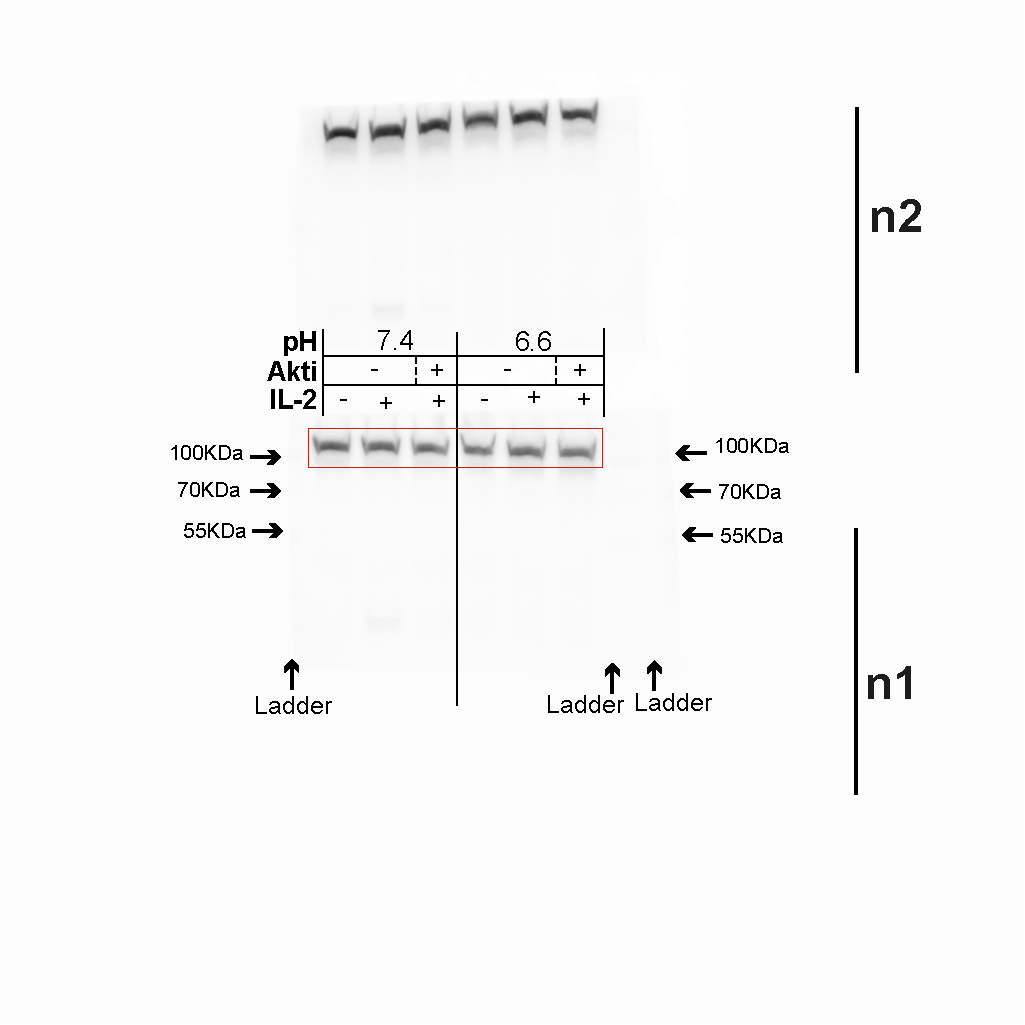

Supplement: Supplementary file 8 — Source data Fig. 6 [file 44318_2024_235_MOESM8_ESM.zip › Figure 6/6A/foxo-600ms.tif]

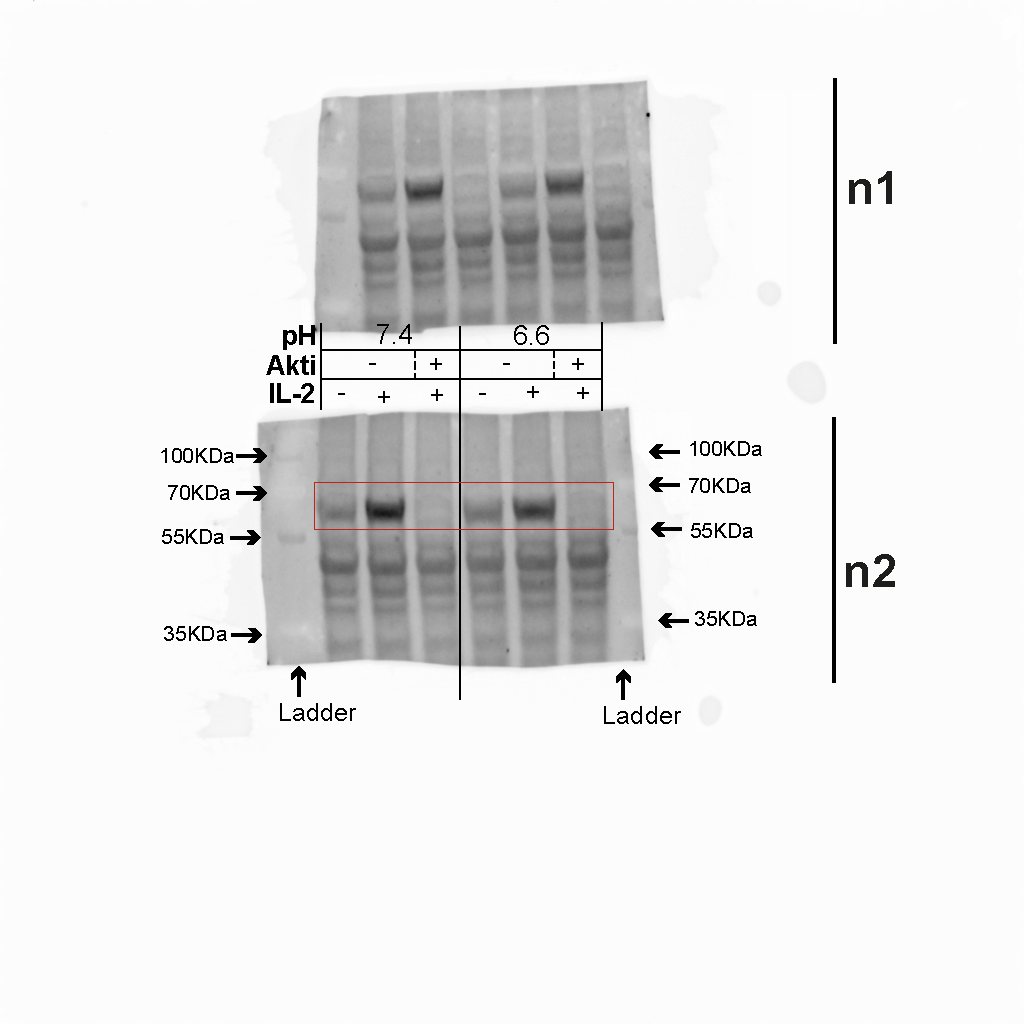

Supplement: Supplementary file 8 — Source data Fig. 6 [file 44318_2024_235_MOESM8_ESM.zip › Figure 6/6A/p-aktThr-5min.tif]

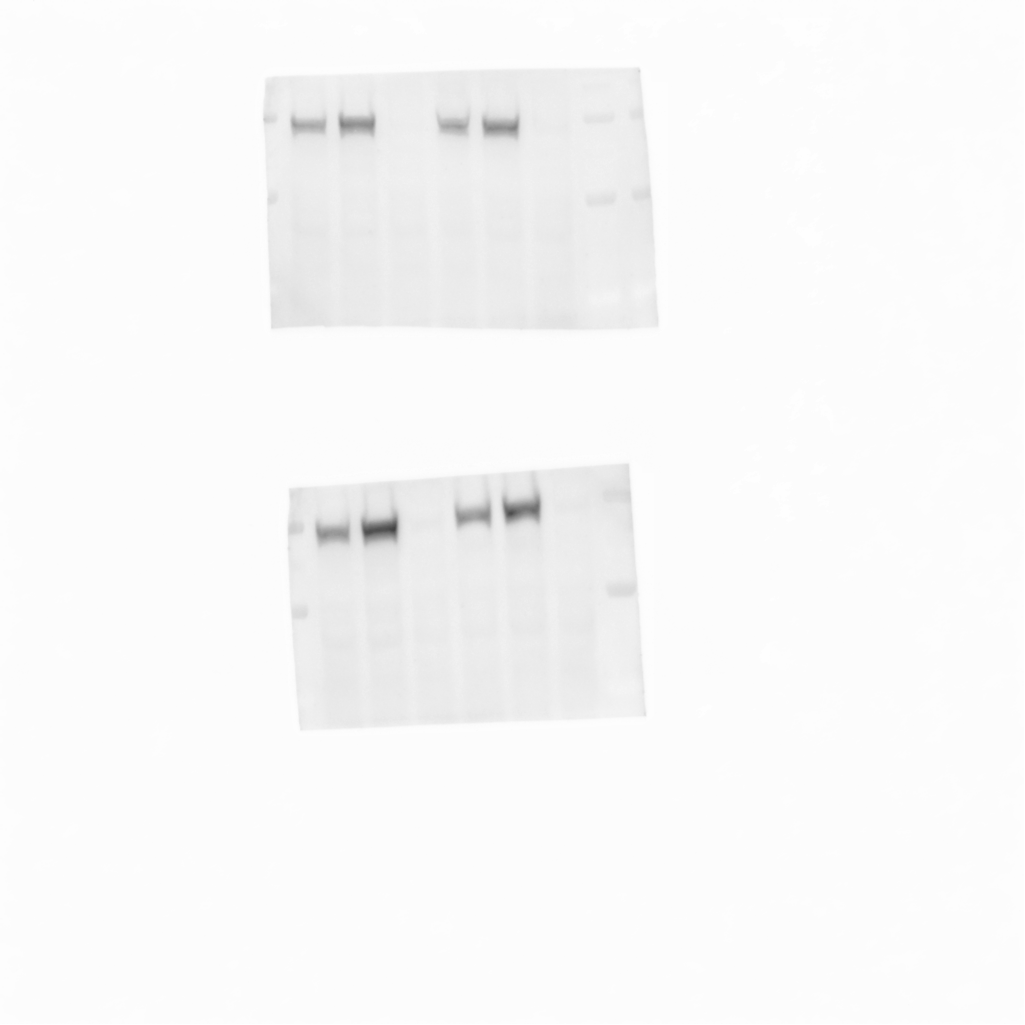

Supplement: Supplementary file 8 — Source data Fig. 6 [file 44318_2024_235_MOESM8_ESM.zip › Figure 6/6A/p-foxo-45s.tif]

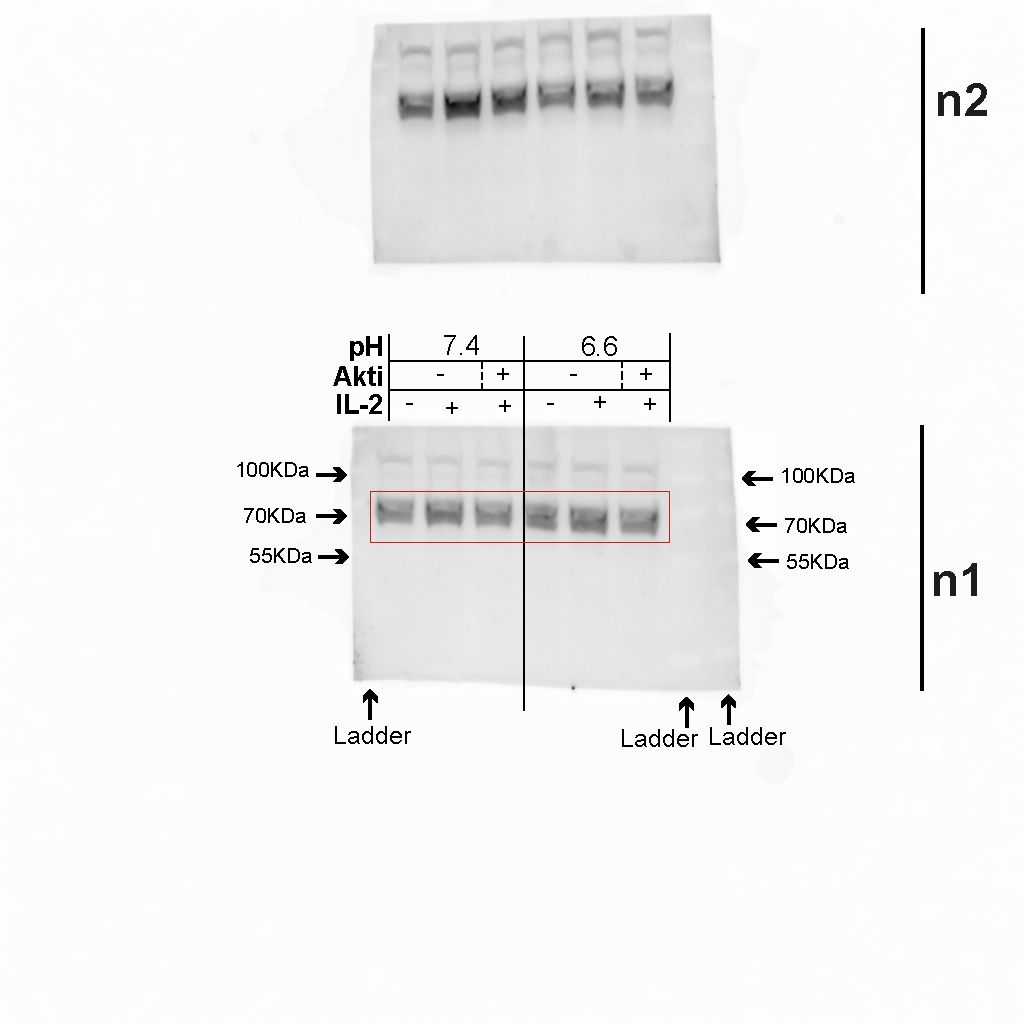

Supplement: Supplementary file 8 — Source data Fig. 6 [file 44318_2024_235_MOESM8_ESM.zip › Figure 6/6A/p70s6k-2min10.tif]

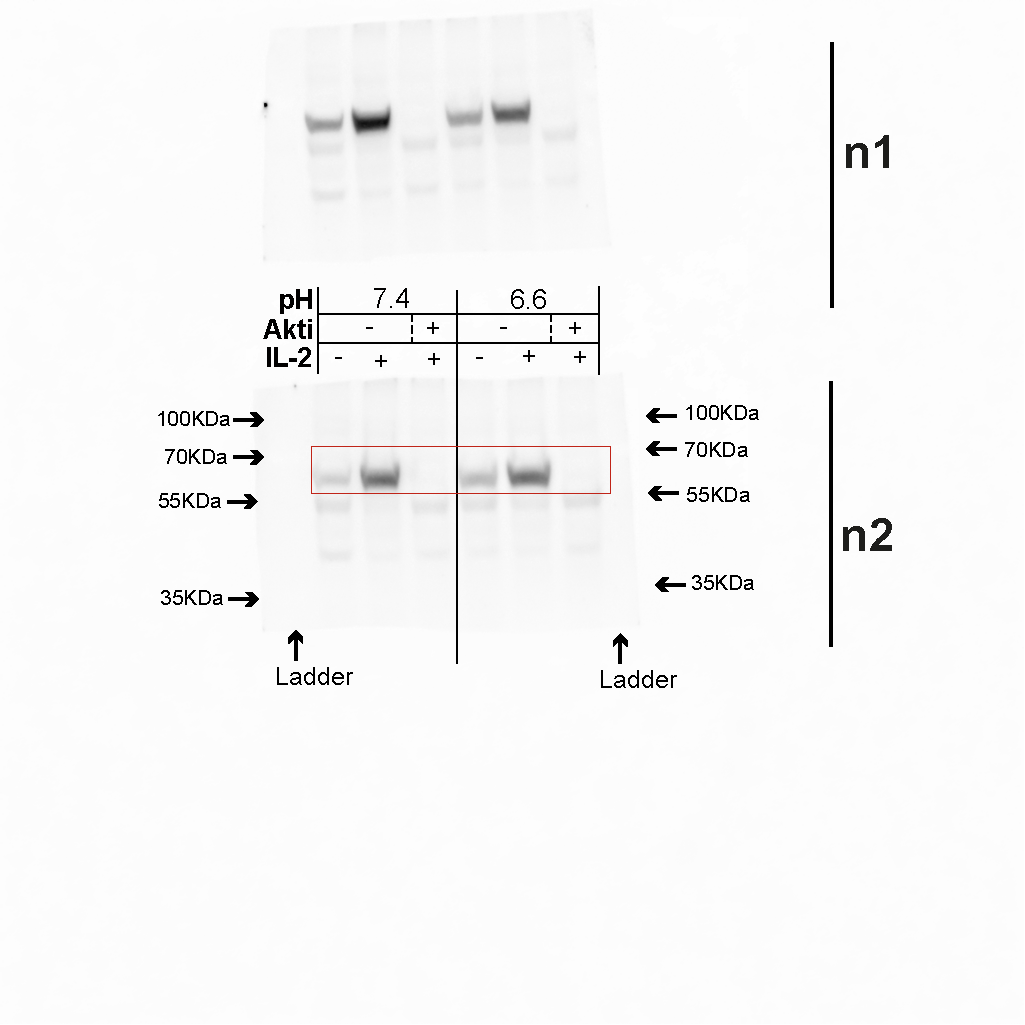

Supplement: Supplementary file 8 — Source data Fig. 6 [file 44318_2024_235_MOESM8_ESM.zip › Figure 6/6A/paktSer-40s.tif]

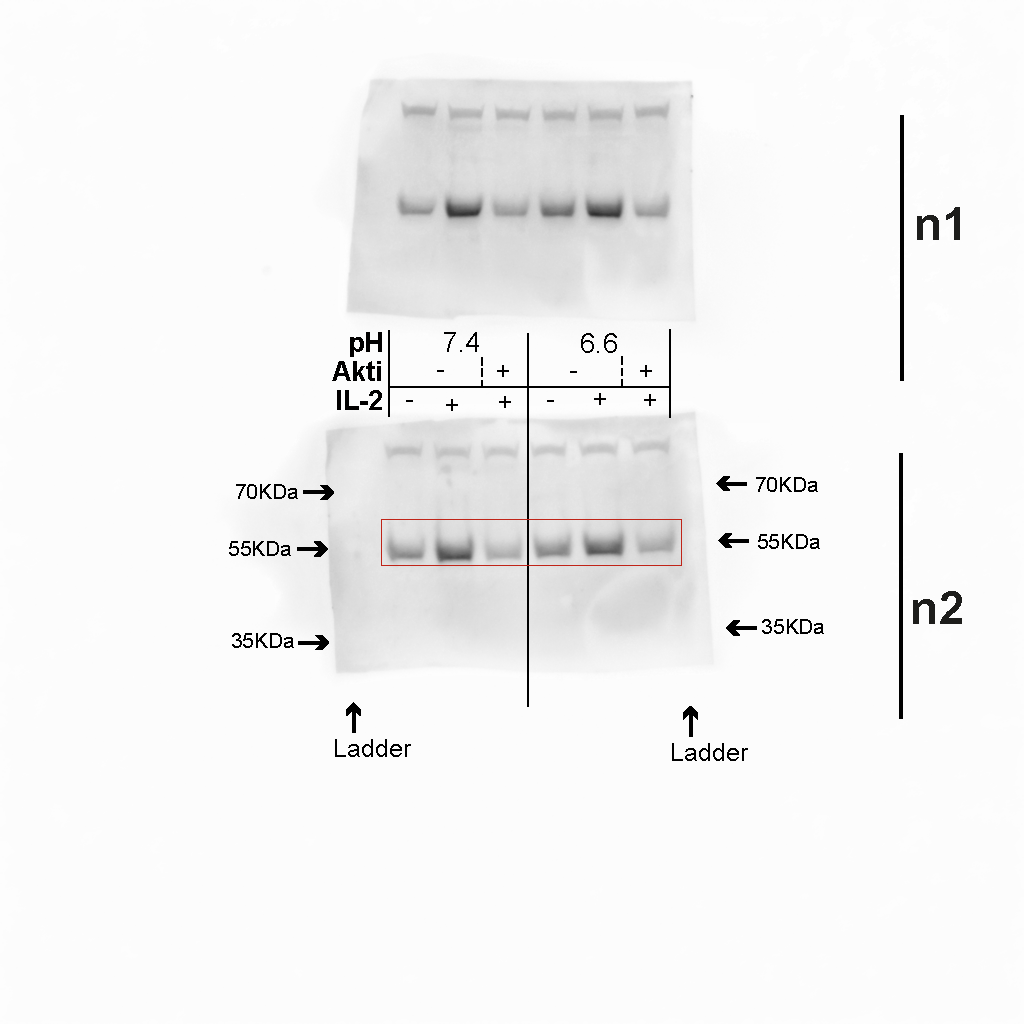

Supplement: Supplementary file 8 — Source data Fig. 6 [file 44318_2024_235_MOESM8_ESM.zip › Figure 6/6A/pgks3-15s.tif]

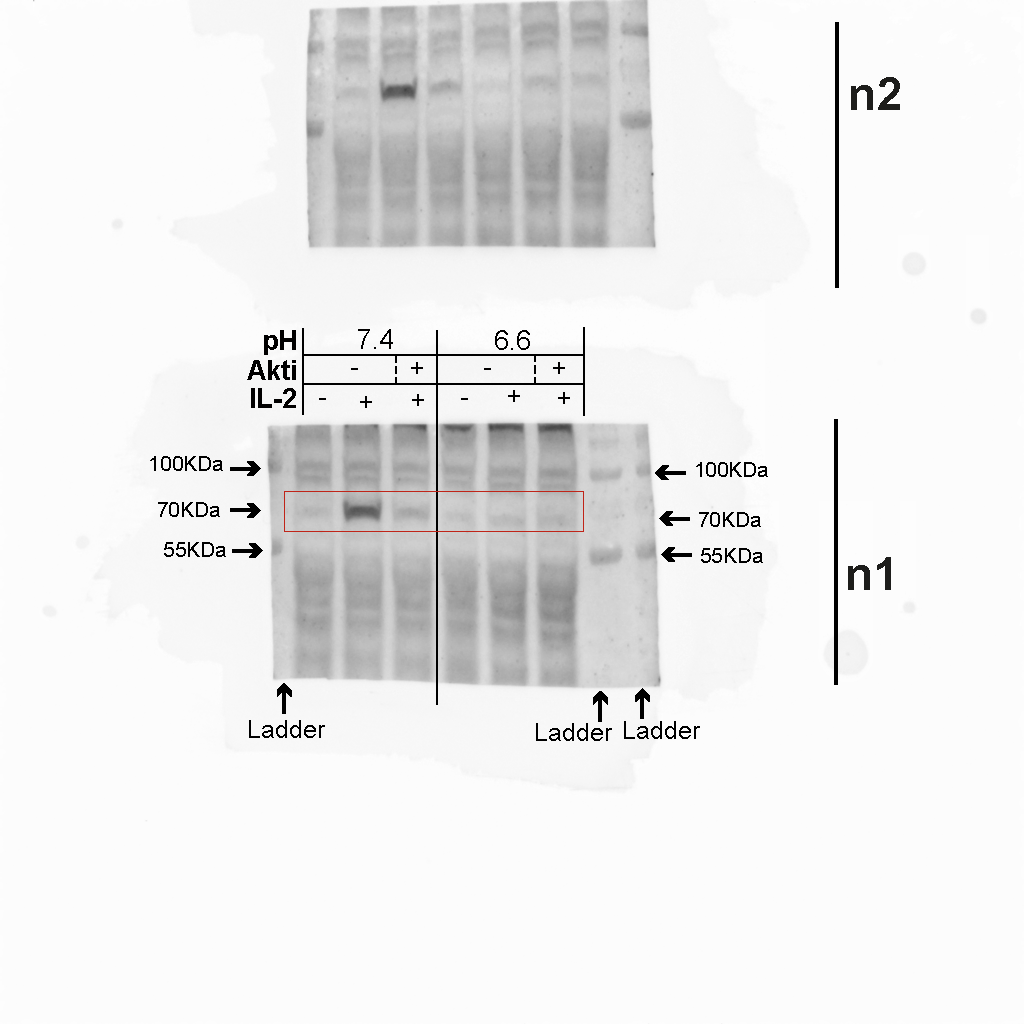

Supplement: Supplementary file 8 — Source data Fig. 6 [file 44318_2024_235_MOESM8_ESM.zip › Figure 6/6A/pp70s6k-6min15.tif]

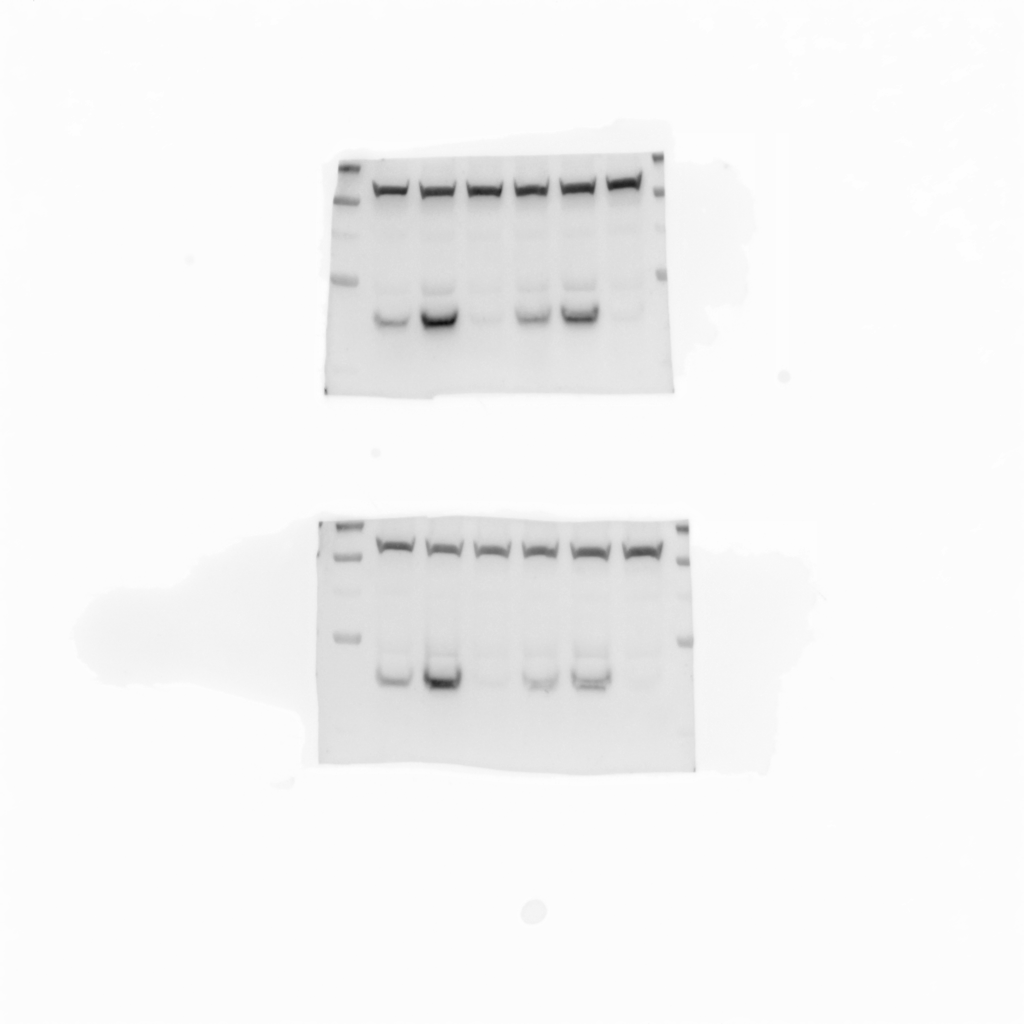

Supplement: Supplementary file 8 — Source data Fig. 6 [file 44318_2024_235_MOESM8_ESM.zip › Figure 6/6A/ppras40-2min10.tif]

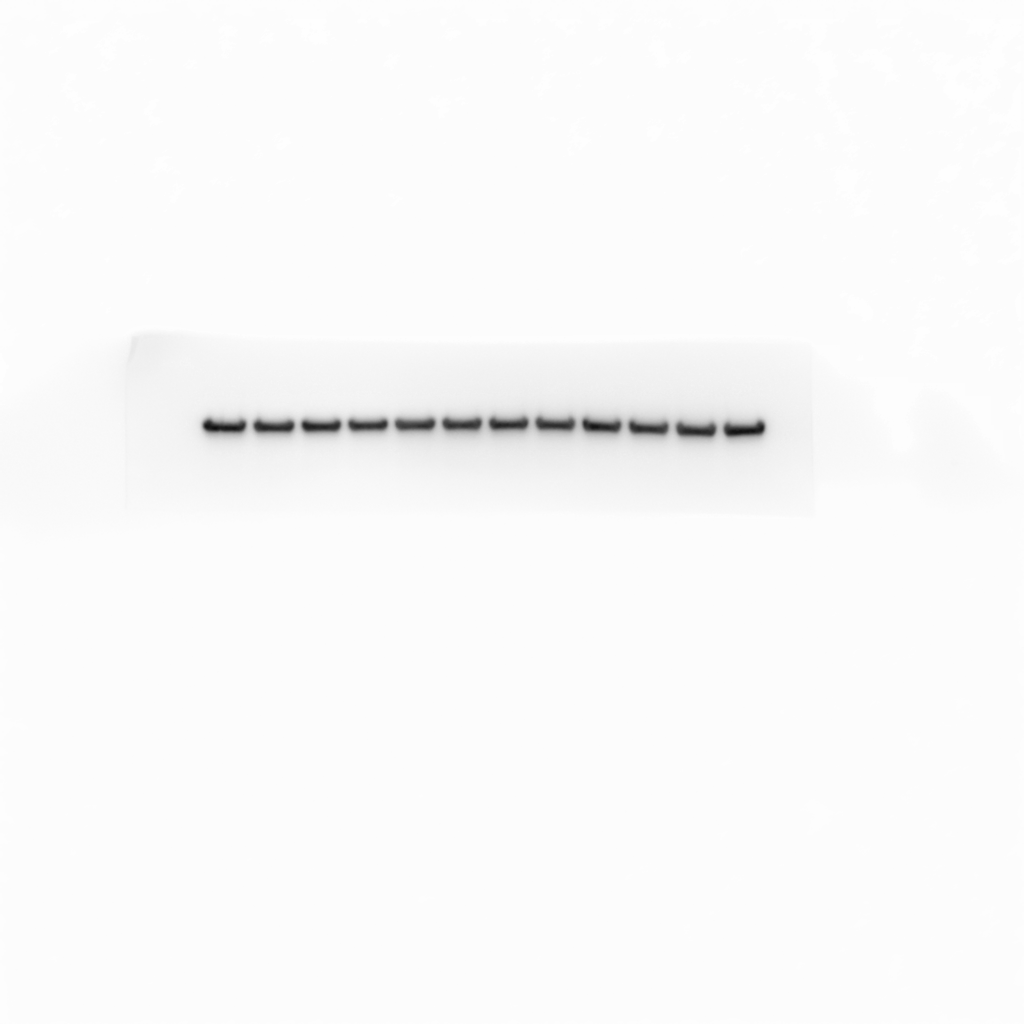

Supplement: Supplementary file 8 — Source data Fig. 6 [file 44318_2024_235_MOESM8_ESM.zip › Figure 6/6B/gapdh-4s5.tif]

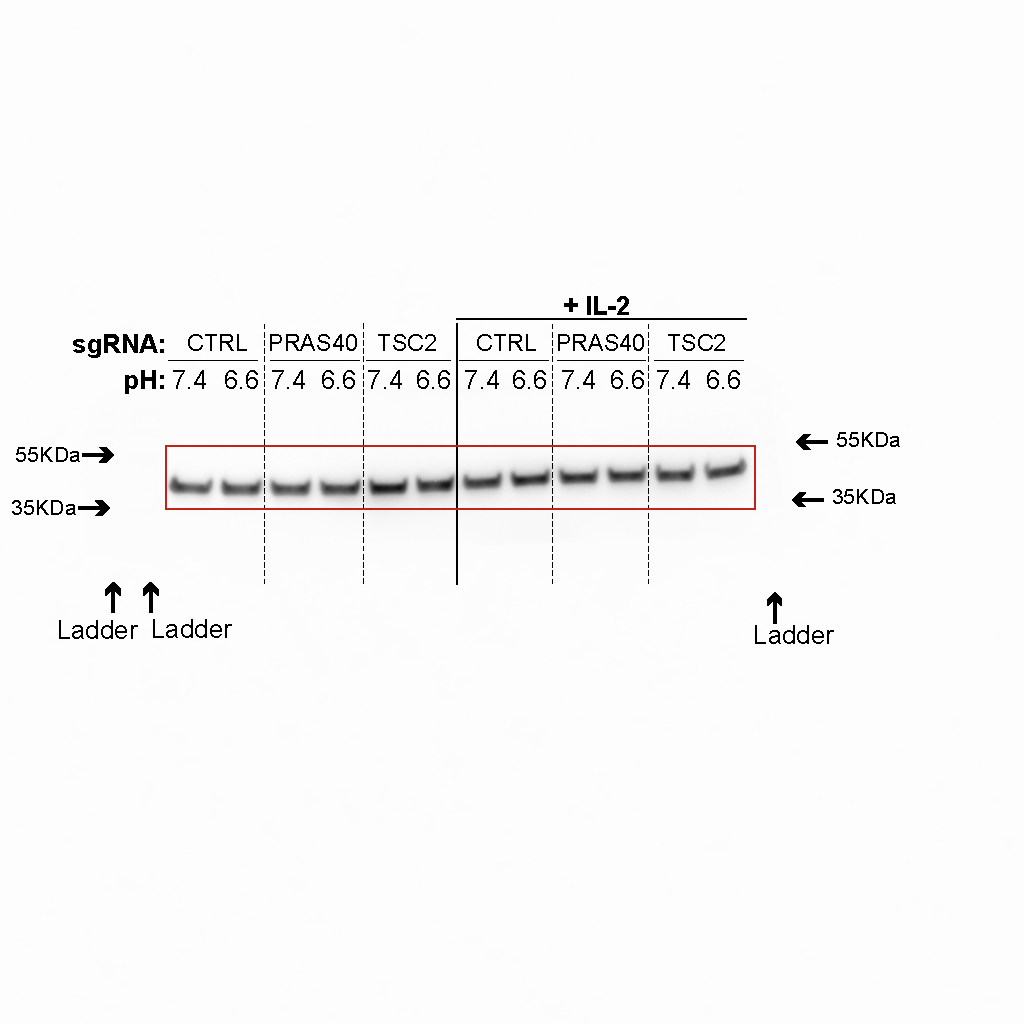

Supplement: Supplementary file 8 — Source data Fig. 6 [file 44318_2024_235_MOESM8_ESM.zip › Figure 6/6B/gapdh-myc-4s.tif]

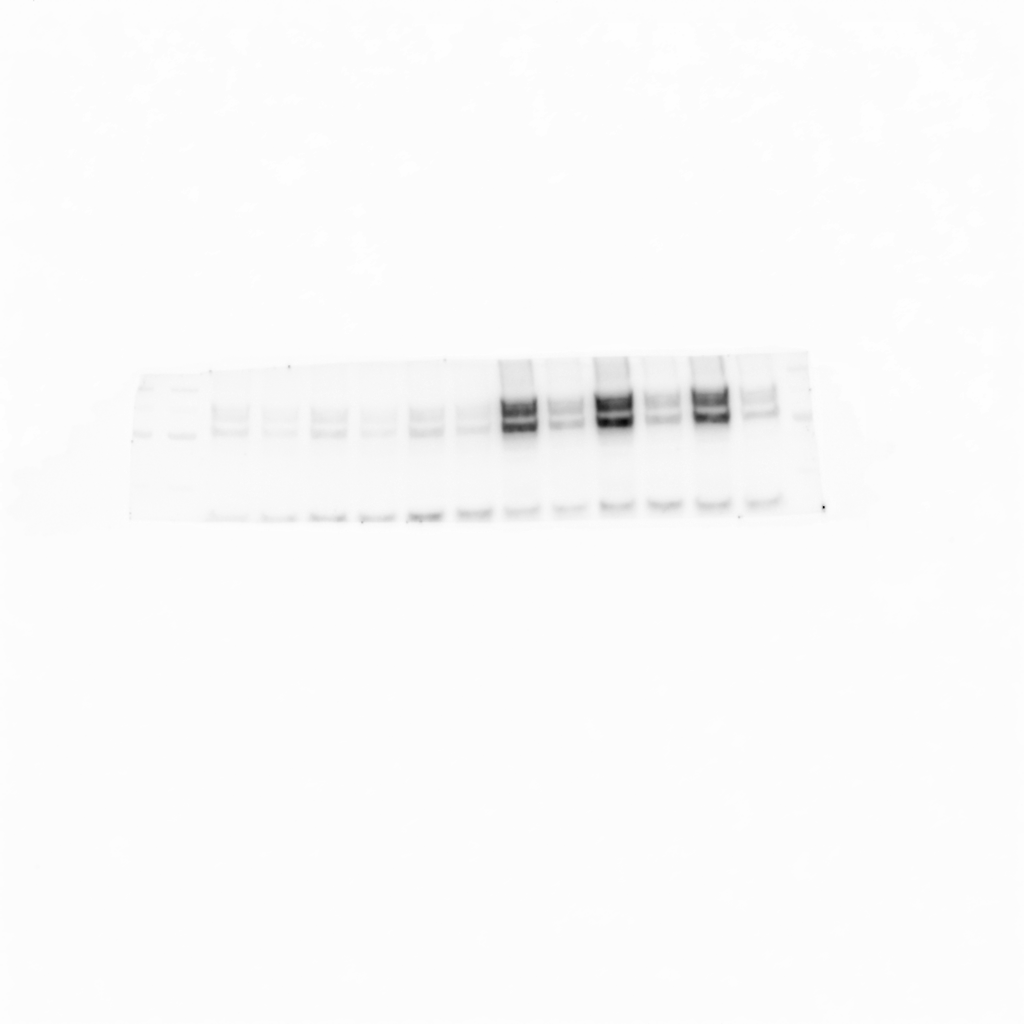

Supplement: Supplementary file 8 — Source data Fig. 6 [file 44318_2024_235_MOESM8_ESM.zip › Figure 6/6B/myc-2min15.tif]

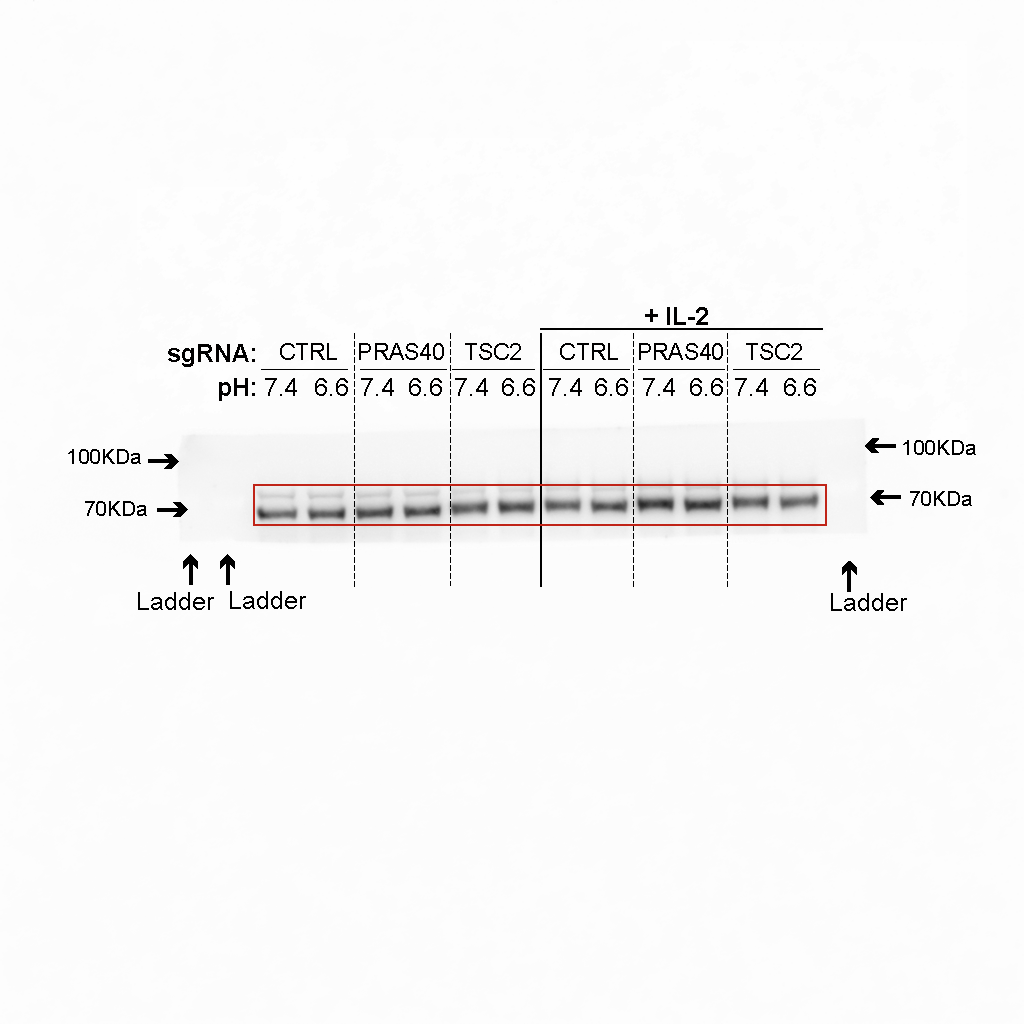

Supplement: Supplementary file 8 — Source data Fig. 6 [file 44318_2024_235_MOESM8_ESM.zip › Figure 6/6B/p70-50s.tif]

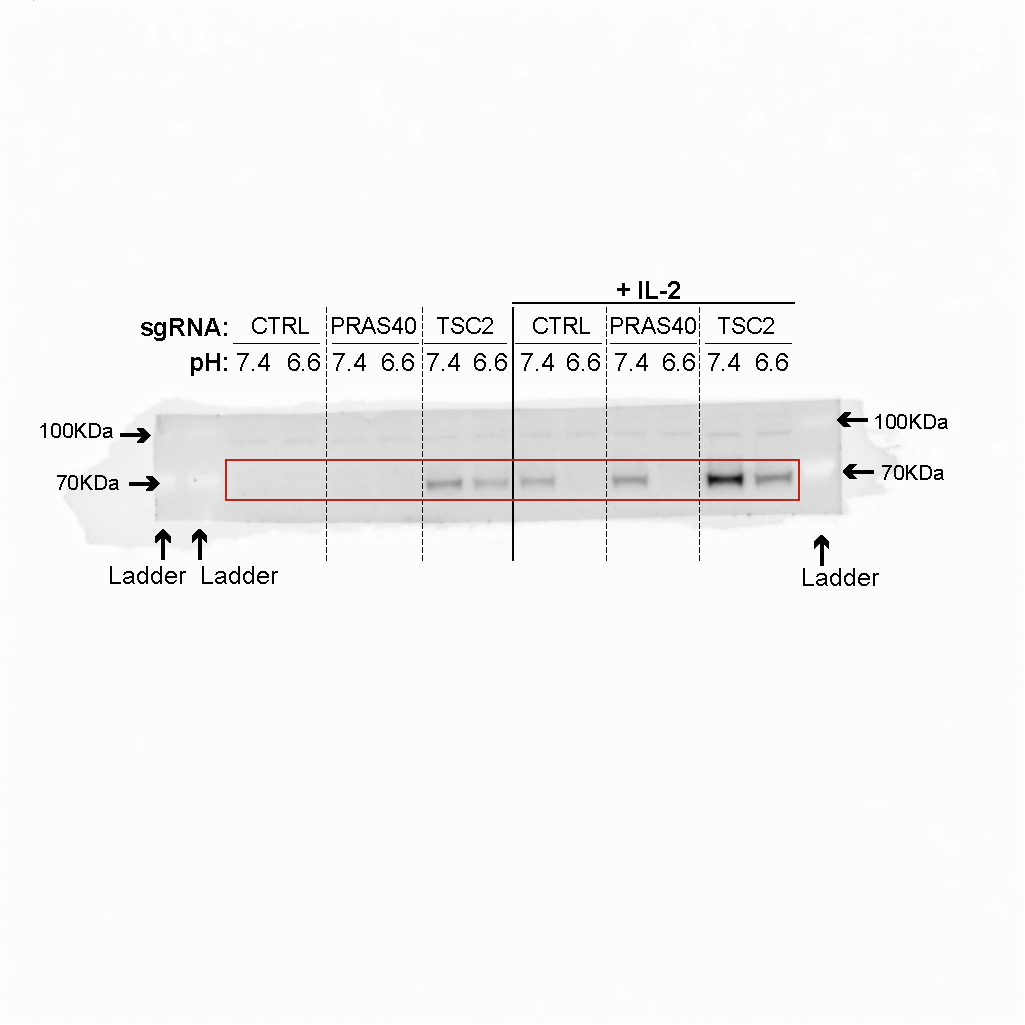

Supplement: Supplementary file 8 — Source data Fig. 6 [file 44318_2024_235_MOESM8_ESM.zip › Figure 6/6B/pp70s6k-6min30.tif]

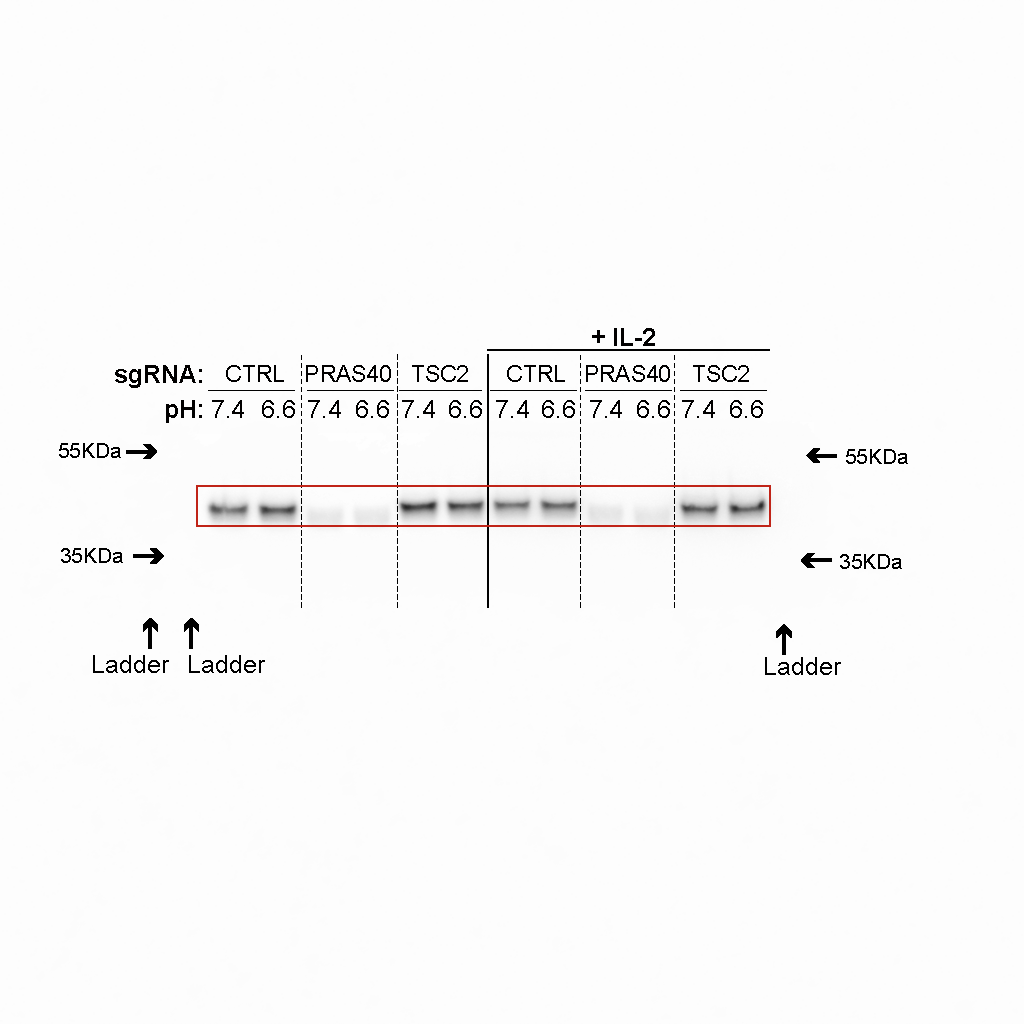

Supplement: Supplementary file 8 — Source data Fig. 6 [file 44318_2024_235_MOESM8_ESM.zip › Figure 6/6B/pras-6s.tif]

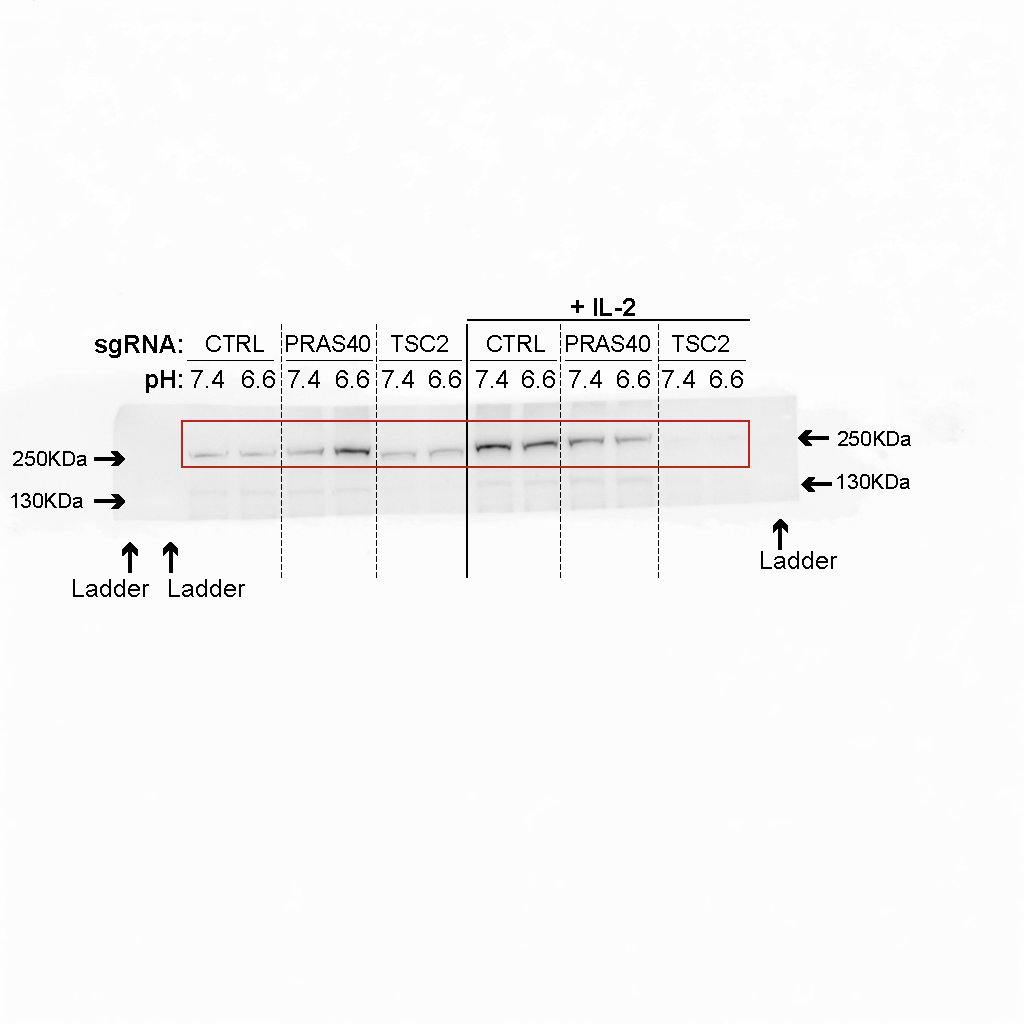

Supplement: Supplementary file 8 — Source data Fig. 6 [file 44318_2024_235_MOESM8_ESM.zip › Figure 6/6B/tsc-2min30.tif]

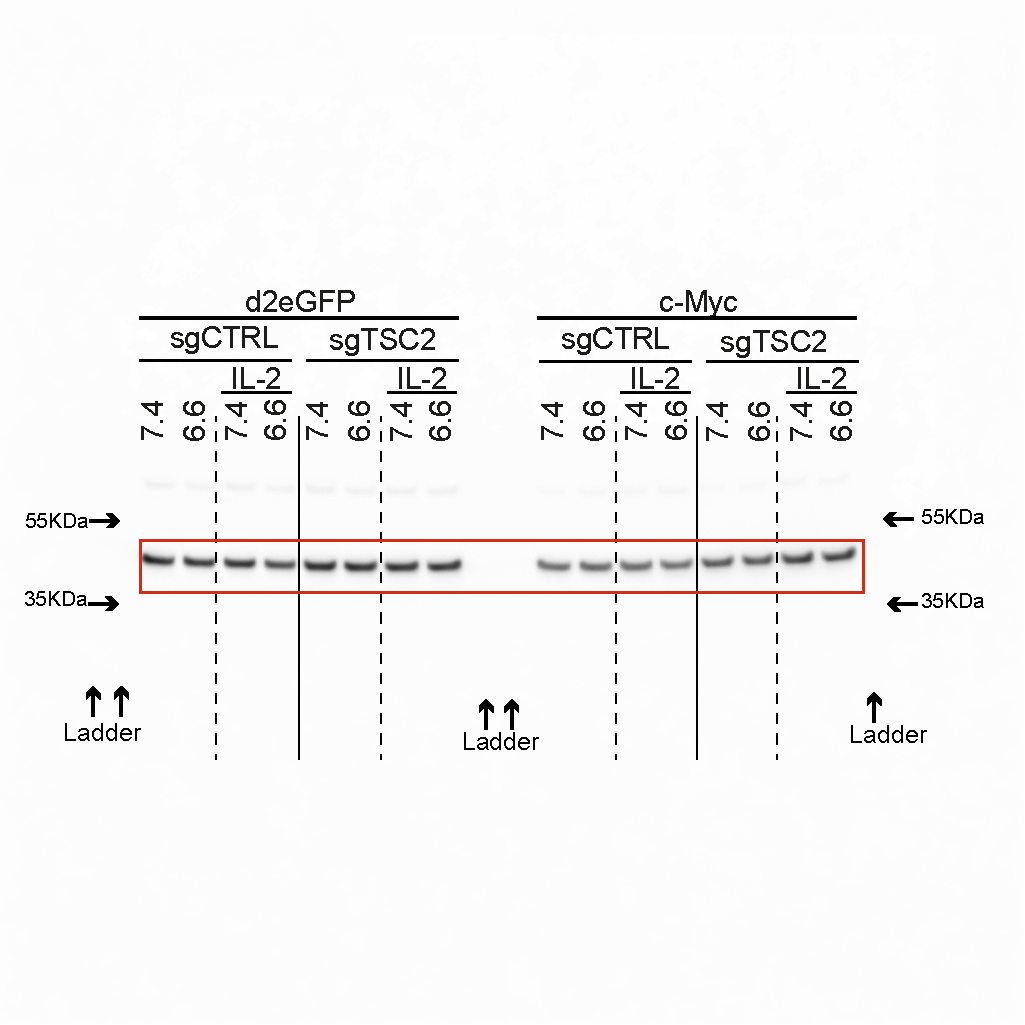

Supplement: Supplementary file 8 — Source data Fig. 6 [file 44318_2024_235_MOESM8_ESM.zip › Figure 6/6D/gapdh-4s.tif]

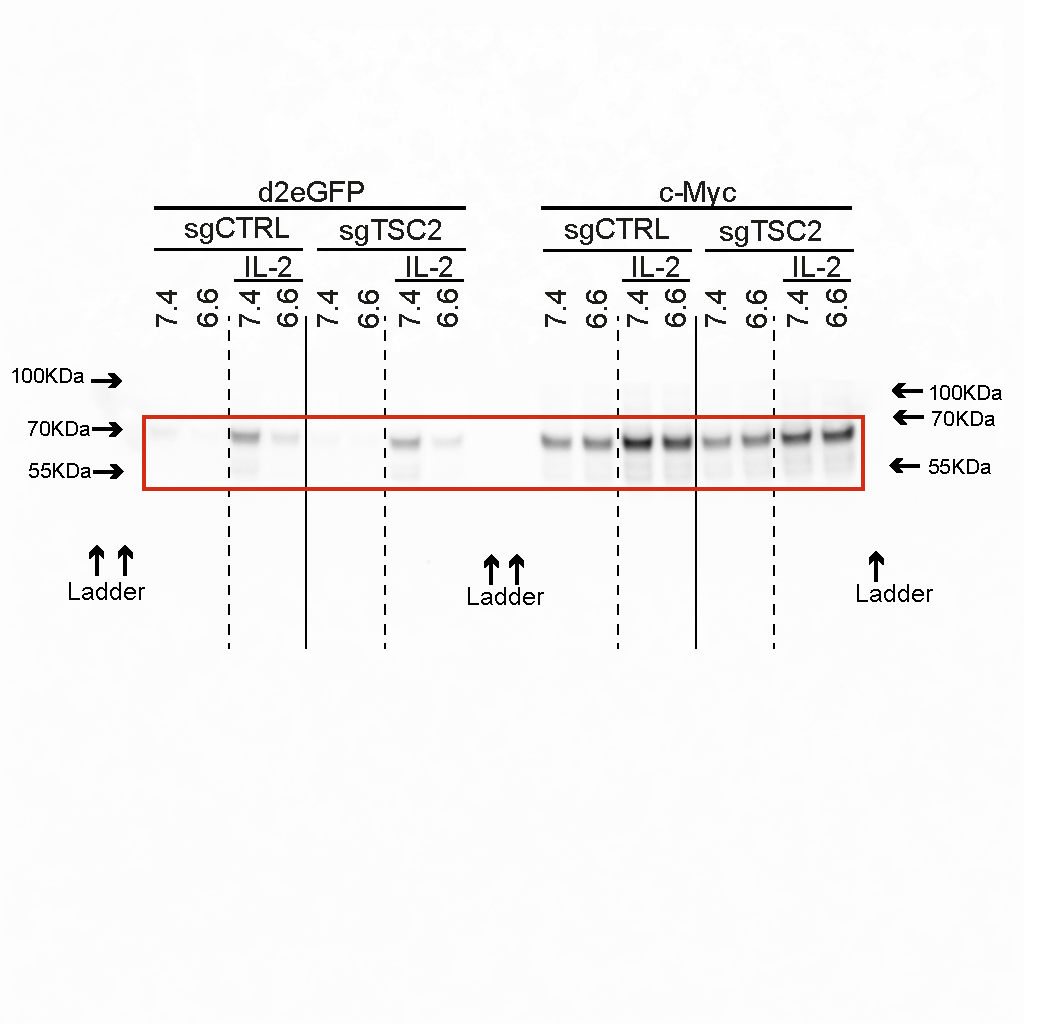

Supplement: Supplementary file 8 — Source data Fig. 6 [file 44318_2024_235_MOESM8_ESM.zip › Figure 6/6D/myc-18s.tif]

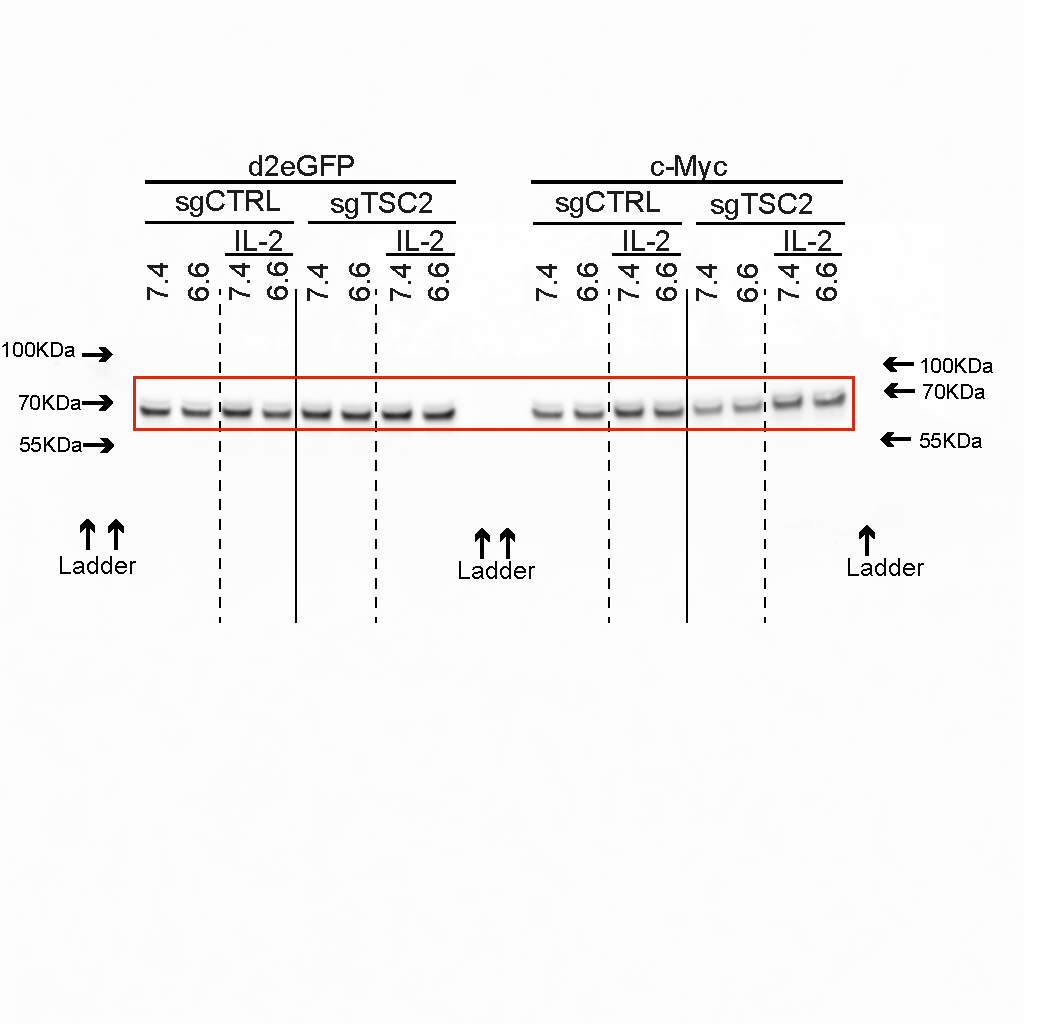

Supplement: Supplementary file 8 — Source data Fig. 6 [file 44318_2024_235_MOESM8_ESM.zip › Figure 6/6D/p70-15s.tif]

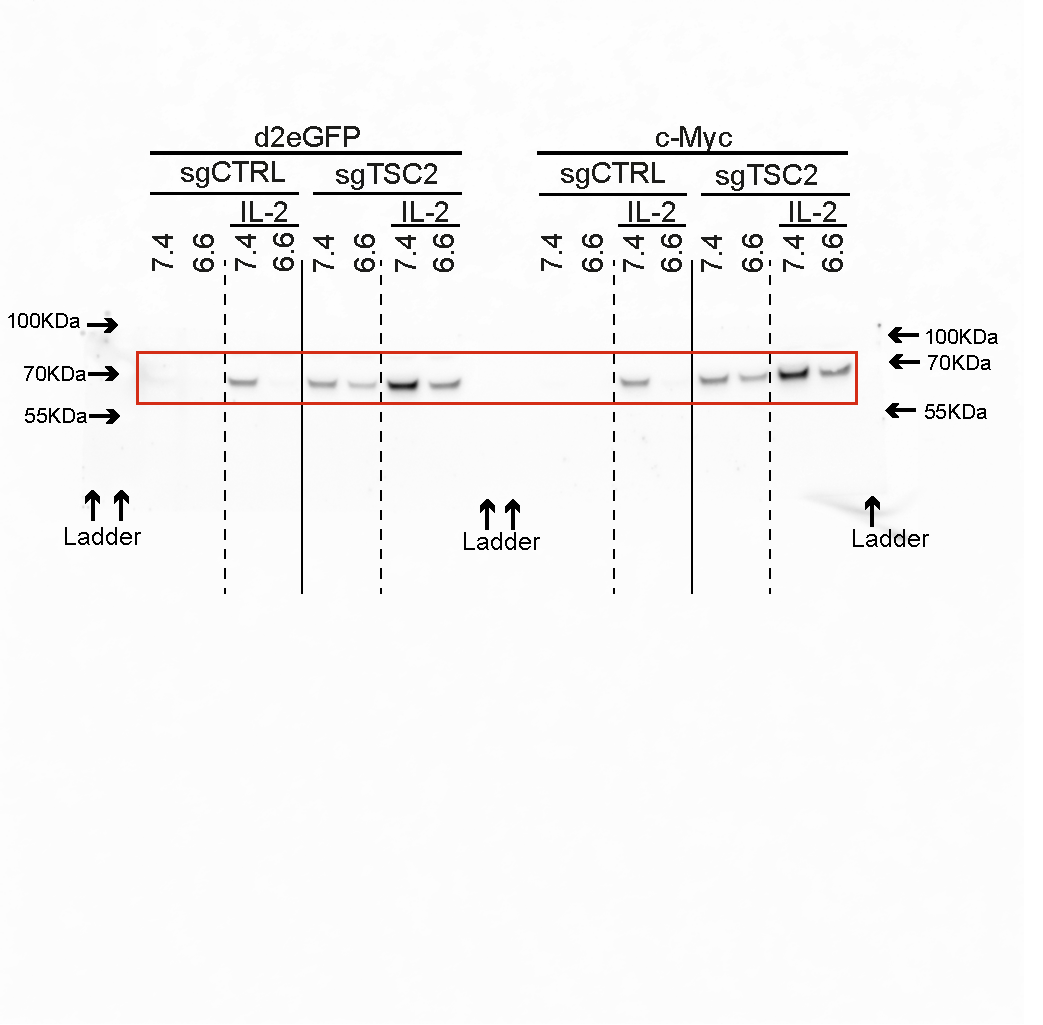

Supplement: Supplementary file 8 — Source data Fig. 6 [file 44318_2024_235_MOESM8_ESM.zip › Figure 6/6D/pp70-1min.tif]

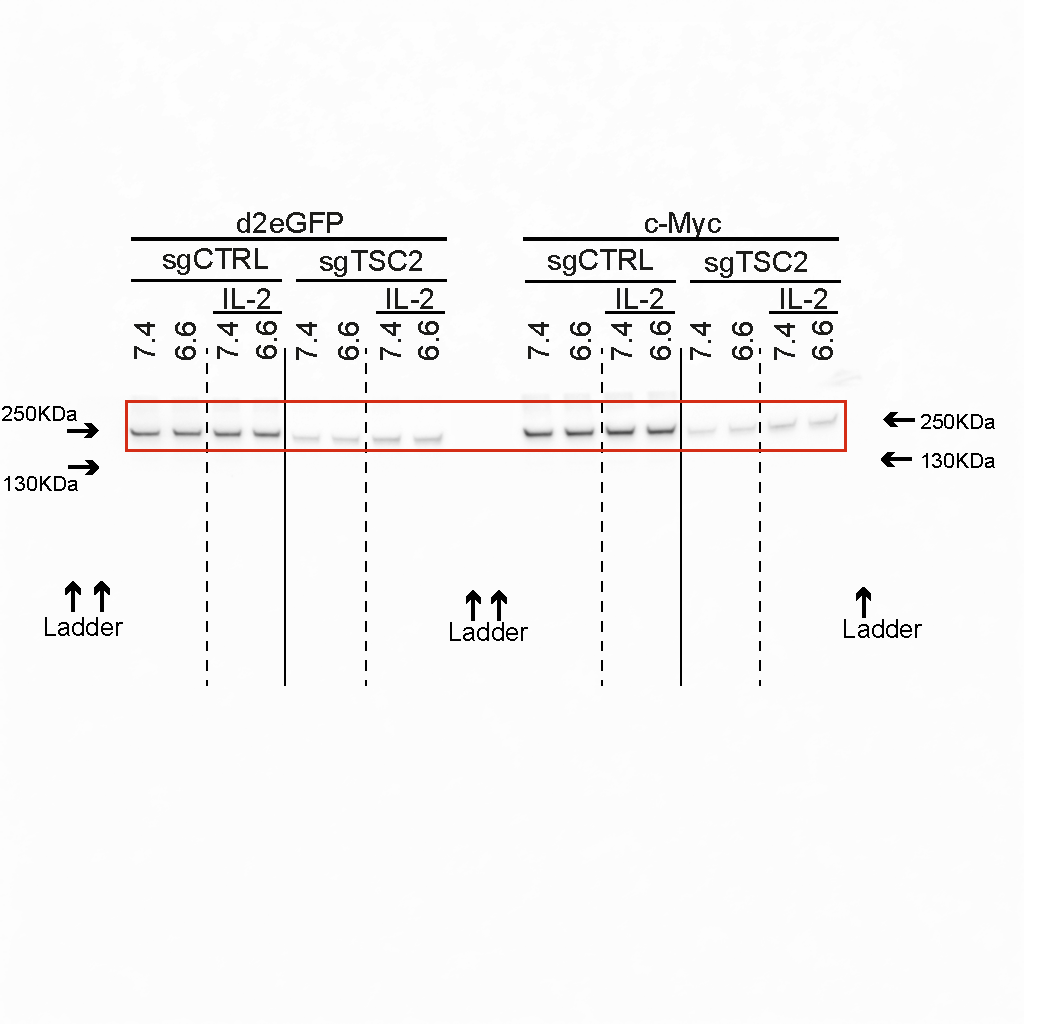

Supplement: Supplementary file 8 — Source data Fig. 6 [file 44318_2024_235_MOESM8_ESM.zip › Figure 6/6D/tsc-8s.tif]

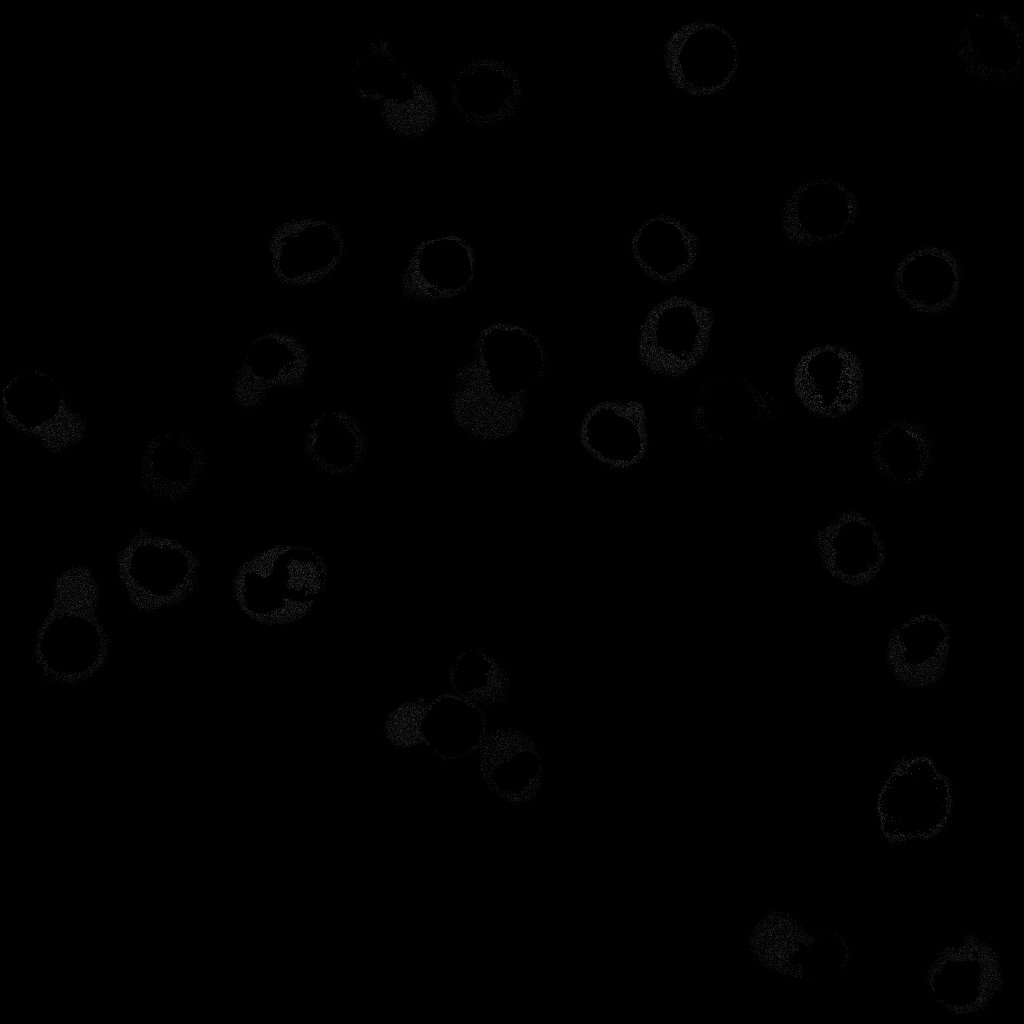

Supplement: Supplementary file 9 — Source data Fig. 7 [file 44318_2024_235_MOESM9_ESM.zip › Figure 7/7A/pH6_3-std-green.jpg]

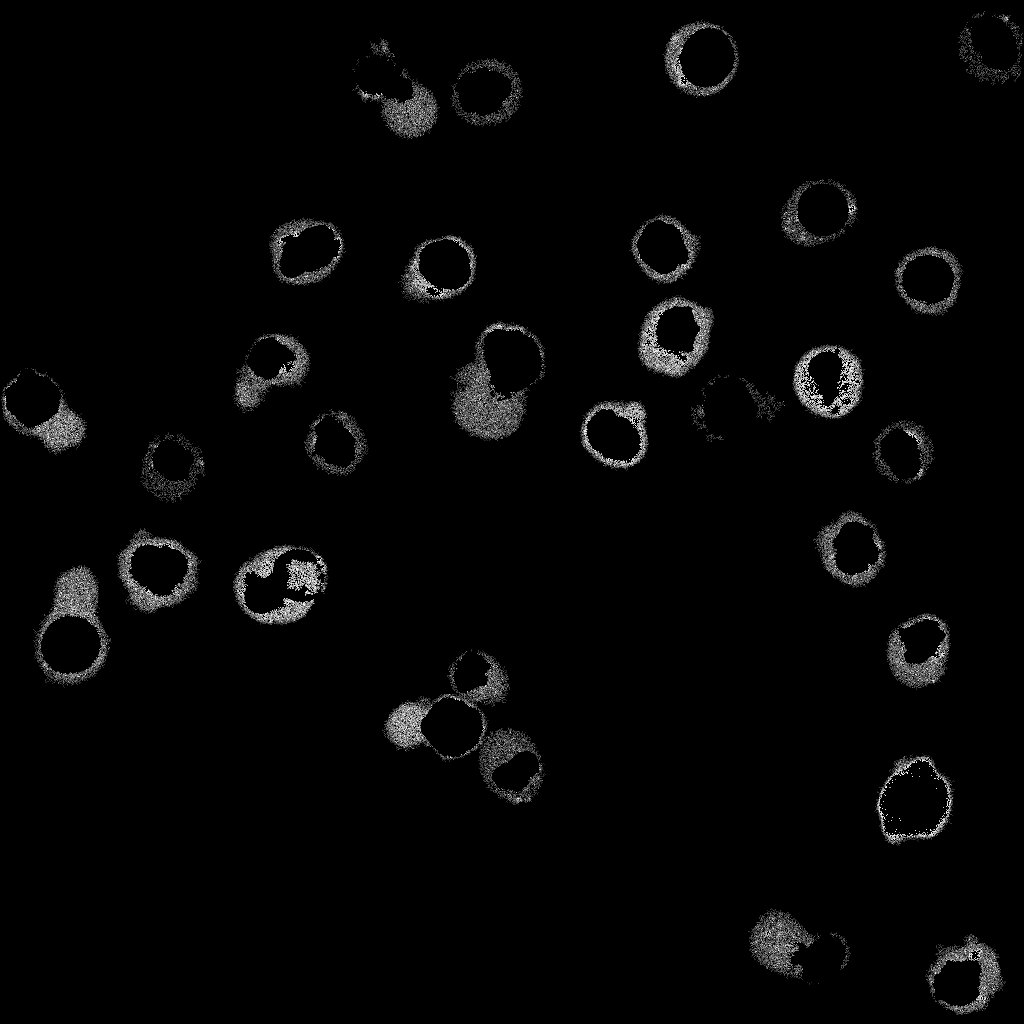

Supplement: Supplementary file 9 — Source data Fig. 7 [file 44318_2024_235_MOESM9_ESM.zip › Figure 7/7A/pH6_3-std-red.jpg]

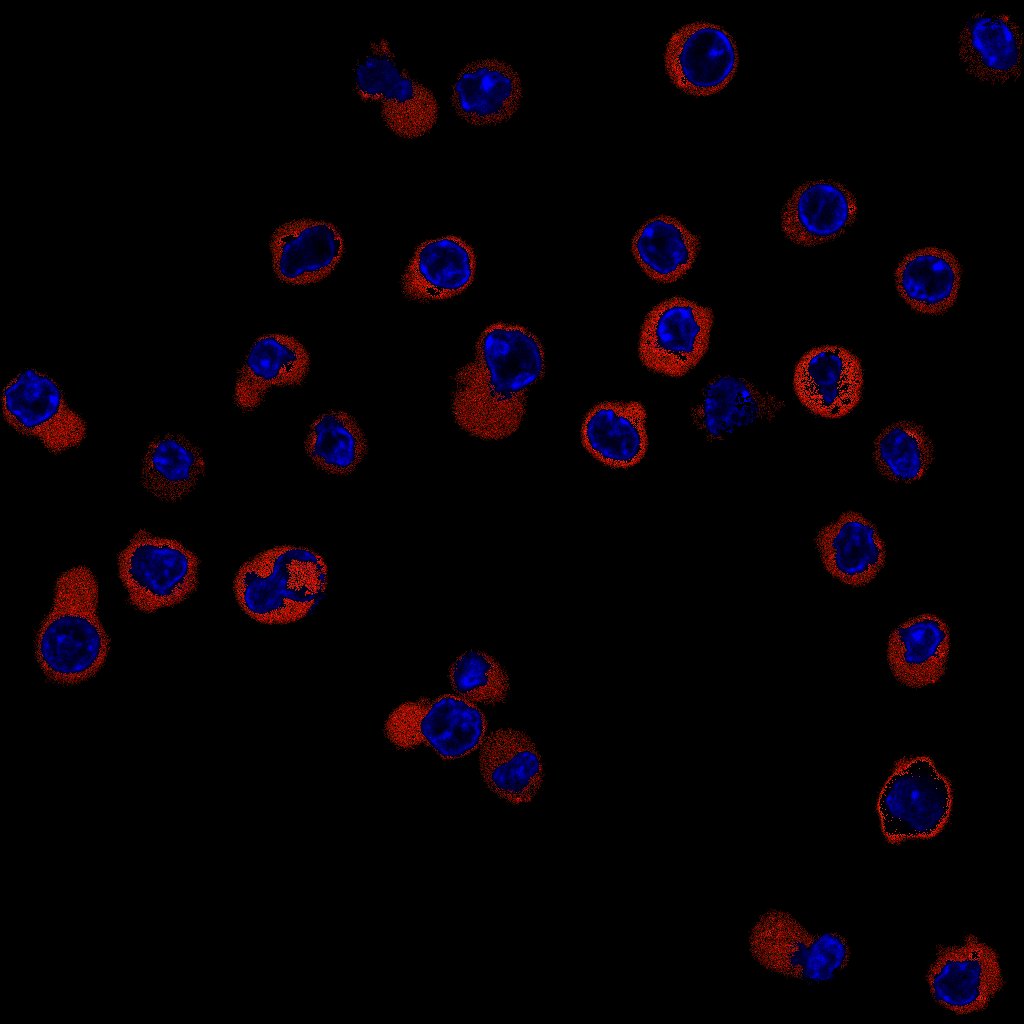

Supplement: Supplementary file 9 — Source data Fig. 7 [file 44318_2024_235_MOESM9_ESM.zip › Figure 7/7A/pH6_3-std.jpg]

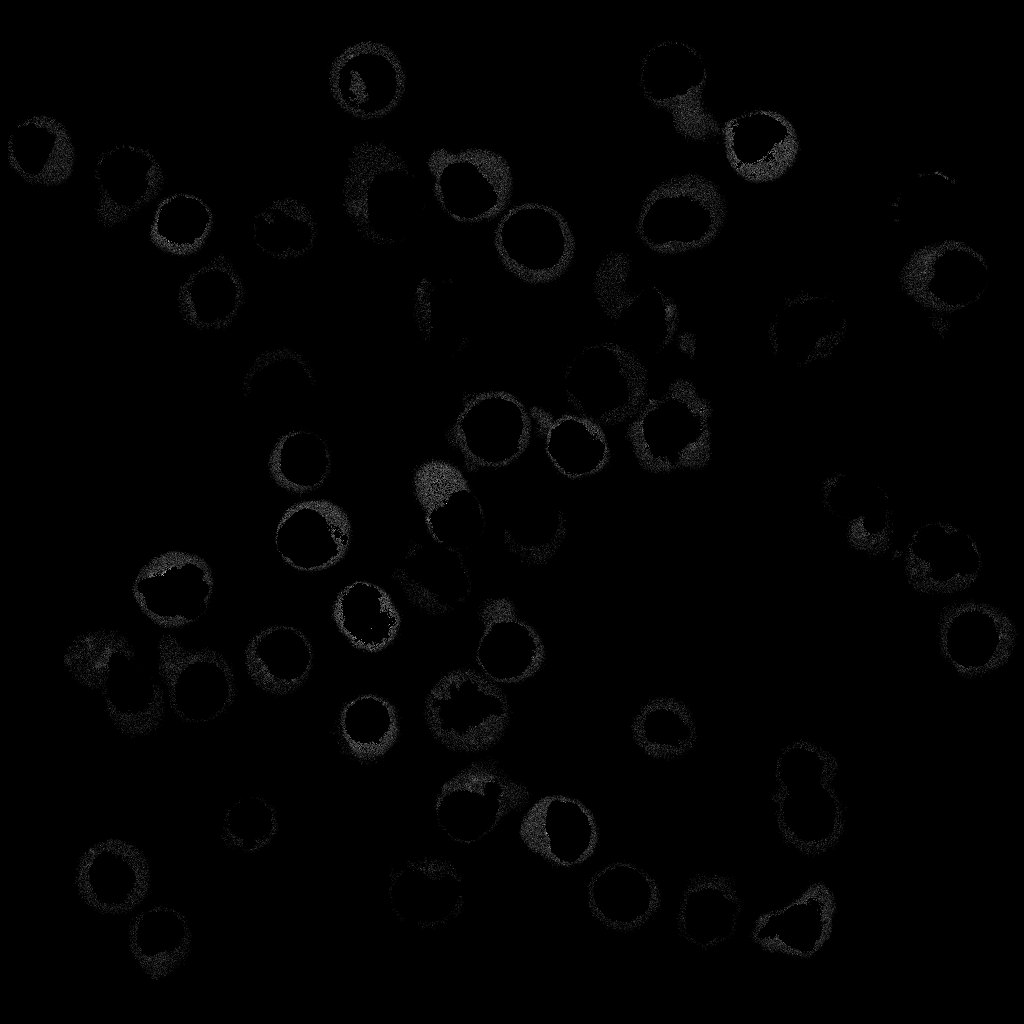

Supplement: Supplementary file 9 — Source data Fig. 7 [file 44318_2024_235_MOESM9_ESM.zip › Figure 7/7A/pH6_8-std-green.jpg]

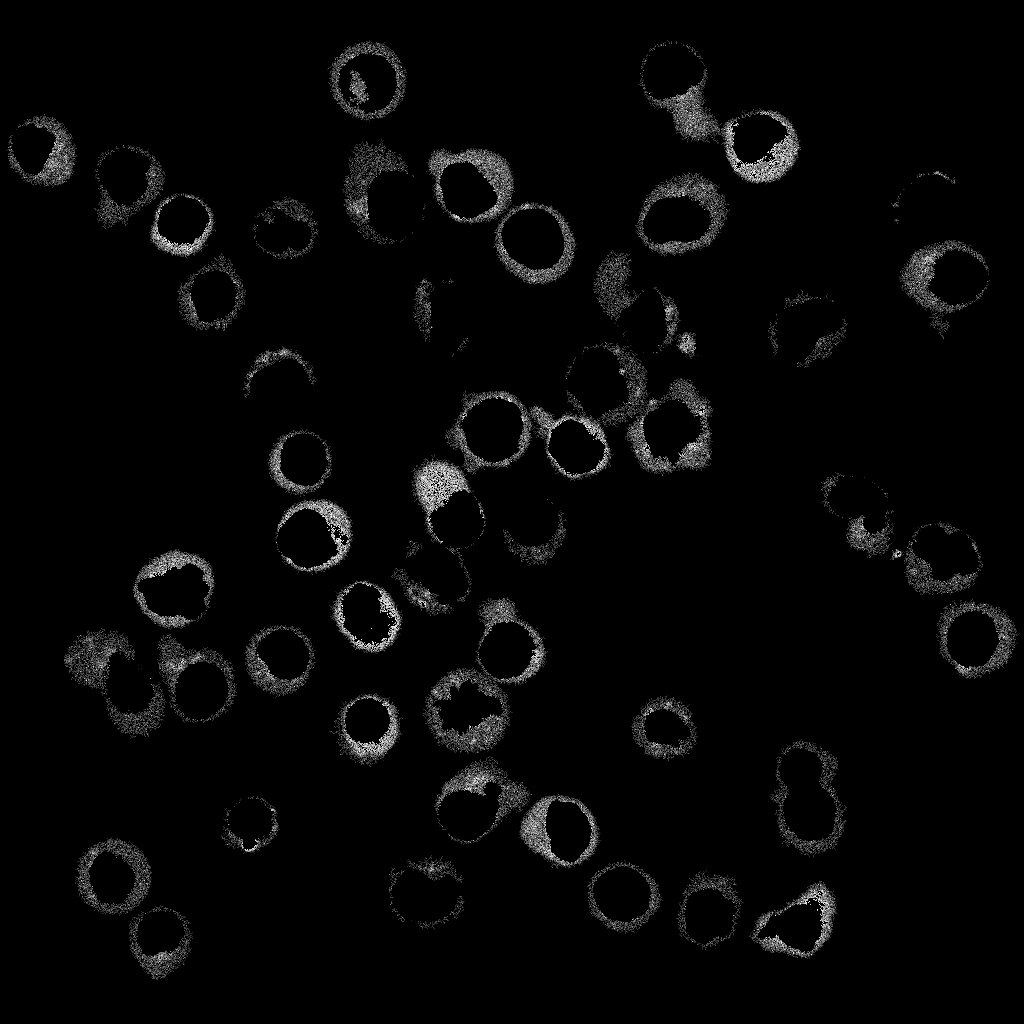

Supplement: Supplementary file 9 — Source data Fig. 7 [file 44318_2024_235_MOESM9_ESM.zip › Figure 7/7A/pH6_8-std-red.jpg]

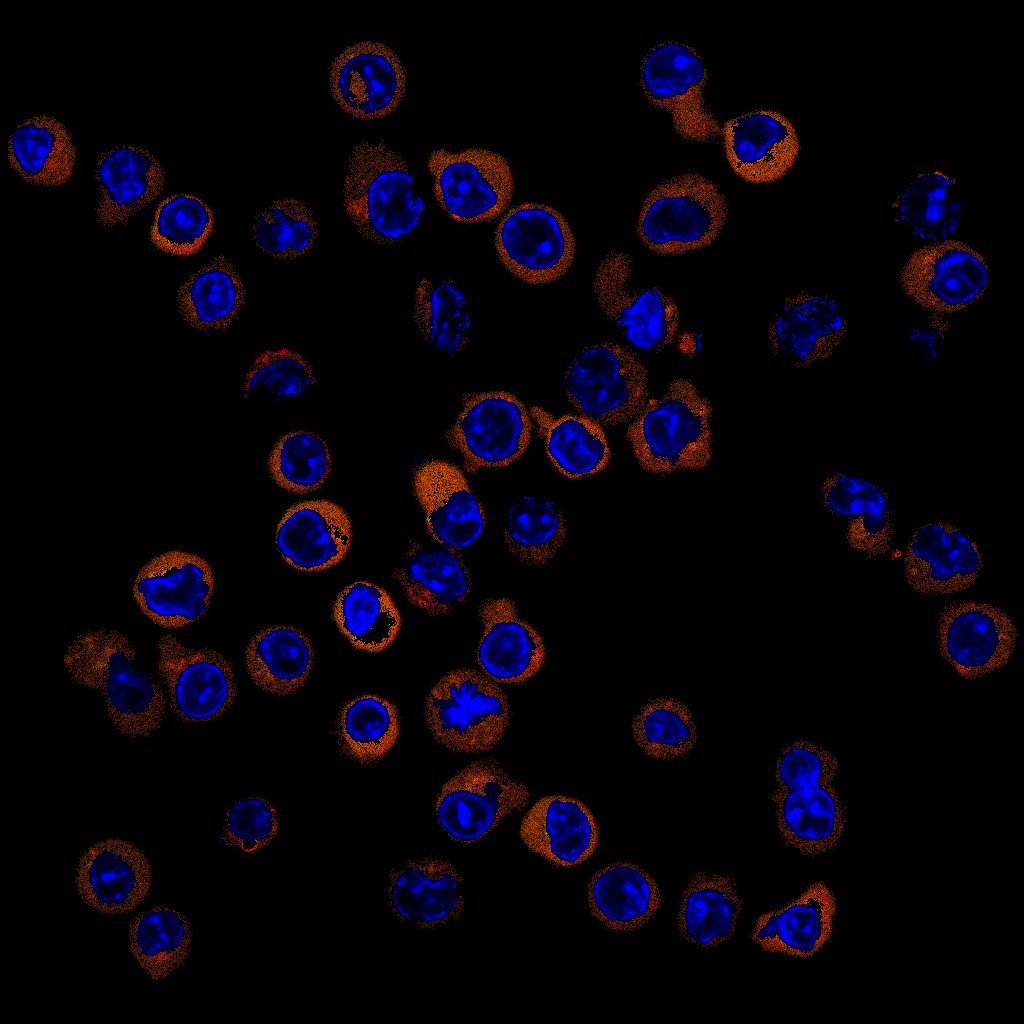

Supplement: Supplementary file 9 — Source data Fig. 7 [file 44318_2024_235_MOESM9_ESM.zip › Figure 7/7A/pH6_8-std.jpg]

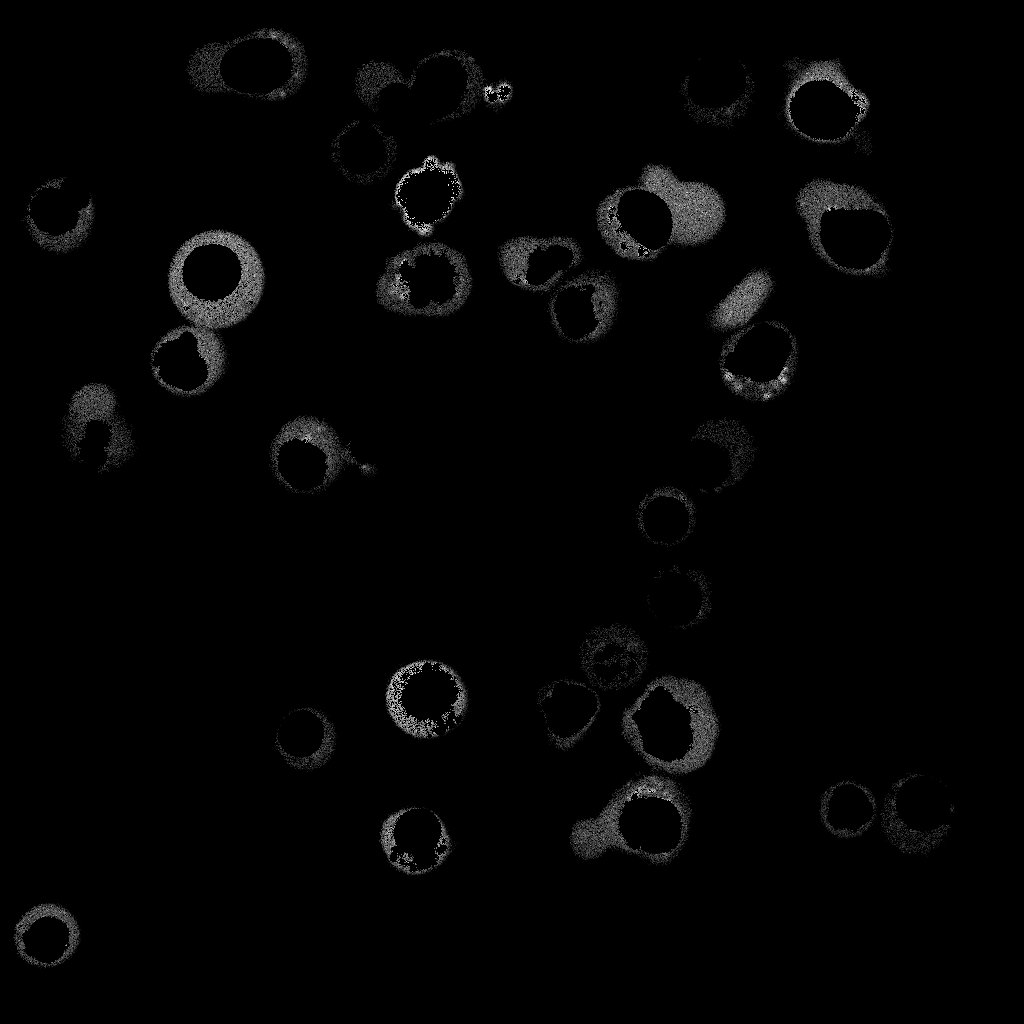

Supplement: Supplementary file 9 — Source data Fig. 7 [file 44318_2024_235_MOESM9_ESM.zip › Figure 7/7A/pH7_3-std-green.jpg]

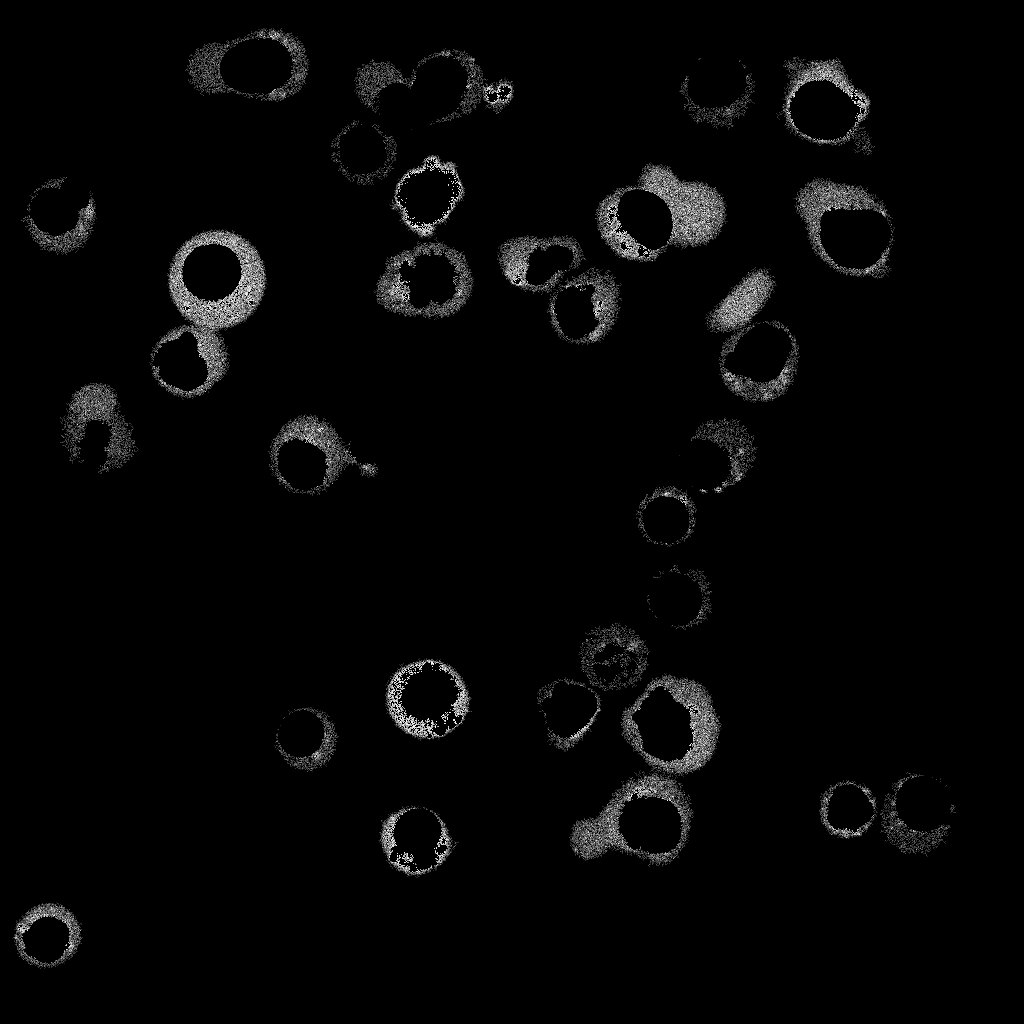

Supplement: Supplementary file 9 — Source data Fig. 7 [file 44318_2024_235_MOESM9_ESM.zip › Figure 7/7A/pH7_3-std-red.jpg]

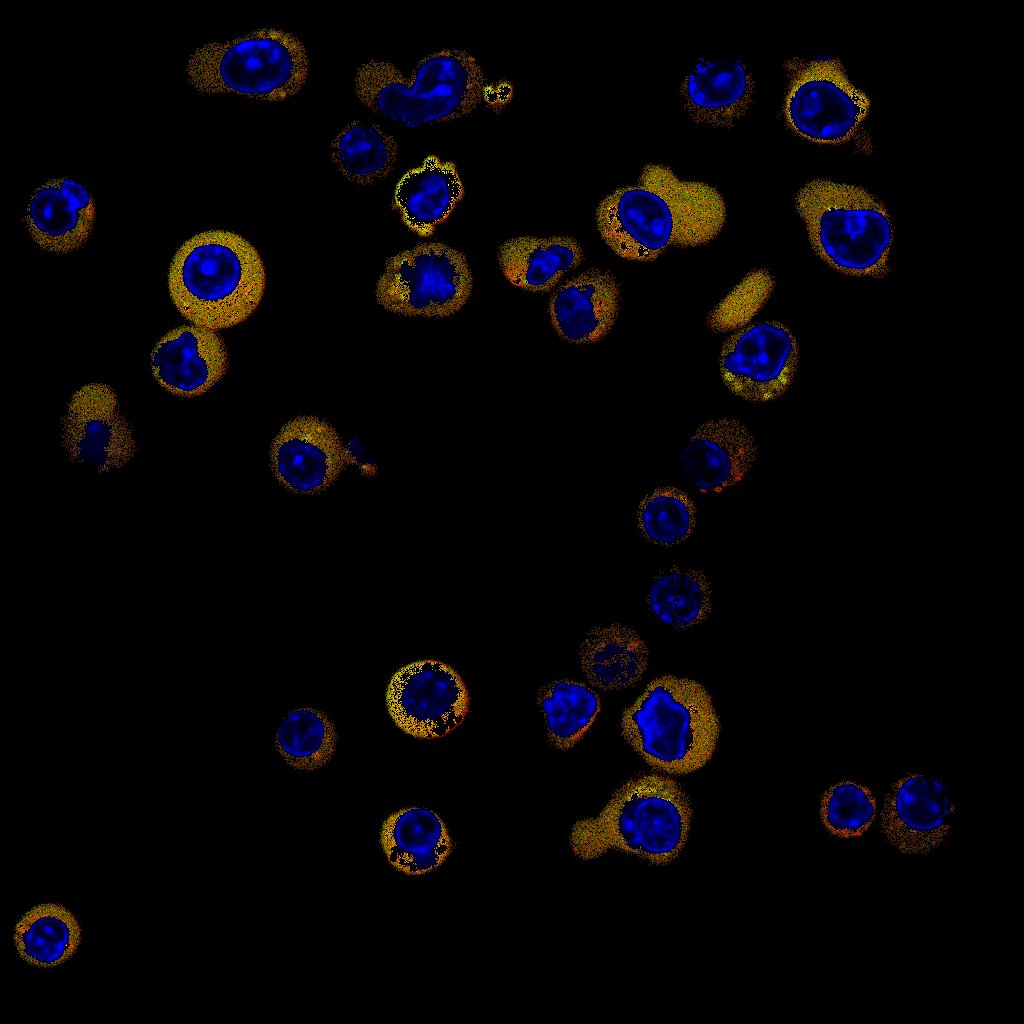

Supplement: Supplementary file 9 — Source data Fig. 7 [file 44318_2024_235_MOESM9_ESM.zip › Figure 7/7A/pH7_3-std.jpg]

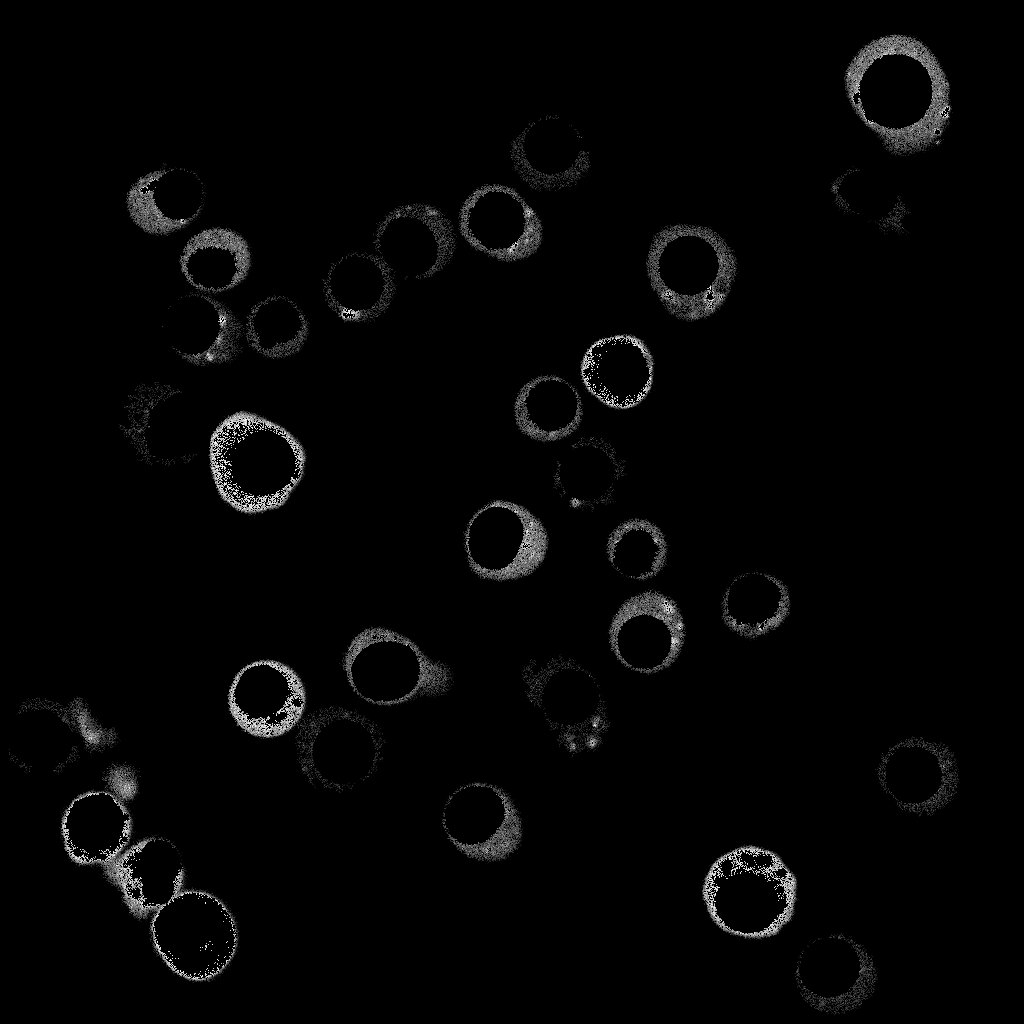

Supplement: Supplementary file 9 — Source data Fig. 7 [file 44318_2024_235_MOESM9_ESM.zip › Figure 7/7A/pH7_8-std-green.jpg]

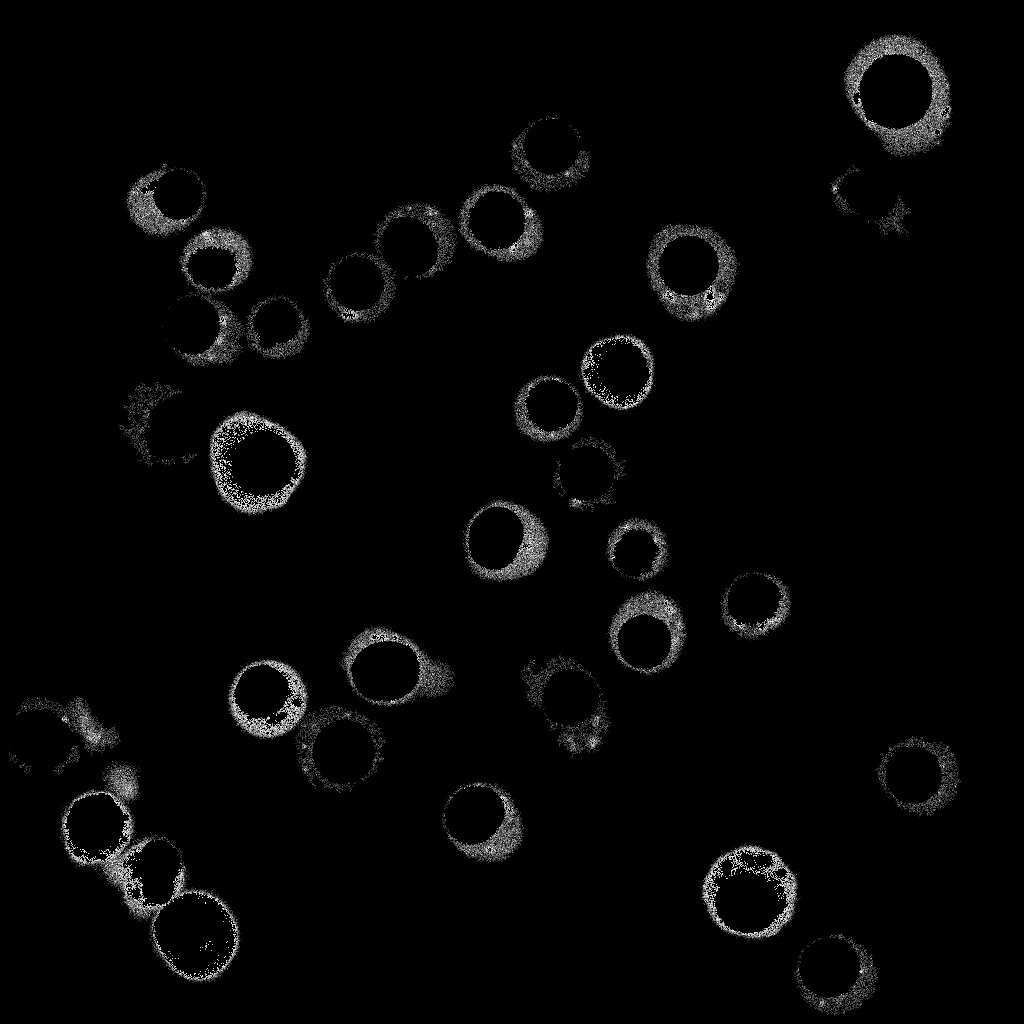

Supplement: Supplementary file 9 — Source data Fig. 7 [file 44318_2024_235_MOESM9_ESM.zip › Figure 7/7A/pH7_8-std-red.jpg]

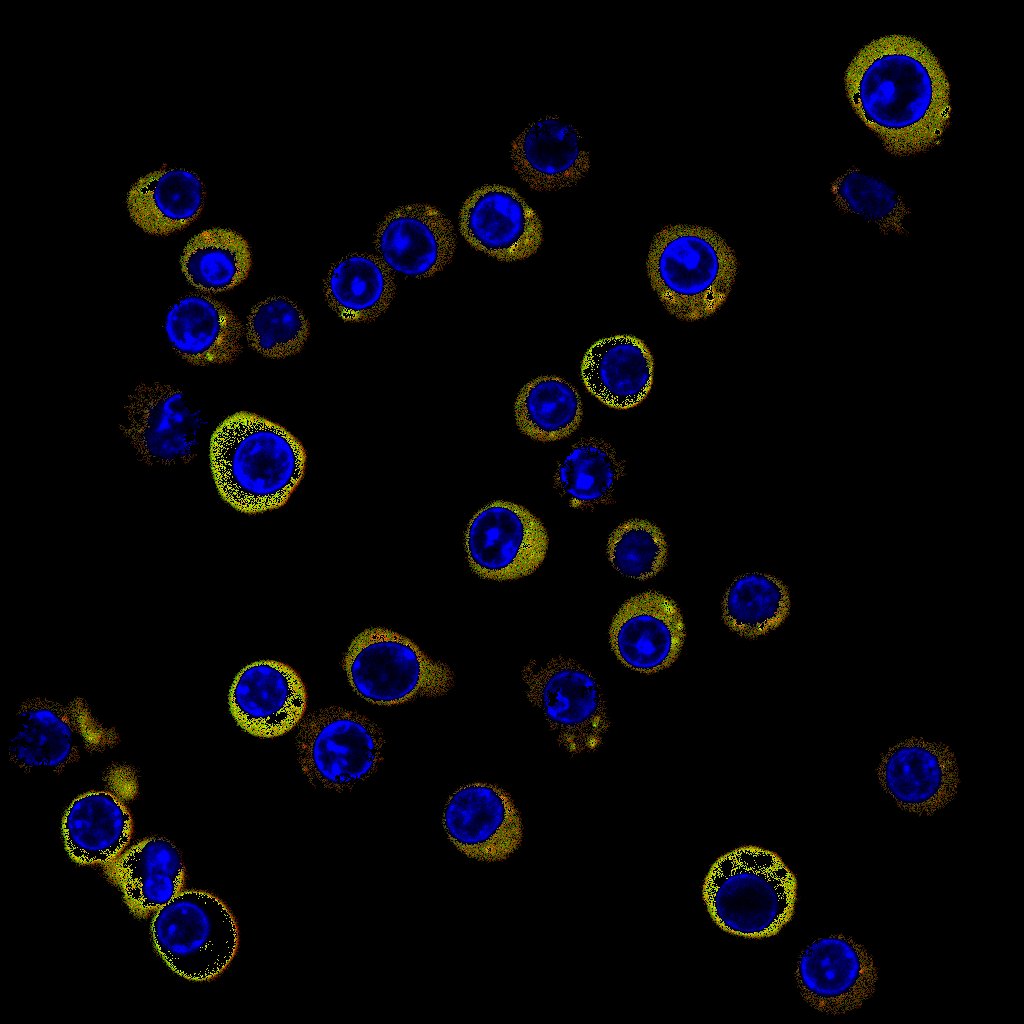

Supplement: Supplementary file 9 — Source data Fig. 7 [file 44318_2024_235_MOESM9_ESM.zip › Figure 7/7A/pH7_8-std.jpg]

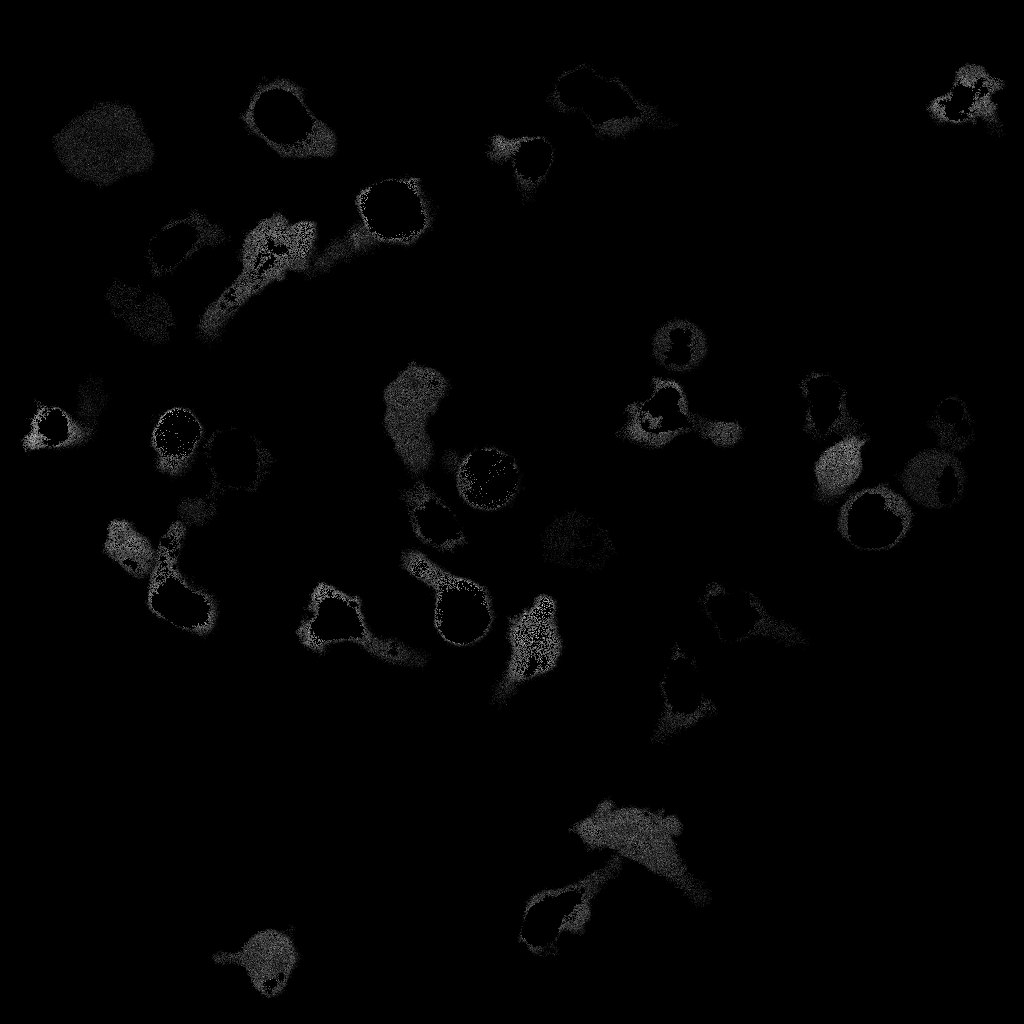

Supplement: Supplementary file 9 — Source data Fig. 7 [file 44318_2024_235_MOESM9_ESM.zip › Figure 7/7B/pH6_6-green.jpg]

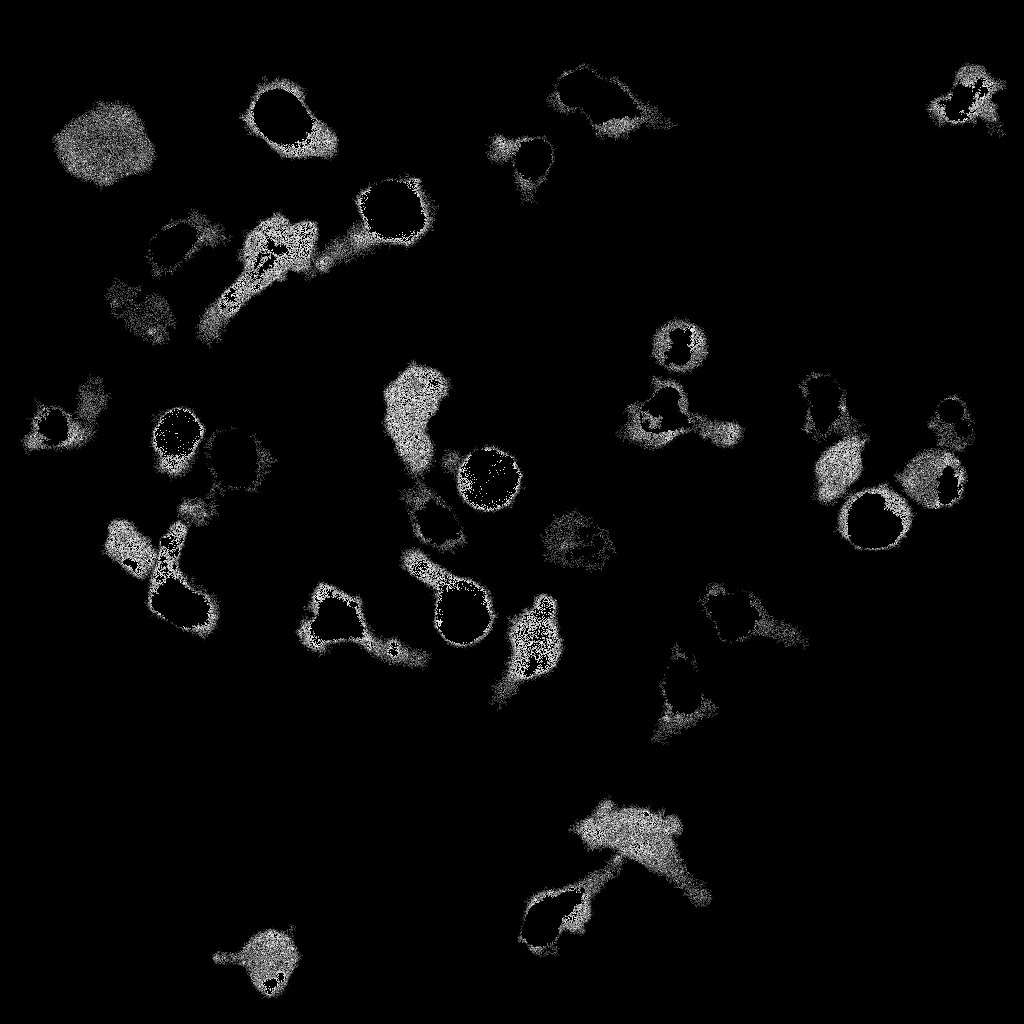

Supplement: Supplementary file 9 — Source data Fig. 7 [file 44318_2024_235_MOESM9_ESM.zip › Figure 7/7B/pH6_6-red.jpg]

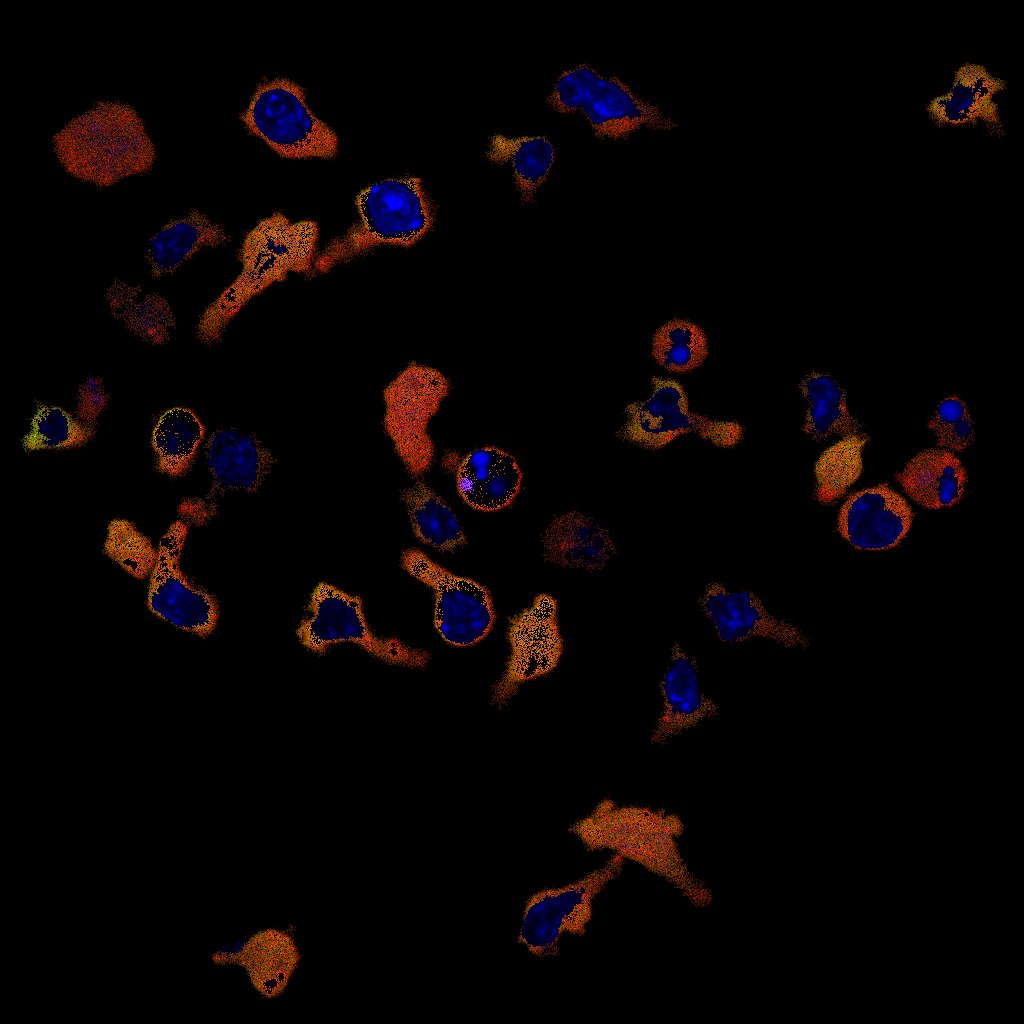

Supplement: Supplementary file 9 — Source data Fig. 7 [file 44318_2024_235_MOESM9_ESM.zip › Figure 7/7B/pH6_6.jpg]

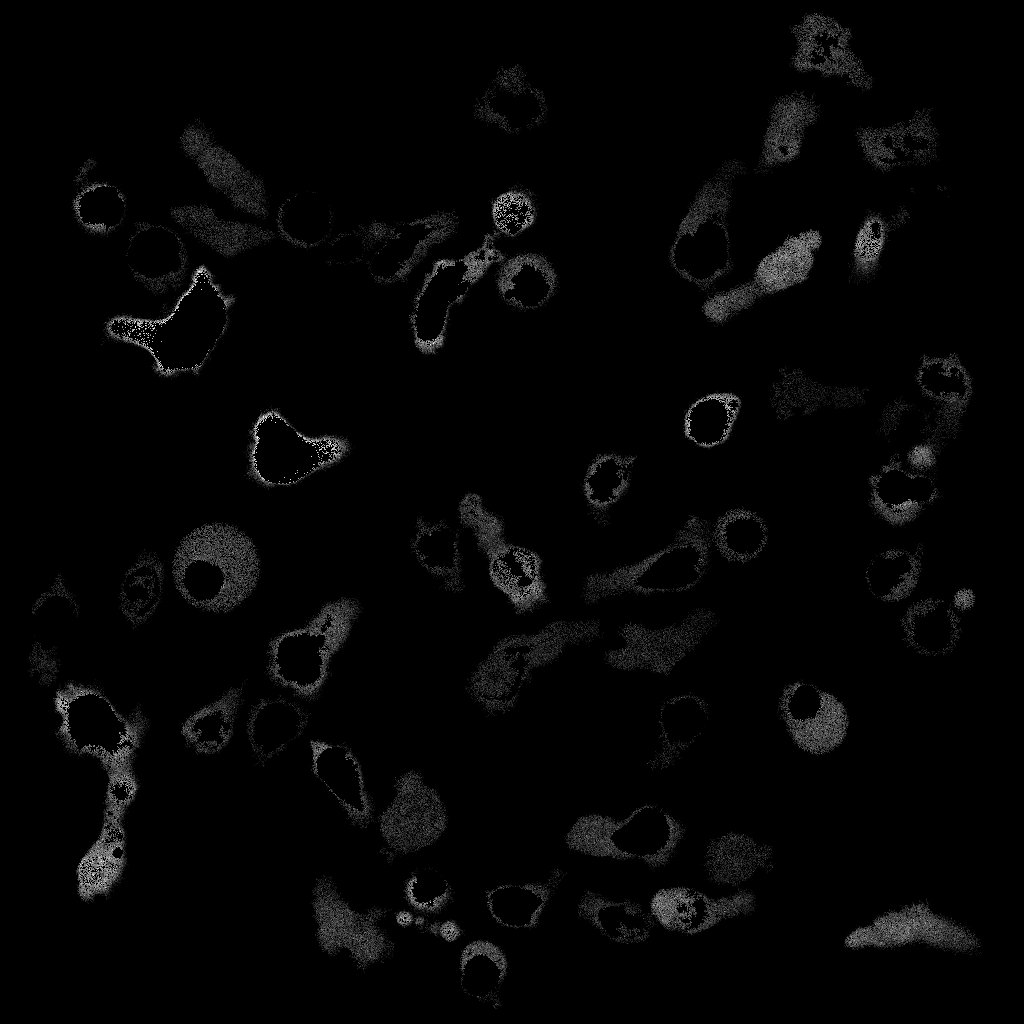

Supplement: Supplementary file 9 — Source data Fig. 7 [file 44318_2024_235_MOESM9_ESM.zip › Figure 7/7B/pH7_4-green.jpg]

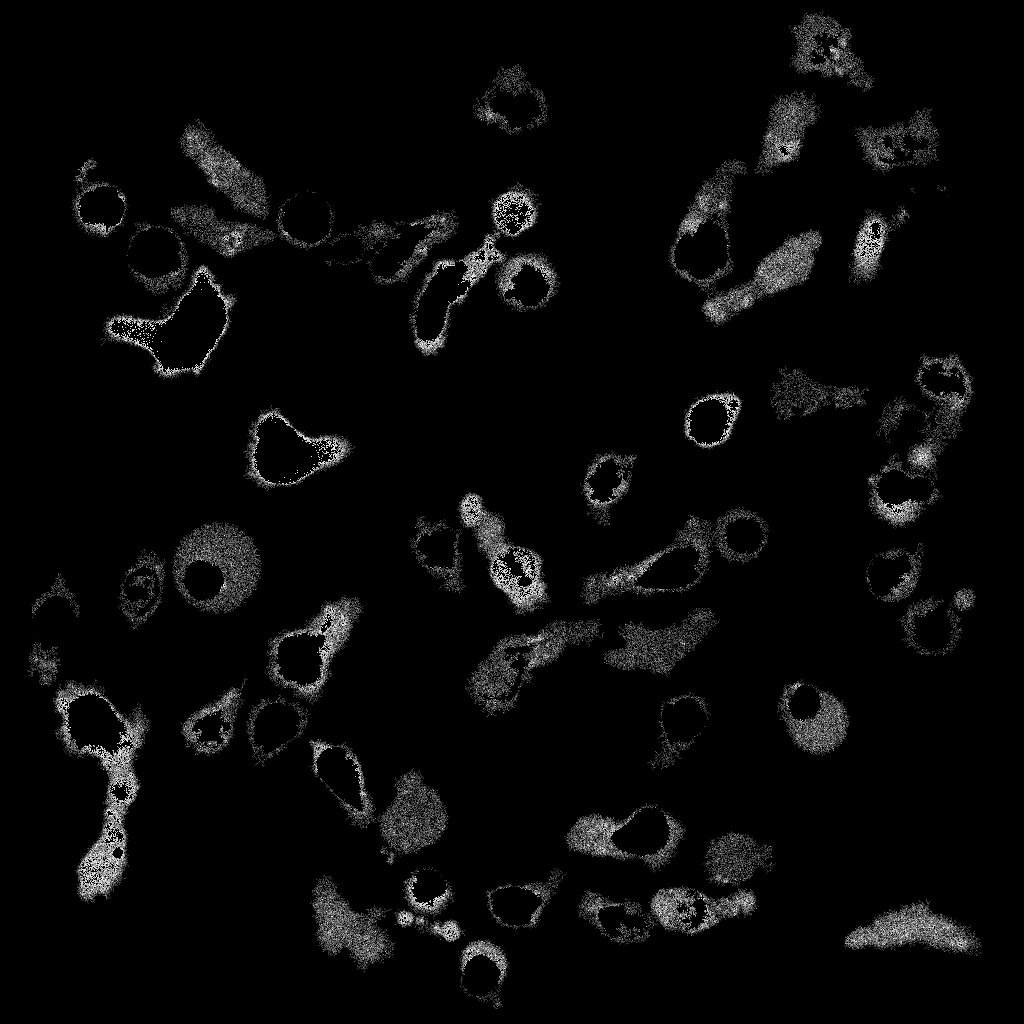

Supplement: Supplementary file 9 — Source data Fig. 7 [file 44318_2024_235_MOESM9_ESM.zip › Figure 7/7B/pH7_4-red.jpg]

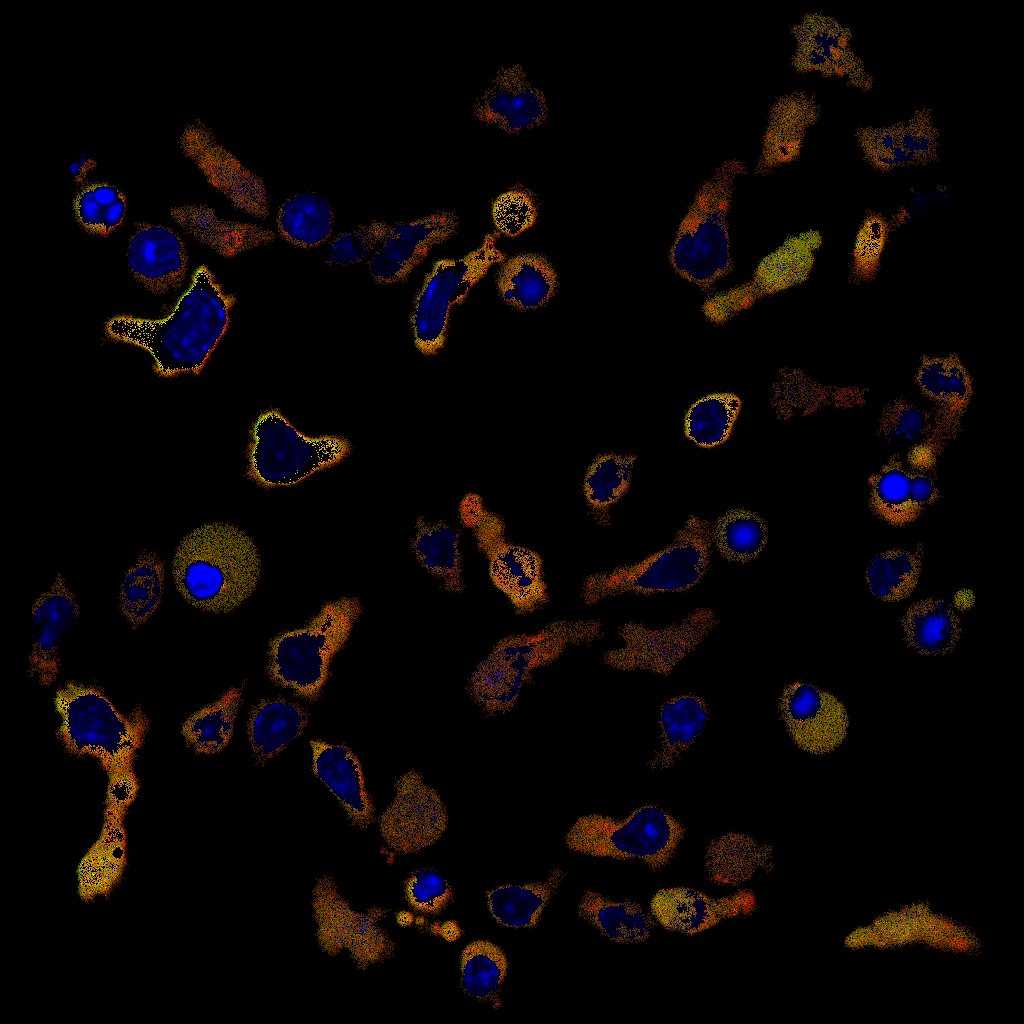

Supplement: Supplementary file 9 — Source data Fig. 7 [file 44318_2024_235_MOESM9_ESM.zip › Figure 7/7B/pH7_4.jpg]

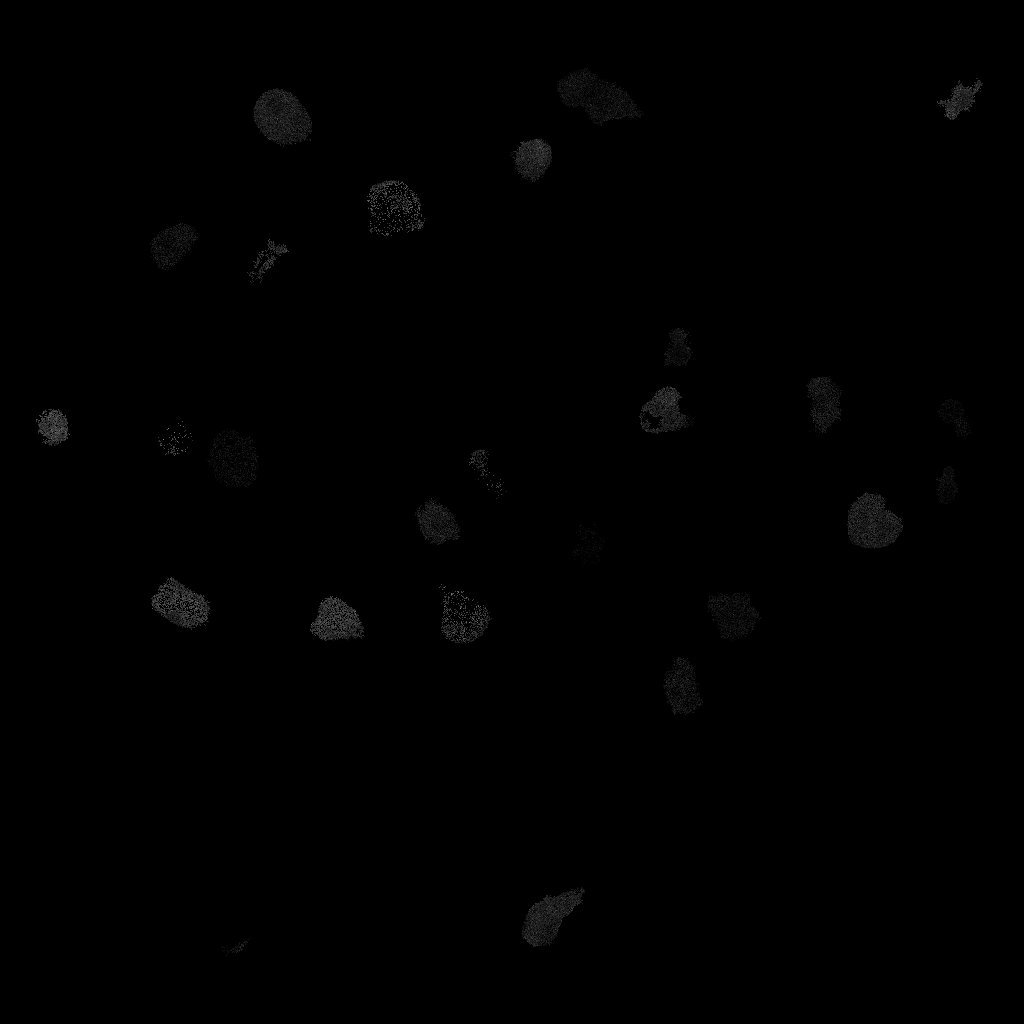

Supplement: Supplementary file 9 — Source data Fig. 7 [file 44318_2024_235_MOESM9_ESM.zip › Figure 7/7C/pH6_6-nucleus-green.jpg]

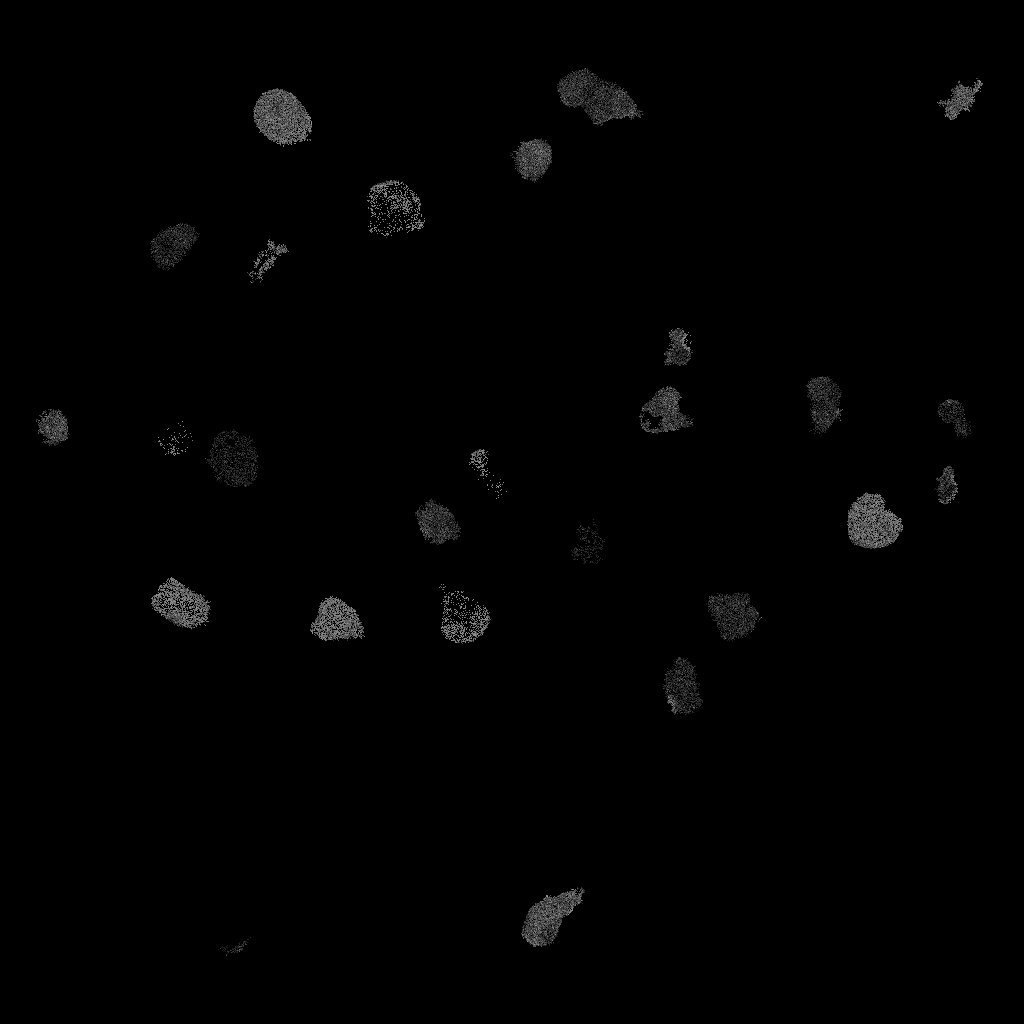

Supplement: Supplementary file 9 — Source data Fig. 7 [file 44318_2024_235_MOESM9_ESM.zip › Figure 7/7C/pH6_6-nucleus-red.jpg]

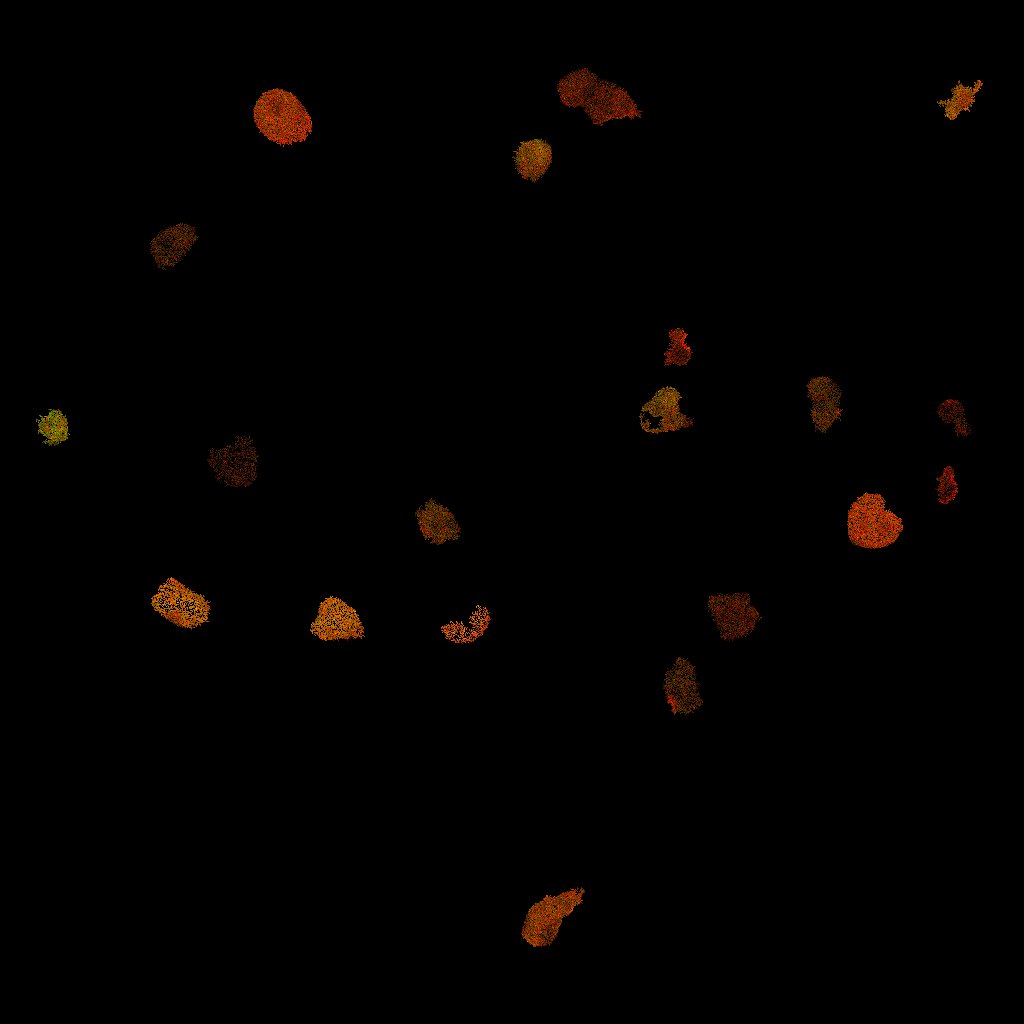

Supplement: Supplementary file 9 — Source data Fig. 7 [file 44318_2024_235_MOESM9_ESM.zip › Figure 7/7C/pH6_6-nucleus.jpg]

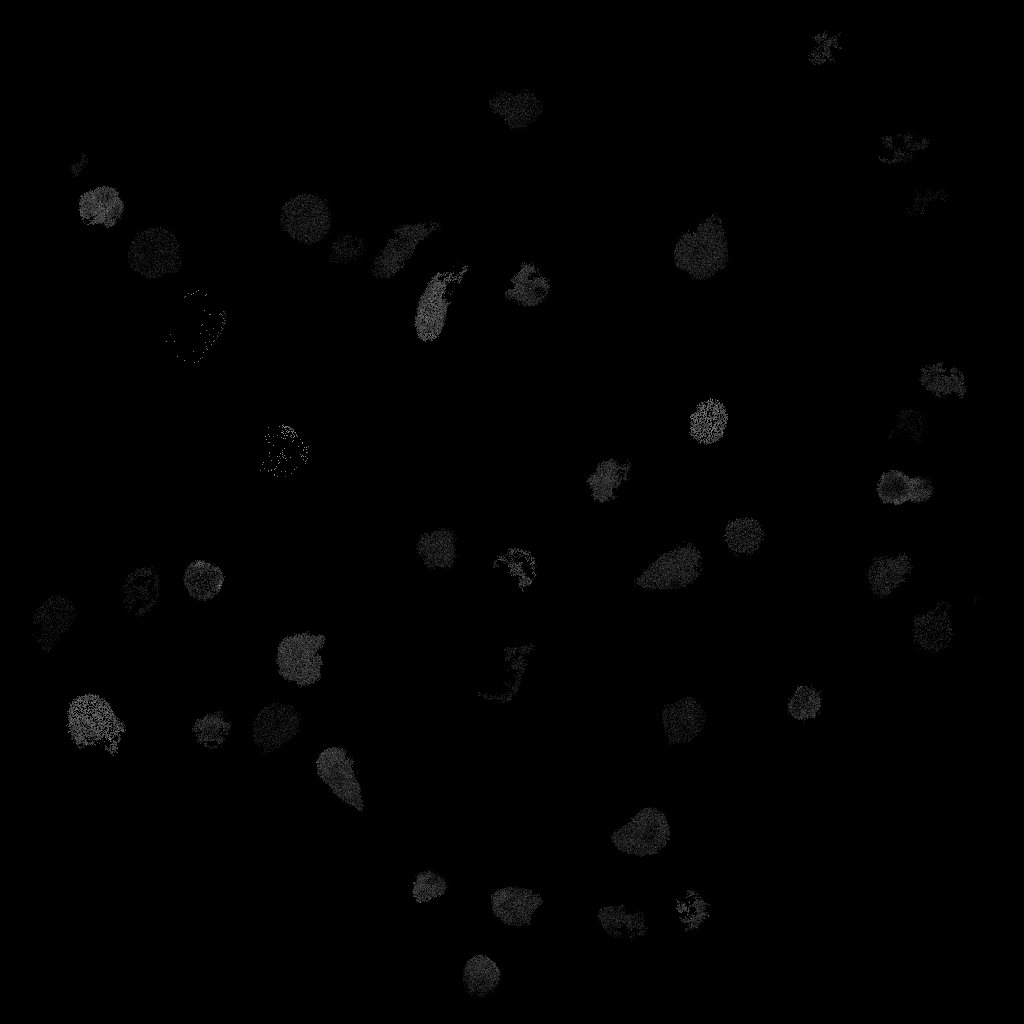

Supplement: Supplementary file 9 — Source data Fig. 7 [file 44318_2024_235_MOESM9_ESM.zip › Figure 7/7C/pH7_4-nucleus-green.jpg]

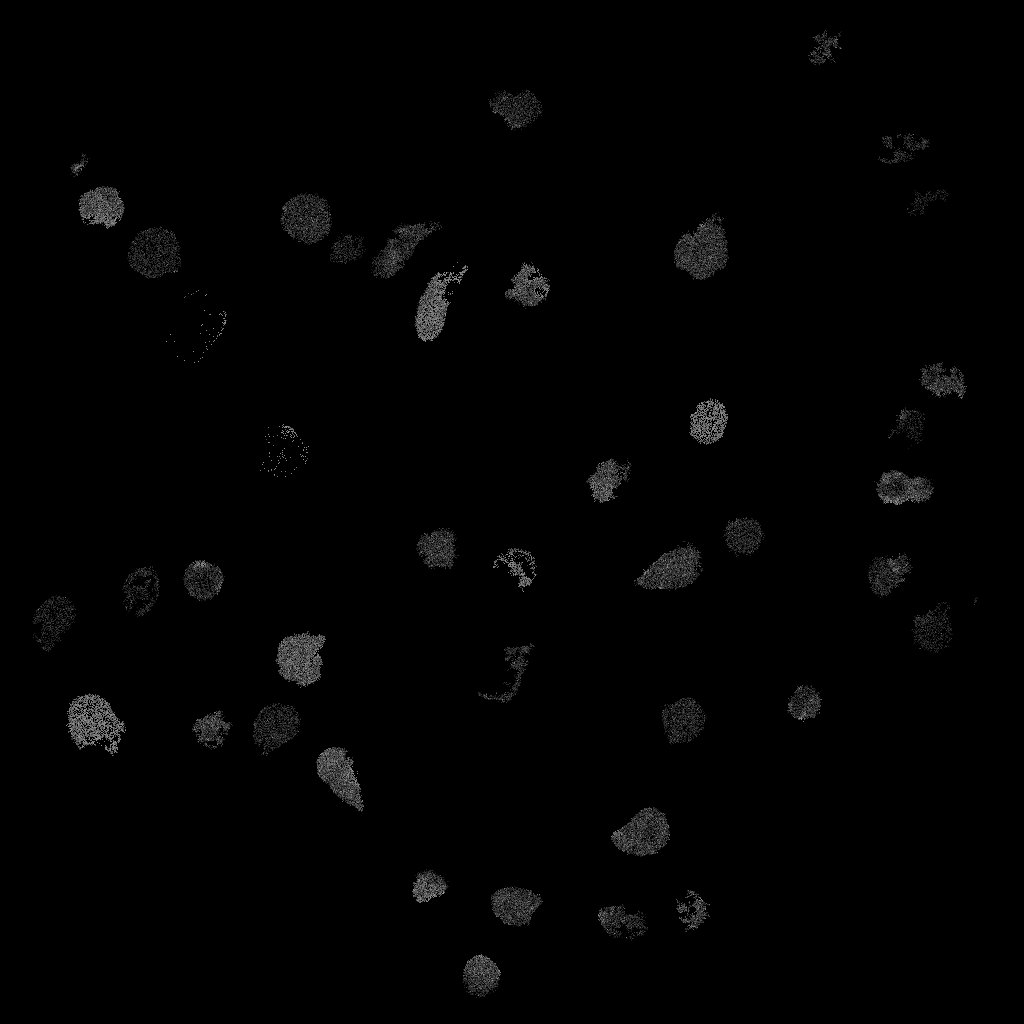

Supplement: Supplementary file 9 — Source data Fig. 7 [file 44318_2024_235_MOESM9_ESM.zip › Figure 7/7C/pH7_4-nucleus-red.jpg]

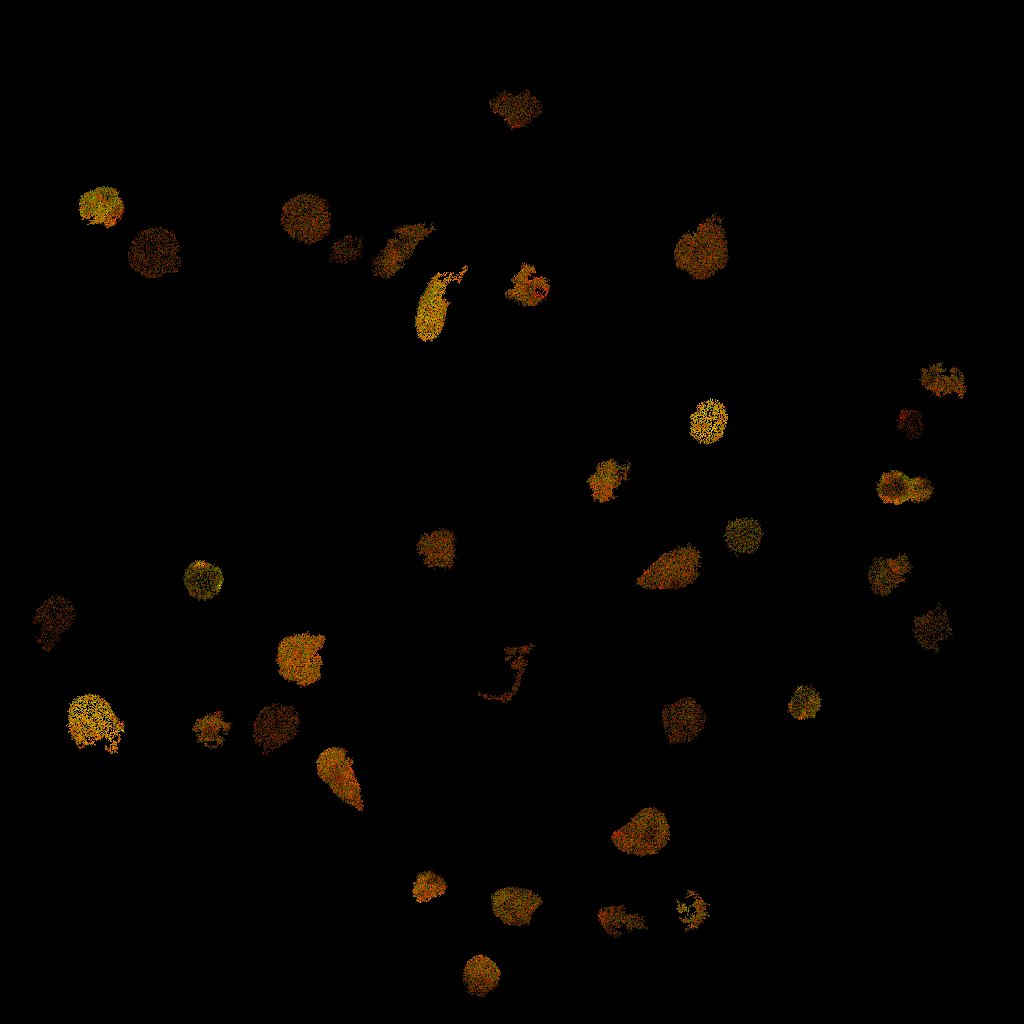

Supplement: Supplementary file 9 — Source data Fig. 7 [file 44318_2024_235_MOESM9_ESM.zip › Figure 7/7C/pH7_4-nucleus.jpg]

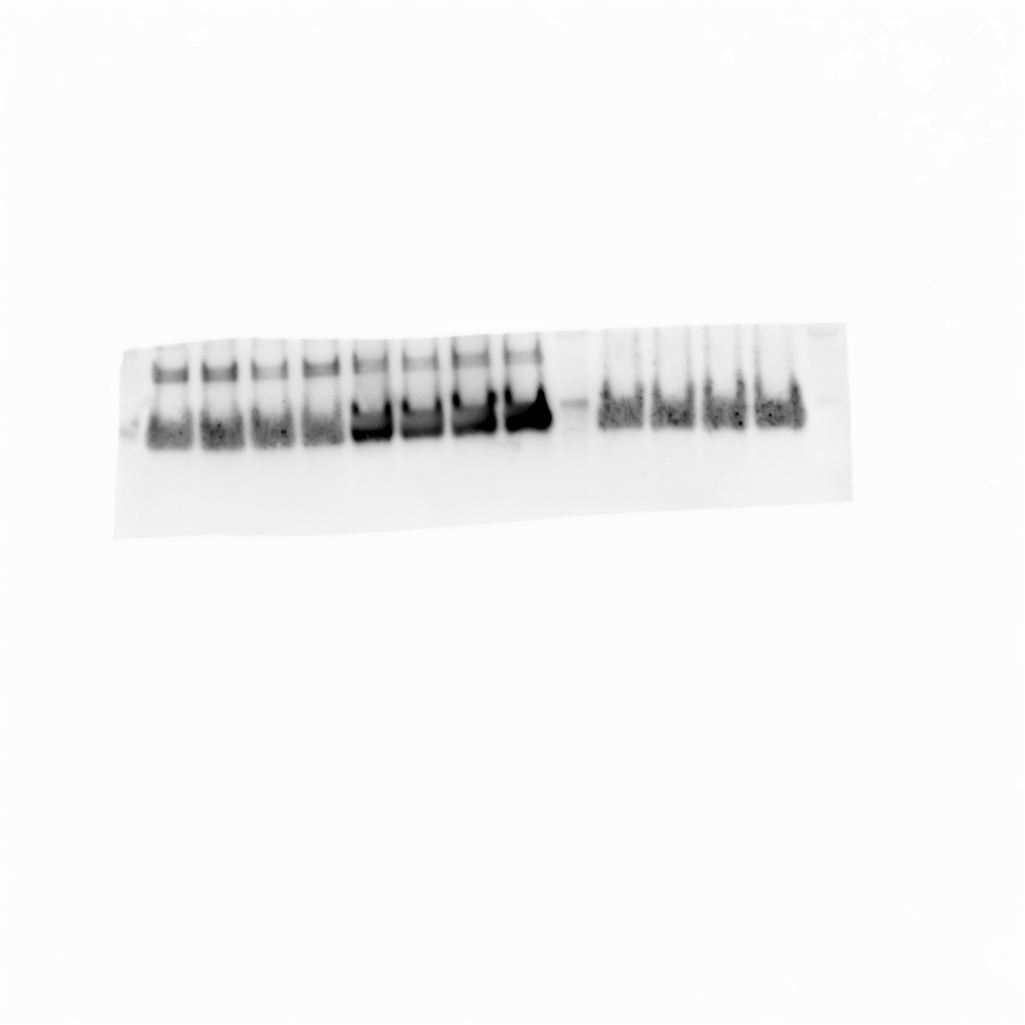

Supplement: Supplementary file 9 — Source data Fig. 7 [file 44318_2024_235_MOESM9_ESM.zip › Figure 7/7D/4ebp1-1s500.tif]

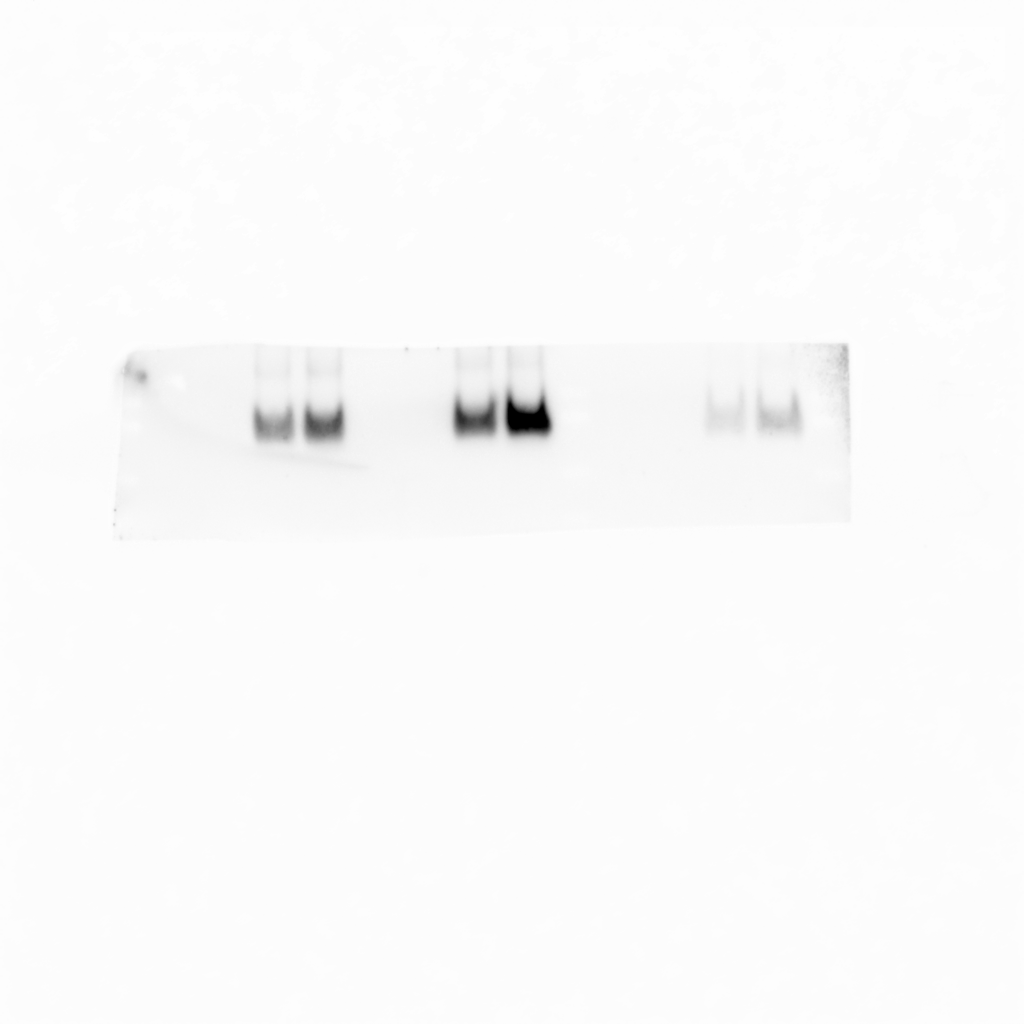

Supplement: Supplementary file 9 — Source data Fig. 7 [file 44318_2024_235_MOESM9_ESM.zip › Figure 7/7D/p4ebp1s65-40s.tif]

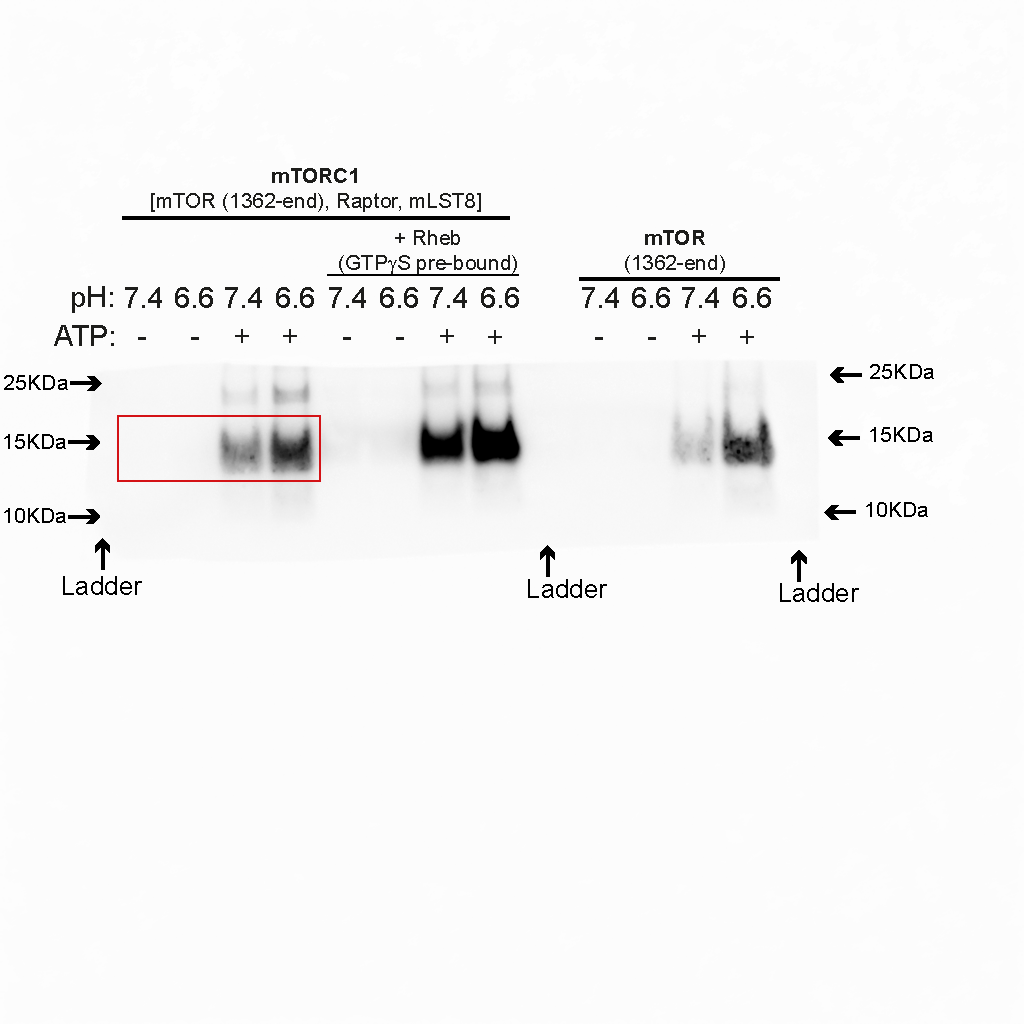

Supplement: Supplementary file 9 — Source data Fig. 7 [file 44318_2024_235_MOESM9_ESM.zip › Figure 7/7D/p4ebp1Thr-7s.tif]

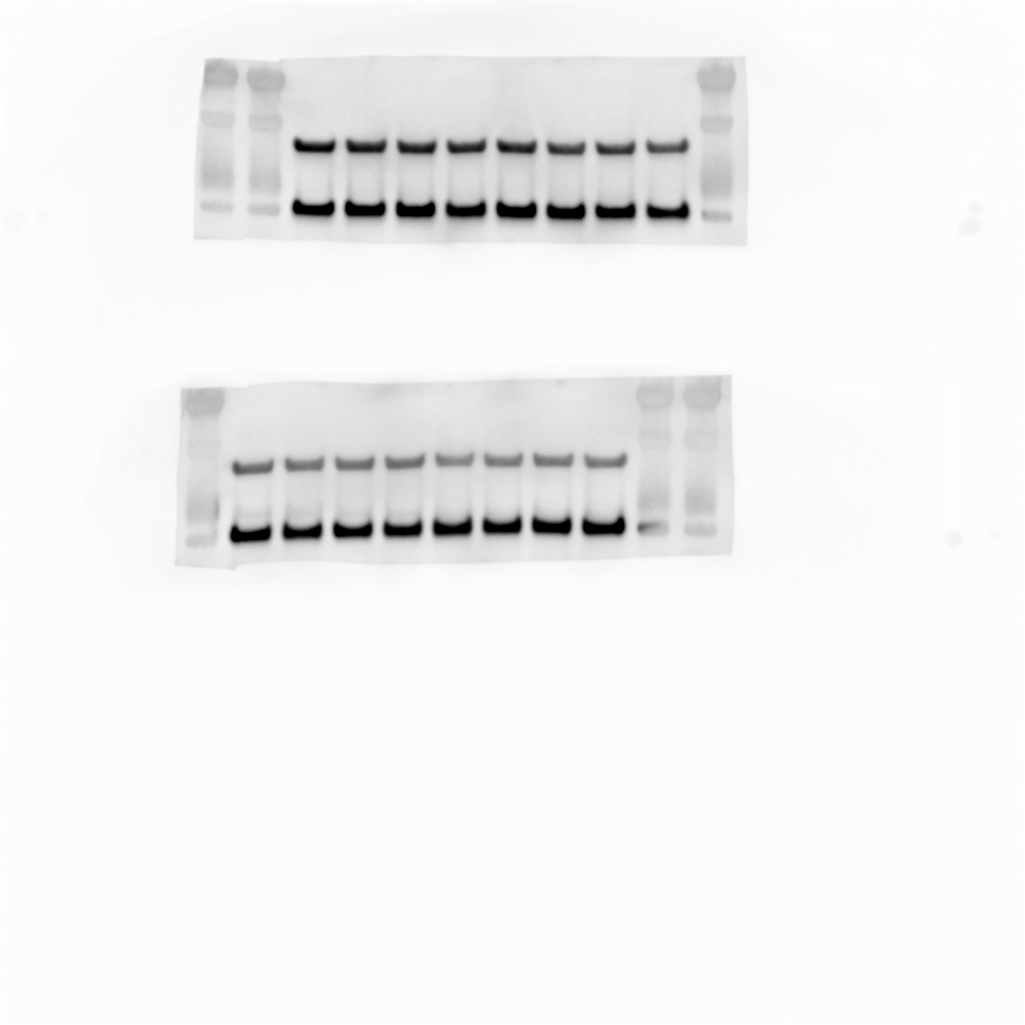

Supplement: Supplementary file 10 — Source data Fig. 8 [file 44318_2024_235_MOESM10_ESM.zip › Figure 8/8B/actin-45s.tif]

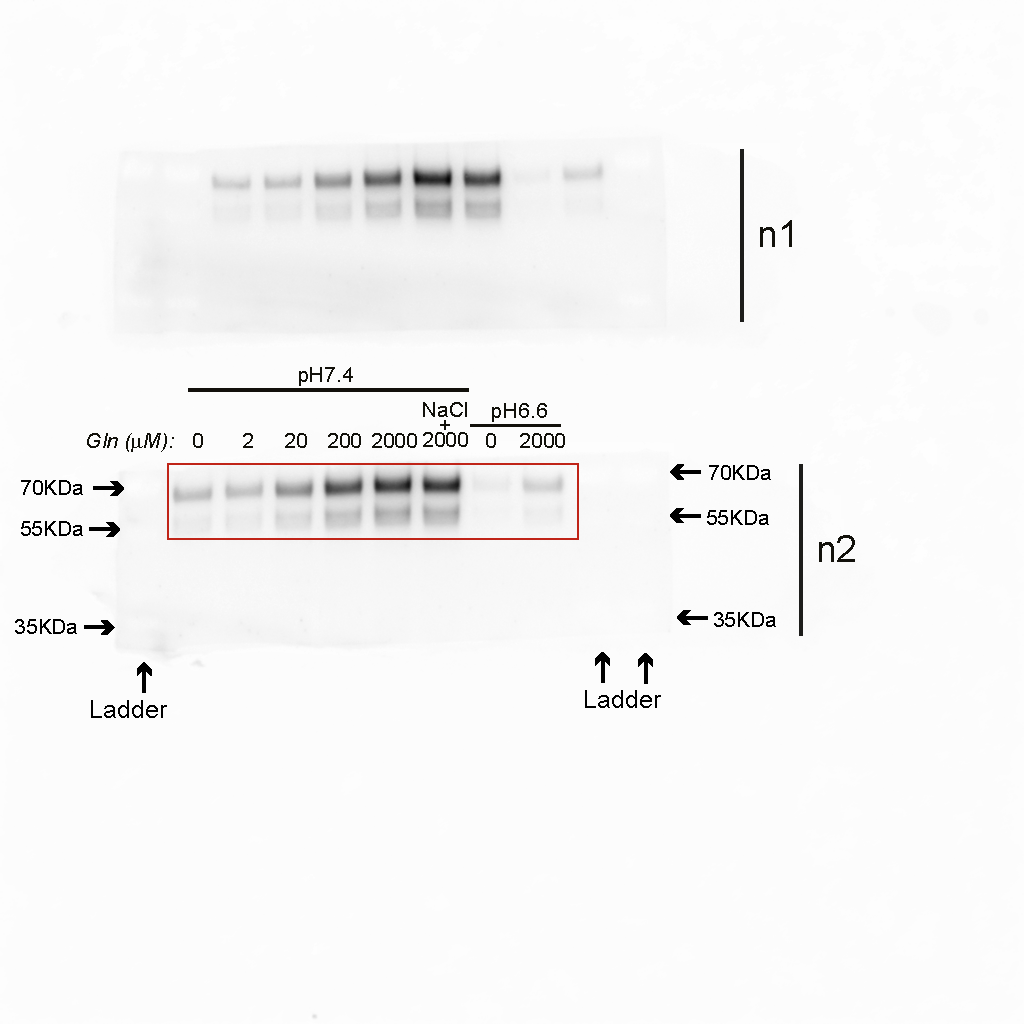

Supplement: Supplementary file 10 — Source data Fig. 8 [file 44318_2024_235_MOESM10_ESM.zip › Figure 8/8B/cmyc-1min.tif]

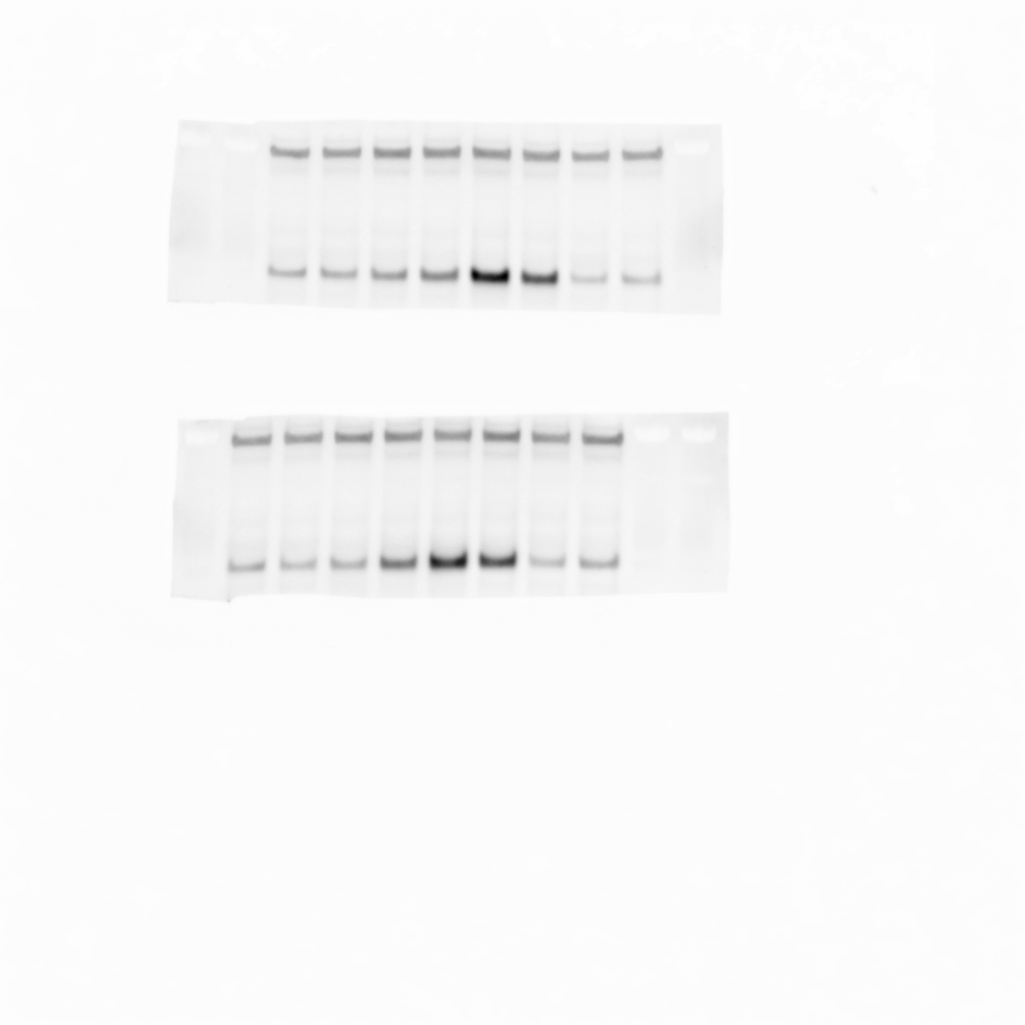

Supplement: Supplementary file 10 — Source data Fig. 8 [file 44318_2024_235_MOESM10_ESM.zip › Figure 8/8B/p70s6k-30s.tif]

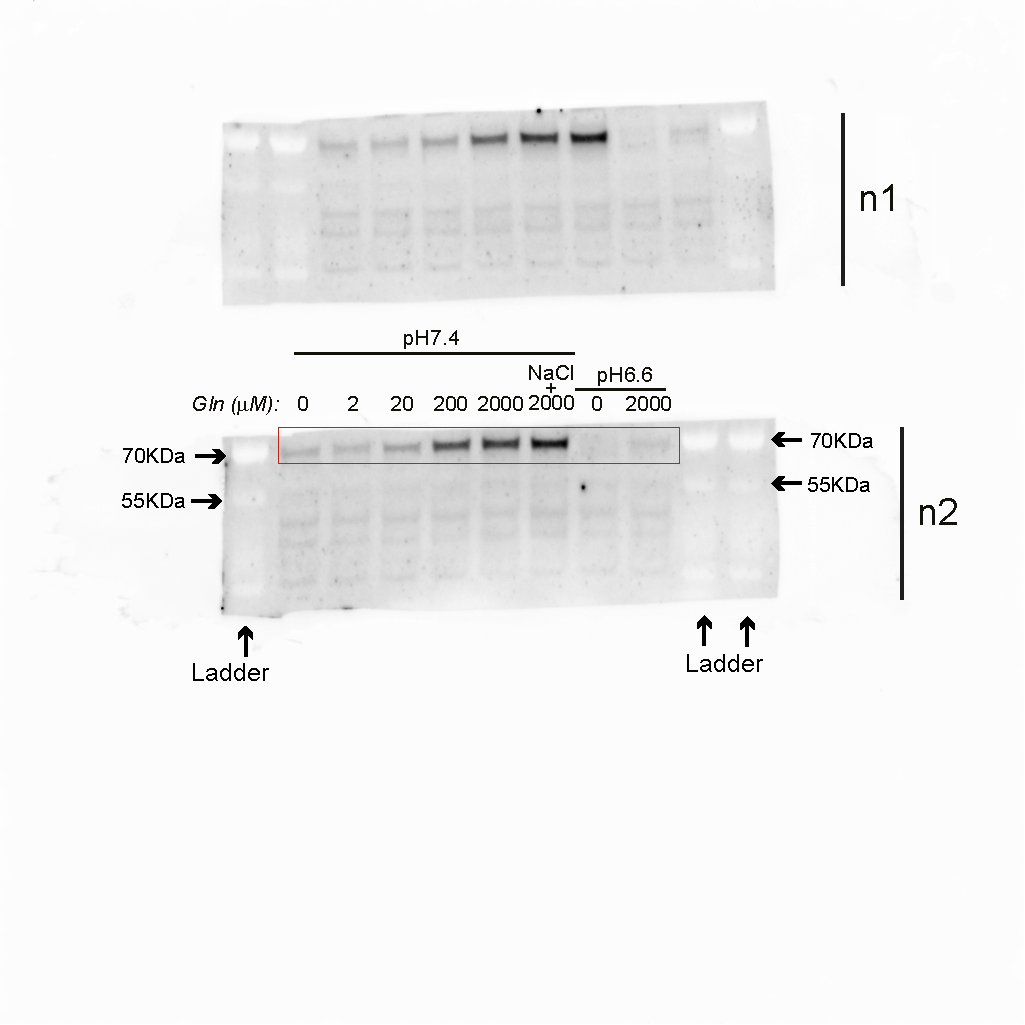

Supplement: Supplementary file 10 — Source data Fig. 8 [file 44318_2024_235_MOESM10_ESM.zip › Figure 8/8B/pp70s6k-6min30.tif]

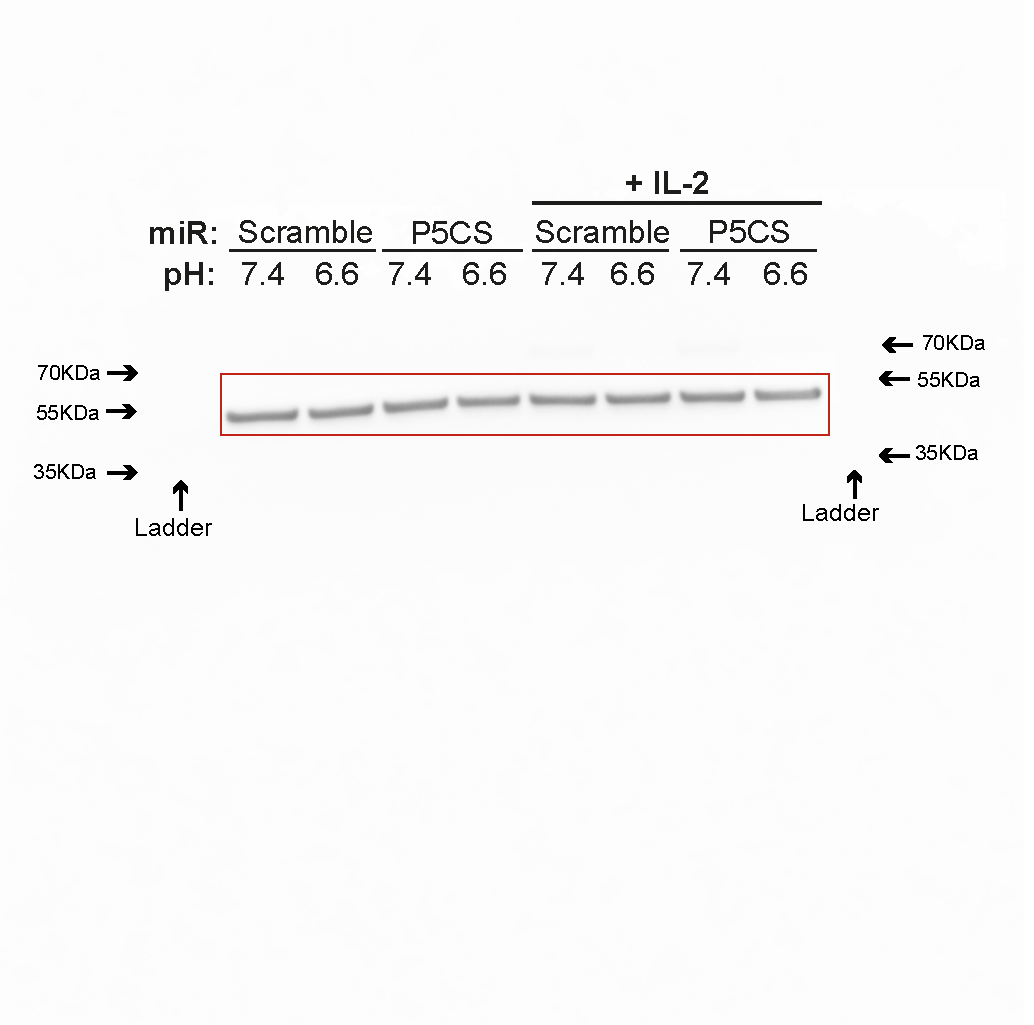

Supplement: Supplementary file 10 — Source data Fig. 8 [file 44318_2024_235_MOESM10_ESM.zip › Figure 8/8E/Actin-5s.tif]

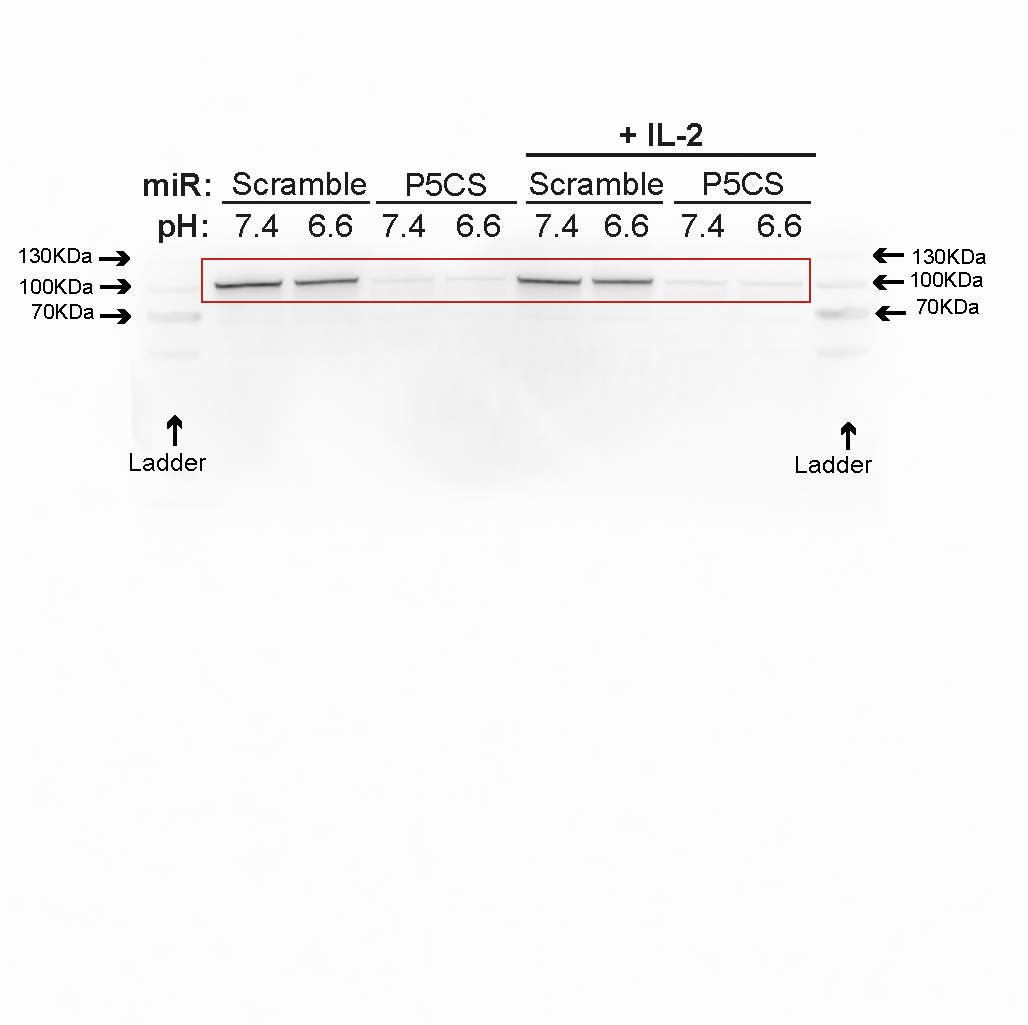

Supplement: Supplementary file 10 — Source data Fig. 8 [file 44318_2024_235_MOESM10_ESM.zip › Figure 8/8E/Aldh-3s.tif]
