# Supplementary material for: Acidity suppresses CD8 + T-cell function by perturbing IL-2, mTORC1, and c-Myc signaling
Source: EMBO J. 2024 Sep 16;43(21):8. doi: 10.1038/s44318-024-00235-w (PMC11535206; doi:10.1038/s44318-024-00235-w)
Supplement: Supplementary file 11 — Expanded View Figures [file 44318_2024_235_MOESM11_ESM.pdf]

Expanded View Figures

Figure EV1. Impact of acidity on CTL size, granularity and proliferation in response to anti-CD3 stimulation.

(**A**) Illustration of the main steps and functions of anti-tumor CD8 + T cell migrating to the tumor microenvironment. Previously differentiated anti-tumor CD8 + T cells (i.e. effector cytotoxic T lymphocytes; CTLs) encounter acidic conditions at the tumor site when they exert their activities. (**B**) Schematic of the in vitro methodology used to deconvolve the impact of pH on CTLs function in tumors. (**C**) Effector/memory phenotype of primed and expanded OT-I $CD8^+$ T cells used in this study. Naive OT-I $CD8^+$ T cells were activated for two days with pre-coated anti-CD3 and soluble anti-CD28 antibodies, and expanded for further ten days in the presence of murine IL-2. Dot plots show one representative experiment of background staining ("Isotype", top graph), and of CD44 CD62L staining (bottom graph) obtained by flow cytometry. Histograms show one representative experiment of background staining ("Isotype", black dashed line), and of Granzyme B staining (red solid line) obtained by flow cytometry. Almost 80% of cells are effectors ($CD44^+$ $CD62L^-$), while all express Granzyme B to relatively high levels. (**D**) Effector generation protocol gives efficient killing capacities of the resulting OT-I T cells. Varying numbers of OT-I CTLs were co-cultured with C1498 tumor cells pulsed (solid lines and symbols), or not (dashed lines and empty symbols), with 1 µM of antigen (minimal ovalbumin peptide epitope, SIINFEKL). Results show the mean percentage of tumor cell lysis ± SD of at least three (or two for 0.4 and 1.1 CTL:Tumor ratios) biological replicates from at least two independent experiments. (**E**) Impact of pH on CTL proliferation following re-activation with increasing doses of anti-CD3. OT-I CTLs were cultured for three days in the presence of an agonistic anti-CD3 antibody (pre-coated plates). Line graph show the estimated division number ± SEM of at least three biological replicates from at least two independent experiments. (**F**) Low pH prevents the increase of CTL size and granularity following anti-CD3 re-activation. OT-I CTLs were cultured in the presence of an agonistic anti-CD3 antibody (1 µg/mL pre-coated plates). Bar graph shows the mean cell size or granularity ± SEM of at least four biological replicates from at least two independent experiments. Density plots shows one representative experiment obtained by flow cytometry one day post re-stimulation. ns: not statistically significant, $^{****}P < 0.0001$. Cell size: $^{***}P = 0.0008$. Granularity: $^{**}P = 0.0018$, $^{***}P = 0.0002$ (one-way repeated measures ANOVA, Tukey post-hoc test). (**G**) Cartoon depicting the two main steps involved in cytokine secretion and proliferation following anti-CD3 re-activation of CTLs. The TCR/CD3-independent signaling allowing CTL proliferation is mostly mediated by IL-2/IL-2R signaling. (**H**) Design of the experiments in order to analyze at which step pH impacts cytokine secretion and proliferation during anti-CD3-mediated CTL re-activation. pH was neutralized or acidified at step 1, or 2, in order to know whether cytokine secretion or proliferation can be restored.

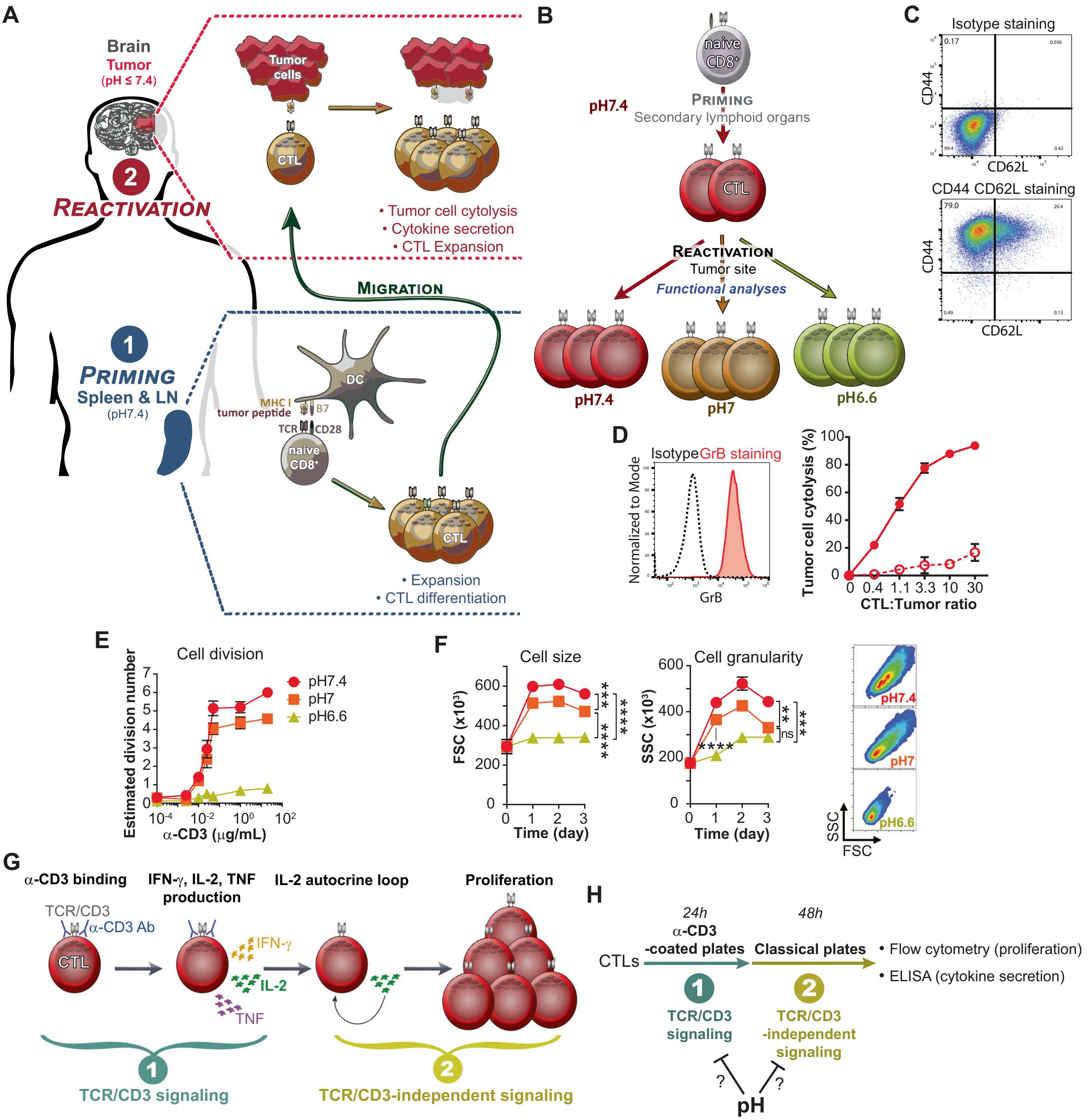
A
Brain
Tumor
(pH ≤ 7.4)
2
REACTIVATION
Tumor cells
CTL
• Tumor cell cytolysis
• Cytokine secretion
• CTL Expansion
MIGRATION
1
PRIMING
Spleen & LN
(pH7.4)
DC
MHC I
tumor peptide
B7
TCR
CD28
naive CD8+
CTL
• Expansion
• CTL differentiation
B
naive CD8+
pH7.4
PRIMING
Secondary lymphoid organs
CTL
REACTIVATION
Tumor site
Functional analyses
pH7.4
pH7
pH6.6
C
Isotype staining
CD44
CD62L
CD44 CD62L staining
CD44
CD62L
D
Isotype
GrB staining
Normalized to Mode
GrB
Tumor cell cytolysis (%)
CTL:Tumor ratio
E
Cell division
Estimated division number
pH7.4
pH7
pH6.6
α-CD3 (µg/mL)
F
Cell size
FSC (x10³)
Time (day)
Cell granularity
SSC (x10³)
ns
Time (day)
pH7.4
pH7
pH6.6
SSC
FSC
G
α-CD3 binding
IFN-γ, IL-2, TNF production
IL-2 autocrine loop
Proliferation
TCR/CD3
α-CD3 Ab
CTL
IFN-γ
IL-2
TNF
1
TCR/CD3 signaling
2
TCR/CD3-independent signaling
H
24h
α-CD3 -coated plates
48h
Classical plates
CTLs
• Flow cytometry (proliferation)
• ELISA (cytokine secretion)
1
TCR/CD3 signaling
2
TCR/CD3 -independent signaling
?
?
pH

 © The Author(s)

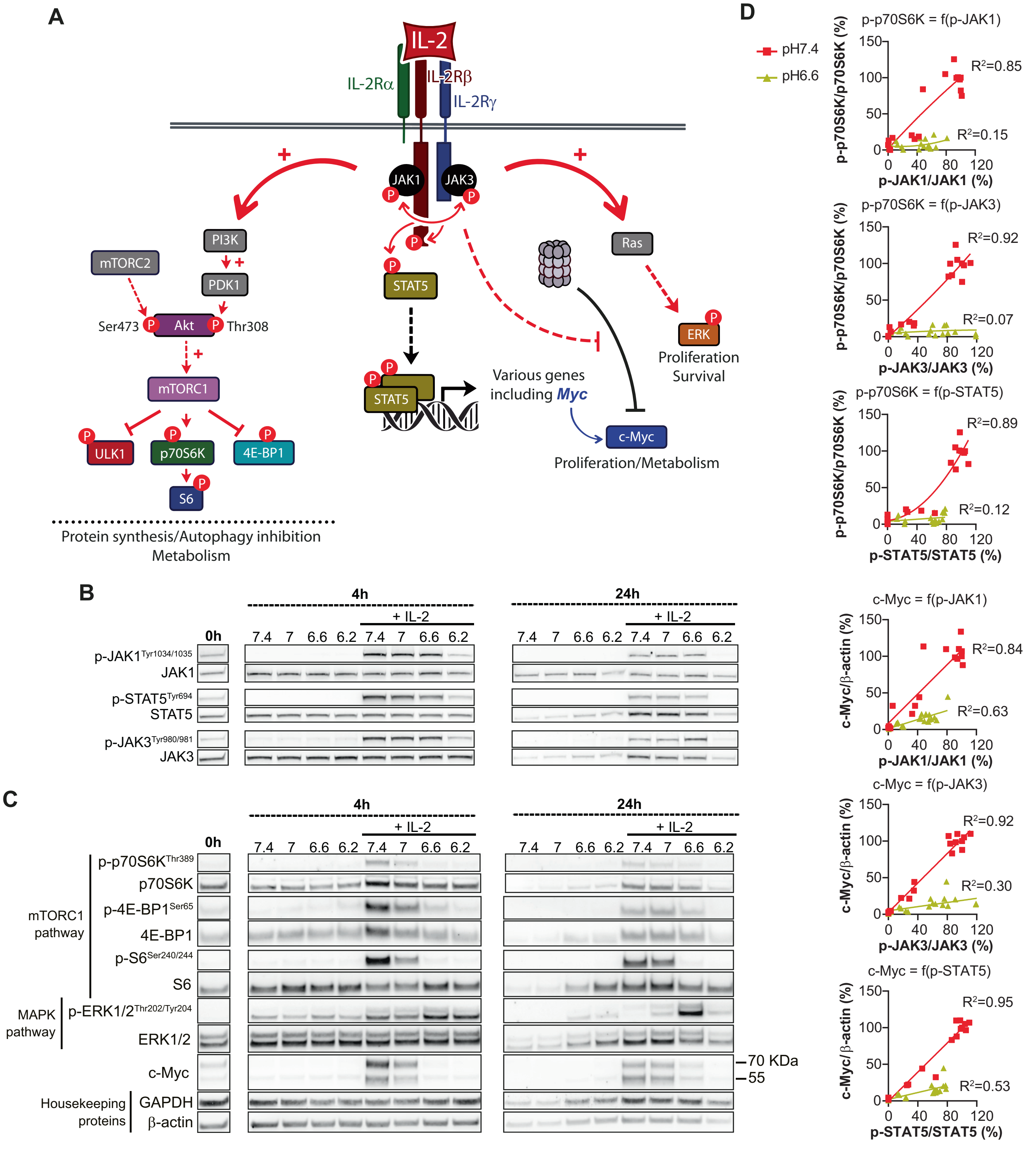

Figure EV2. Acidity lowers IL-2R signaling.

(**A**) Simplified scheme of the IL-2R signaling. (**B**) IL-2R signaling is disturbed at lower pH. OT-I CTLs were cultured at various pH in the presence, or the absence of exogenous murine IL-2 for 4 or 24 h. One representative western blot is shown. (**C**) Low pH disturbs IL-2 –induced mTORC1 pathway and c-Myc levels. The methodology is the same as in (**B**). One representative western blot is shown. (**D**) Correlation between the activation of first signaling transducers and mTORC1 pathway targets, or c-Myc levels. Results show individual values obtained from the experiments displayed in Fig. 3C. Corresponding correlation curves together with associated R^2 are shown.

© The Author(s)

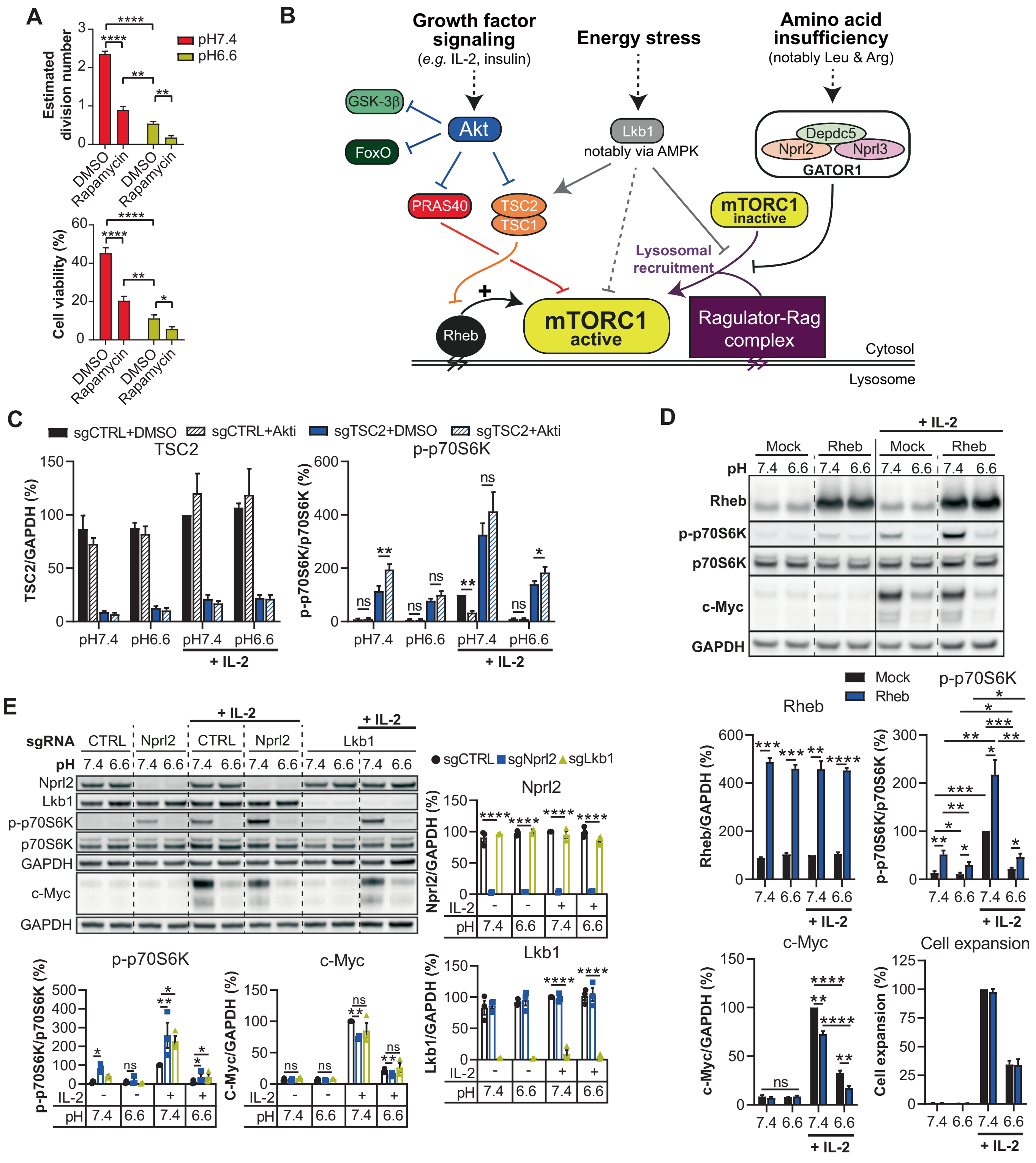
A
Estimated division number
pH7.4
pH6.6
DMSO
Rapamycin
Cell viability (%)
B
Growth factor signaling
(e.g. IL-2, insulin)
Energy stress
Amino acid insufficiency
(notably Leu & Arg)
GSK-3β
Akt
FoxO
Lkb1
notably via AMPK
Depdc5
Nprl2
Nprl3
GATOR1
PRAS40
TSC2
TSC1
mTORC1 inactive
Lysosomal recruitment
Rheb
mTORC1 active
Ragulator-Rag complex
Cytosol
Lysosome
C
sgCTRL+DMSO
sgCTRL+Akti
sgTSC2+DMSO
sgTSC2+Akti
TSC2
TSC2/GAPDH (%)
p-p70S6K
p-p70S6K/p70S6K (%)
+ IL-2
D
Mock
Rheb
Rheb
p-p70S6K
p70S6K
c-Myc
GAPDH
Rheb/GAPDH (%)
c-Myc/GAPDH (%)
Cell expansion
Cell expansion (%)
E
sgRNA
CTRL
Nprl2
Lkb1
sgCTRL
sgNprl2
sgLkb1
Nprl2/GAPDH (%)
Lkb1/GAPDH (%)
C-Myc/GAPDH (%)

 © The Author(s)

Figure EV3. mTORC1 pathway exploration.

(**A**) mTORC1 inhibition leads to proliferation and viability defects following IL-2 stimulation. OT-I CTLs were cultured for three days in the presence ("Rapamycin"), or absence ("DMSO"), of rapamycin 10 nM with exogenous murine IL-2. Results show the estimated division number, or viability, + SEM of three biological replicates from two independent experiments. ****$P < 0.0001$. Division number: pH 7.4+Rapa vs pH 6.6 + DMSO **$P = 0.0021$, pH 6.6+Rapa vs pH 6.6 + DMSO **$P = 0.0020$. Viability: pH 7.4+Rapa vs pH 6.6 + DMSO **$P = 0.0014$, pH 6.6+Rapa vs pH 6.6 + DMSO *$P = 0.0185$ (one-way repeated measures ANOVA, Tukey post-hoc test). (**B**) Simplified scheme of mTORC1 activity regulation. mTORC1 is recruited to the lysosome via the Ragulator-Rag complex where it can eventually be activated by Rheb. Growth factor signaling leads to Akt activation that phosphorylates PRAS40 and TSC2 allowing to block their capacity to inhibit mTORC1 activity. Amino acid sufficiency prevents GATOR1 complex from impeding mTORC1 recruitment to lysosomes. Energy stress leads to Lkb1 activation that can lower mTORC1 activity via several mechanisms (mostly involving AMPK) including TSC2 activation, Ragulator-Rag inhibition and mTORC1 inhibiting phosphorylation. (**C**) Impact of Akt inhibition on mTORC1 activity in TSC2 knockouts. OT-I x CRISPR/Cas9 CTLs were transduced with retroviruses encoding a negative control, a PRAS40 or a TSC2 sgRNA, and were cultured for 4 h in the presence, or absence ("DMSO"), of Akt1/2 inhibitor (10μM- Akti). Bar graphs show the mean levels normalized to the condition "pH 7.4 + IL-2 sgCTRL DMSO" + SEM of four biological replicates from two independent experiments. ns: not significant. pH 7.4 sgTSC2+Akti vs sgTSC2+DMSO **$P = 0.0037$, pH 7.4 + IL-2 sgCTRL+Akti vs sgCTRL+DMSO **$P = 0.0014$, pH 6.6 + IL-2 sgTSC2+Akti vs sgTSC2+DMSO *$P = 0.0195$ (Student's paired *t* test). (**D**) Rheb overexpression improves mTORC1 activity. OT-I CTLs were transduced with a control (Mock) or a Rheb encoding retrovirus, and were cultured for 4 h (western blot analyses) or 4 days (cell expansion and viability). One representative western blot is shown. Bar graphs show the mean levels normalized to the condition "pH 7.4 + IL-2 Mock" + SEM of four biological replicates from two independent experiments. ns: not significant, ****$P < 0.0001$. Rheb: pH 7.4 Rheb vs Mock ***$P = 0.0001$, pH 6.6 Rheb vs Mock ***$P = 0.0002$, pH 7.4 + IL-2 Rheb vs Mock **$P = 0.0017$. p-p70S6K: pH 7.4 Rheb vs Mock **$P = 0.0099$, pH 6.6 Mock vs pH 7.4 Mock *$P = 0.0445$, pH 7.4 + IL-2 Mock vs pH 7.4 Mock ***$P = 0.0001$, pH 6.6 Rheb vs pH 7.4 Rheb **$p = 0.0019$, pH 7.4 + IL-2 Rheb vs pH 7.4 Rheb **$P = 0.0098$, pH 6.6 Rheb vs pH 6.6 Mock *$P = 0.0181$, pH 6.6 + IL-2 Mock vs pH 6.6 Mock *$P = 0.0143$, pH 6.6 + IL-2 Rheb vs pH 6.6 Rheb *$P = 0.0396$, pH 7.4 + IL-2 Rheb vs Mock *$P = 0.0307$, pH 6.6 + IL-2 Mock vs pH 7.4 + IL-2 Mock ***$P = 0.0002$, pH 6.6 + IL-2 Rheb vs pH 7.4 + IL-2 Rheb **$P = 0.0056$, pH 6.6 + IL-2 Rheb vs pH 6.6 + IL-2 Mock *$P = 0.0125$. c-Myc: pH 7.4 + IL-2 Rheb vs Mock **$P = 0.0032$, pH 6.6 + IL-2 Rheb vs Mock **$P = 0.0053$. (Student's paired *t* test). (**E**) Nprl2 and Lkb1 knockouts do not improve mTORC1 activity at low pH. OT-I x CRISPR/Cas9 CTLs were transduced with retroviruses encoding a negative control, a Nprl2 or a Lkb1 sgRNA, and were cultured for 4 h. One representative western blot from two membranes of the same samples is shown. Bar graphs show the mean levels normalized to the condition "pH 7.4 + IL-2 sgCTRL" + SEM of three biological replicates (except for conditions pH 7.4 and pH 6.6 sgLkb1 without IL-2: two) from two independent experiments. ns: not significant, ****$P < 0.0001$. p-p70S6K: pH 7.4 sgNprl2 vs sgCTRL *$P = 0.0296$, pH 7.4 + IL-2 sgNprl2 vs pH 7.4 + IL-2 sgCTRL **$P = 0.0050$, pH 7.4 + IL-2 sgLkb1 vs pH 7.4 + IL-2 sgCTRL *$P = 0.0429$, pH 6.6 + IL-2 sgNprl2 vs pH 6.6 + IL-2 sgCTRL *$P = 0.0339$, pH 6.6 + IL-2 sgLkb1 vs pH 6.6 + IL-2 sgCTRL *$P = 0.0187$. c-Myc: pH 7.4 + IL-2 sgNprl2 vs sgCTRL **$P = 0.0042$, pH 6.6 + IL-2 sgNprl2 vs sgCTRL **$P = 0.0023$. (Student's paired *t* test).

© The Author(s)

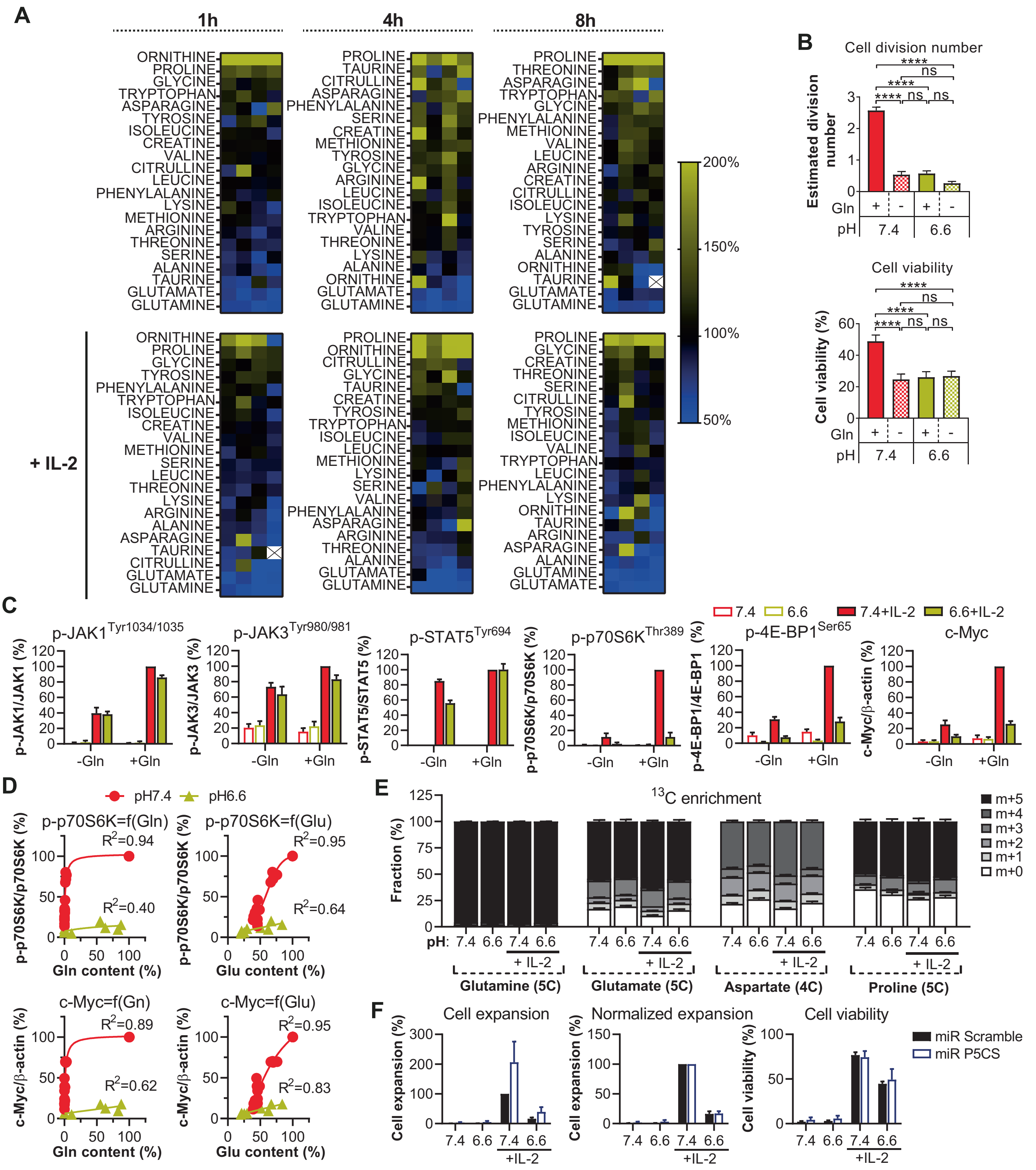
A
1h
4h
8h
ORNITHINE PROLINE GLYCINE TRYPTOPHAN ASPARAGINE TYROSINE ISOLEUCINE CREATINE VALINE CITRULLINE LEUCINE PHENYLALANINE LYSINE METHIONINE ARGININE THREONINE SERINE ALANINE TAURINE GLUTAMATE GLUTAMINE
PROLINE TAURINE CITRULLINE ASPARAGINE PHENYLALANINE SERINE CREATINE METHIONINE TYROSINE GLYCINE ARGININE LEUCINE ISOLEUCINE TRYPTOPHAN VALINE THREONINE LYSINE ALANINE ORNITHINE GLUTAMATE GLUTAMINE
PROLINE THREONINE ASPARAGINE TRYPTOPHAN GLYCINE PHENYLALANINE METHIONINE VALINE LEUCINE ARGININE CREATINE CITRULLINE ISOLEUCINE LYSINE TYROSINE SERINE ALANINE ORNITHINE TAURINE GLUTAMATE GLUTAMINE
+ IL-2
ORNITHINE PROLINE GLYCINE TYROSINE PHENYLALANINE TRYPTOPHAN ISOLEUCINE CREATINE VALINE METHIONINE SERINE LEUCINE THREONINE LYSINE ARGININE ALANINE ASPARAGINE TAURINE CITRULLINE GLUTAMATE GLUTAMINE
PROLINE ORNITHINE CITRULLINE GLYCINE TAURINE CREATINE TYROSINE TRYPTOPHAN ISOLEUCINE LEUCINE METHIONINE LYSINE SERINE VALINE PHENYLALANINE ASPARAGINE ARGININE THREONINE ALANINE GLUTAMATE GLUTAMINE
PROLINE GLYCINE CREATINE THREONINE SERINE CITRULLINE TYROSINE METHIONINE ISOLEUCINE VALINE TRYPTOPHAN LEUCINE PHENYLALANINE LYSINE ORNITHINE TAURINE ARGININE ASPARAGINE ALANINE GLUTAMINE GLUTAMATE
200%
150%
100%
50%
B
Cell division number
Estimated division number
Cell viability
Cell viability (%)
**** ns **** **** ns ns
Gln + - + -
pH 7.4 6.6
C
7.4 6.6 7.4+IL-2 6.6+IL-2
p-JAK1 Tyr1034/1035
p-JAK1/JAK1 (%)
p-JAK3 Tyr980/981
p-JAK3/JAK3 (%)
p-STAT5 Tyr694
p-STAT5/STAT5 (%)
p-p70S6K Thr389
p-p70S6K/p70S6K (%)
p-4E-BP1 Ser65
p-4E-BP1/4E-BP1 (%)
c-Myc
c-Myc/β-actin (%)
-Gln +Gln
D
pH7.4 pH6.6
p-p70S6K=f(Gln)
R²=0.94
R²=0.40
p-p70S6K=f(Glu)
R²=0.95
R²=0.64
p-p70S6K/p70S6K (%)
c-Myc=f(Gn)
R²=0.89
R²=0.62
c-Myc=f(Glu)
R²=0.95
R²=0.83
c-Myc/β-actin (%)
Gln content (%)
Glu content (%)
E
13C enrichment
Fraction (%)
m+5 m+4 m+3 m+2 m+1 m+0
pH: 7.4 6.6 7.4 6.6
+ IL-2
Glutamine (5C)
Glutamate (5C)
Aspartate (4C)
Proline (5C)
F
Cell expansion
Cell expansion (%)
Normalized expansion
Cell viability
Cell viability (%)
miR Scramble
miR P5CS
7.4 6.6 7.4 6.6
+IL-2

 © The Author(s)

Figure EV4. Exploration of glutamine metabolism at low pH.

(**A**) Heat map of intracellular amino acid contents as a function of pH, time, and IL-2 stimulation. Intracellular levels of amino acids were quantified from OT-I CTLs. Results show the percent level of amino acid as compared to the matched pH 7.4 condition using a color code, as outlined on the ladder. Each square shows one biological replicate, with a total of four from two independent experiments. White square with a cross inside means the amino acid was not detected. (**B**) Glutamine deprivation inhibits CTL proliferation and viability. OT-I CTLs were cultured for three days with exogenous murine IL-2 in the presence, or absence, of glutamine (under the glutamax form, 4 mM). Results show the estimated division number, or cell viability, + SEM of at least four biological replicates from at least two independent experiments. ns: not significant, *$P < 0.05$, ****$P < 0.0001$ (one-way repeated measures ANOVA, Tukey post-hoc test). (**C**) Glutamine deprivation lowers mTORC1 activation and c-Myc accumulation. OT-I CTLs were cultured for 4 h in the presence, or absence, of glutamine (2 mM). Results show the mean levels normalized to the condition "pH 7.4 + IL-2" ± SEM of four biological replicates from two independent experiments. (**D**) mTORC1/c-Myc as a function of intracellular levels of glutamine/glutamate. OT-I CTLs were cultured for 4 h with exogenous murine IL-2 in the presence of various exogenous quantities of glutamine. Results show the individual values of p-p70S6K or c-Myc as a function of intracellular amino acid content normalized to the condition "pH 7.4 + 2000 μM Gln" from four biological replicates out of two independent experiments. A correlation curve and R^2 for each pH is displayed. (**E**) Intracellular glutamine, glutamate, aspartate and proline are coming from extracellular glutamine. OT-I CTLs were cultured for 4 h with isotopic ^{13}C-glutamine (2 mM). Results show isotopologue distribution of the indicated intracellular amino acid + SEM of four biological replicates from two independent experiments. The maximum number of carbons that can be labeled is indicated under bracket aside each amino acid. (**F**) P5CS knockdown does not restore CTL proliferation and viability at low pH. OT-I CTLs transduced with a control miR (Scramble) or a P5CS-targeting miR were cultured for 3 days. Bar graphs display mean CTL expansion normalized to the condition "pH 7.4 + IL-2", mean CTL expansion normalized to the condition "pH 7.4 + IL-2" per corresponding miR, or mean CTL viability + SEM of four biological replicates from two independent experiments.

© The Author(s)

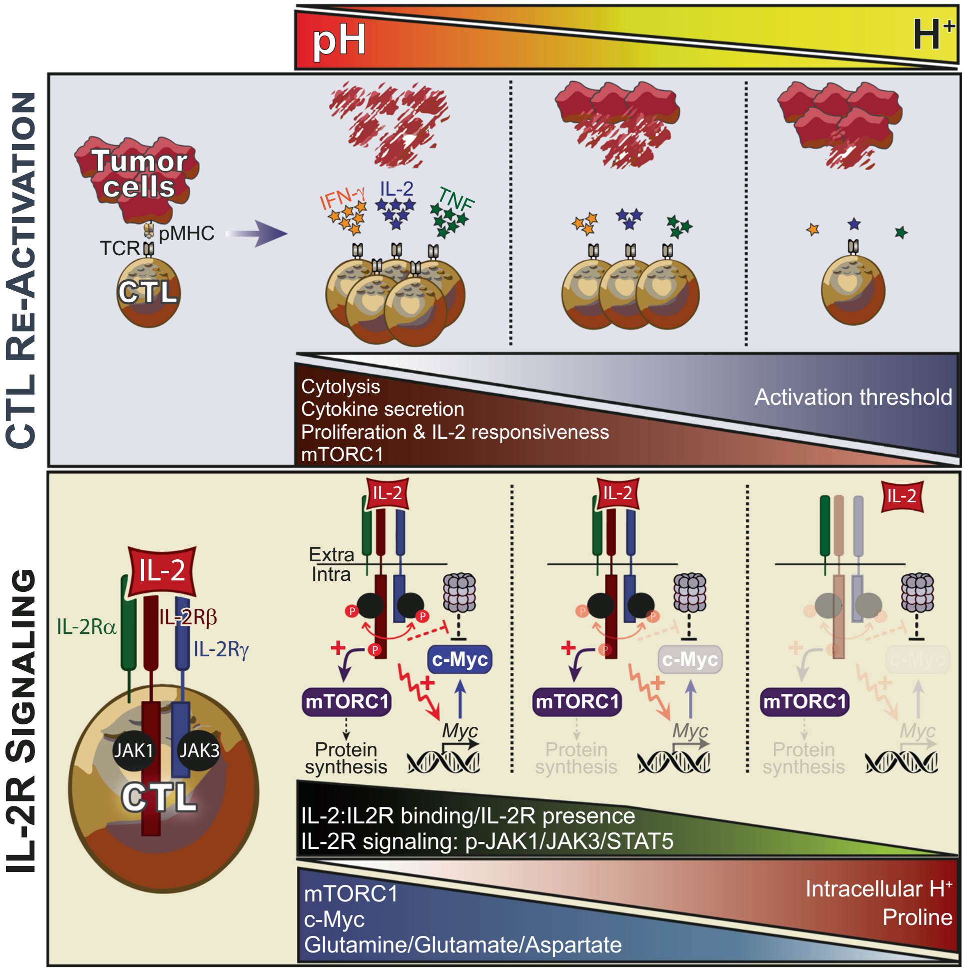

Figure EV5. Schematic overview of the impact of increasingly acidic conditions on CTLs.

 © The Author(s)
